# Epigenomic Consequences of Immortalized Plant Cell Suspension Culture

**DOI:** 10.1371/journal.pbio.0060302

**Published:** 2008-12-09

**Authors:** Milos Tanurdzic, Matthew W Vaughn, Hongmei Jiang, Tae-Jin Lee, R. Keith Slotkin, Bryon Sosinski, William F Thompson, R. W Doerge, Robert A Martienssen

**Affiliations:** 1 Cold Spring Harbor Laboratory, Cold Spring Harbor, New York, United States of America; 2 Department of Statistics, Northwestern University, Evanston, Illinois, United States of America; 3 Department of Horticultural Science, North Carolina State University, Raleigh, North Carolina, United States of America; 4 Departments of Plant Biology, Genetics, and Crop Science, North Carolina State University, Raleigh, North Carolina, United States of America; 5 Departments of Statistics and Agronomy, Purdue University, West Lafayette, Indiana, United States of America; The Babraham Institute, United Kingdom

## Abstract

Plant cells grown in culture exhibit genetic and epigenetic instability. Using a combination of chromatin immunoprecipitation and DNA methylation profiling on tiling microarrays, we have mapped the location and abundance of histone and DNA modifications in a continuously proliferating, dedifferentiated cell suspension culture of *Arabidopsis*. We have found that euchromatin becomes hypermethylated in culture and that a small percentage of the hypermethylated genes become associated with heterochromatic marks. In contrast, the heterochromatin undergoes dramatic and very precise DNA hypomethylation with transcriptional activation of specific transposable elements (TEs) in culture. High throughput sequencing of small interfering RNA (siRNA) revealed that TEs activated in culture have increased levels of 21-nucleotide (nt) siRNA, sometimes at the expense of the 24-nt siRNA class. In contrast, TEs that remain silent, which match the predominant 24-nt siRNA class, do not change significantly in their siRNA profiles. These results implicate RNA interference and chromatin modification in epigenetic restructuring of the genome following the activation of TEs in immortalized cell culture.

## Author Summary

Cultured plant cells are genetically and epigenetically unstable. We investigated the epigenomic consequences of long-term plant cell culture and found that some genes show an increase in DNA methylation, reminiscent of immortalized animal cell lines and cancer cells. By contrast, in the heterochromatic portion of the genome, some transposable elements (TE), undergo dramatic and very precise loss of DNA methylation and transcriptional activation. The reactivated TEs in culture are also accompanied by a production of 21-nucleotide (nt) small RNAs. In contrast, TEs that remain methylated and silent retain the predominant class of 24-nt small RNAs, and do not change significantly in their small RNA profiles. Our results implicate RNA interference in epigenetic restructuring of the genome following the activation of TEs in immortalized cell culture.

## Abstract 3

Immortalized plant cells undergo epigenomic changes, reminiscent of immortalized animal cell lines and cancer cells, accompanied by shifts in small RNA classes, implicating a role for the RNAi machinery in regulating the epigenome.

## Introduction

More than half a century has passed since the concept and practice of plant cell culture was first introduced [1]. Unlike most animal cells, plant cells can change from one differentiated state, representing a committed developmental program, to a completely different one via a transition through a dedifferentiated state typical of callus tissue [2]. This process is achieved by varying concentrations and relative proportions of two major plant growth regulators (auxin and cytokinin) in the growth medium [3]. Under appropriate conditions, callus cells can continue to grow in “immortalized” suspension cultures, which can be maintained indefinitely without differentiation. Plant cells grown in culture exhibit unprecedented levels of genetic and epigenetic instability [1]. Through changes in gene activity, plant cells are able to respond to the challenges presented by tissue culture conditions and continue to divide according to internal and external cues. Epigenetic regulation plays an important role. For example, hormone habituation is a process during which plant cells in culture shift their requirement for exogenous growth regulators between the auxo- and autotrophic states [4]. The epigenetic nature of this shift is demonstrated by the fact that upon plant regeneration from habituated cell culture and reculturing, the original requirement for growth factors is present again in the reestablished cell culture. In addition to the reversibility, this switch occurs at rates that are orders of magnitude higher than mutations rates in plants [5].

The culturing of plant cells induces heritable variation known as somaclonal variation. It has been observed in numerous instances for a variety of plant species, and given the early promise of cell culture for advances in agriculture, the stochastic nature of somaclonal variation poses a significant challenge [6,7]. Genetic analyses of somaclonal variants in maize, rice, tobacco, and other plant species identified novel transposon insertions into genes as an important cause of somaclonal variation [8–14]. Additionally, heritable epialleles are known to occur in plants [15–18], and there is recent evidence that meiotically heritable epialleles with high reversion rates can be induced in plant cell culture [19]. Plant cells in culture undergo changes in both DNA methylation of the repetitive portions of plant genomes [20–24], as well as at individual TEs [25,26]. Reactivation of retrotransposon *Athila*, a highly repetitive component of the pericentromeric heterochromatin in Arabidopsis thaliana [27], was also reported in a cell suspension culture of continuously dividing dedifferentiated *Arabidopsis* cells [28,29]. TEs can serve as insertional mutagens of plant genomes, whereas widespread activation can lead to a wide array of chromosomal rearrangements. These rearrangements can, in turn, lead to misregulation of genes and transposons [1].

Plant genomes contain numerous and diverse TEs [30,31], most of which are transcriptionally silent [27]. In Arabidopsis thaliana, and in other plants, silencing is achieved by both DNA methylation as well as by histone deacetylation and H3 lysine 9 dimethylation, which is targeted by small RNAs (sRNAs) in the 21–24-nucleotide (nt) range [32–34]. In addition, this mutually enforcing set of chromatin modifications allows for the silenced states of TEs to be inherited throughout normal cell divisions and plant development. When silencing is perturbed by mutations in DNA methyltransferases, RNA interference (RNAi) machinery, or histone modifiers, TEs can become reactivated [32,35–38].

With the advent of chromatin immunoprecipitation (ChIP), microarray and deep sequencing technologies, it is now possible to map DNA and histone modifications genome-wide [39–41]. In animal systems, these approaches have uncovered histone modifications specific to promoters, such as histone H3 trimethylation at lysine4 (H3K4me3), to gene coding regions H3K4me2 and H3K36me3, to polycomb-regulated genes (H3K27me3), and to repeats and TEs (H3K9me3 and H4K20me3) [42–44]. This epigenomic information has been used to describe chromatin states specific for different stages of differentiation from pluripotent embryonic stem cells, as well as in cancer cell lines [45–48].

Comparable chromosome-wide and genome-wide epigenomic profiles in plants provided the first glimpses into the plant epigenome. DNA methylation in *Arabidopsis* has a bimorphic distribution: dense cytosine methylation in all three sequence contexts (CpG, CpNpG, and CpNpN) characterizes TEs and other repetitive DNA, whereas short clusters of CpG methylation are found in over 25% of gene coding regions [32,49–51]. The role of euchromatic, genic DNA methylation is not known, but it is extremely variable between accessions of Arabidopsis thaliana and, as a rule, is not accompanied by heterochromatic histone H3K9 dimethylation or the presence of sRNAs [51]. Additionally, the coding regions of *Arabidopsis* genes are enriched in H3K4me2 and H3K27me3 [32,52,53]. In contrast, heterochromatic transposon DNA methylation is strongly correlated with histone H3K9me2 and associated small interfering RNA (siRNA) [32,54].

Beyond a role in transposon silencing, epigenetic modifications play a role in plant gene regulation and development. Gene imprinting in the endosperm [55] and the regulation of vernalization [56,57] are examples of epigenetic control in plant development that are regulated by DNA and histone methylation, respectively. However, epigenetic changes that occur in rapidly dividing dedifferentiated cells during plant cell culture have not yet been described.

Using a combination of ChIP and DNA methylation profiling on tiling microarrays, and high-throughput sRNA sequencing, we have mapped the location and abundance of histone and DNA modifications in rapidly dividing, dedifferentiated *Arabidopsis* suspension cells, and compared them with gene expression in suspension cells and in callus tissue. We have found dramatic DNA hypomethylation and activation of specific TEs, as well as hypermethylation in genic regions that may be responsible for the emergence of epialleles. DNA methyltransferase and most other epigenetic modifiers are up-regulated in cell culture, indicating that loss of targeting rather than modification is responsible for TE activation. Gain of 21-nt siRNA and associated expression of the *Argonaute2* gene family member may accompany this loss of targeting.

## Results

### Chromatin Maps of Plant Cell Suspension Culture

Chromosome-wide maps of DNA and histone modifications were created by microarray hybridization to a custom *Arabidopsis* chromosome 4 tiling array [51]. The 22,761 probes interrogate most of *Arabidopsis* chromosome 4, including the repetitive pericentromeric heterochromatin, as well as the heterochromatic knob on the short arm of chromosome 4 [39]. We profiled covalent modifications of DNA and histones as well as sRNA populations in cultured suspension cells (Figure S1) of the Columbia ecotype that have been in continuous propagation for several years (I. Meier, unpublished data). The cells were cultured in 1× B5 medium with minimal organics supplemented with 3% sucrose and 1.1 mg/l 2,4-D with a 1:1 and 1:9 final dilution for the 16-h (cells7) and the 96-h (cells4) culture regimens, respectively, every 7 d. Flow sorting of DAPI-stained nuclei at 96 h postculture split showed that a vast majority of these cells were diploid and in the G1 stage of the cell cycle (Figure S1). In addition, BrdU incorporation results showed that the cells7 sample had close to three times higher BrdU incorporation relative to the cells4 sample (Figure S1). We also tested the ability of this long-term cell suspension culture to redifferentiate into shoots by growing microcalli from the suspension culture on solid shoot-inducing medium. In contrast to freshly induced calli, which regenerated shoots after 6 wk under the same conditions, cell suspension culture-derived calli continued to proliferate without any differentiation or shoot regeneration.

**Figure 1:**
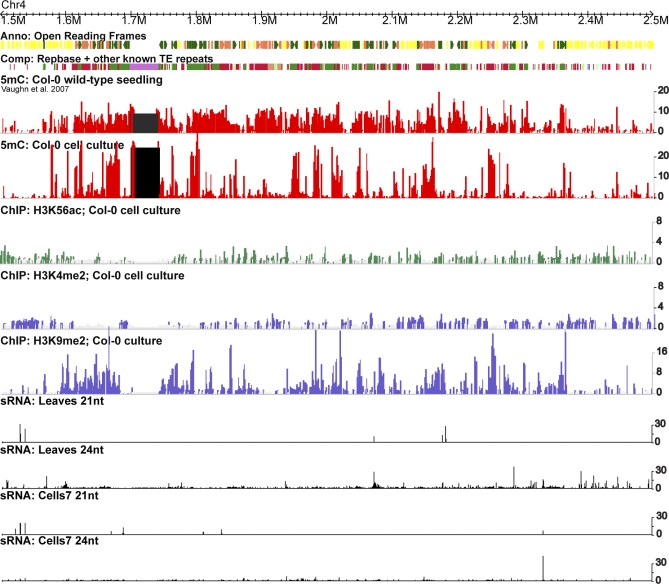
Heterochromatic Modification of Chromosome 4 in *Arabidopsis* Cell Suspension Cultures One megabase encompassing the heterochromatic knob on the short arm of chromosome 4 and the flanking euchromatic region are displayed. Genes are depicted in yellow, DNA transposons are in red and orange, and retrotransposons are in green in the annotation (Anno) and Repbase tracks (Comp). DNA methylation profiles of cell suspension culture and the reference Col seedling DNA methylation profile [51] are presented as red bars. Tiles with significant enrichment of H3K4me2 and H3K9me2 are shown in purple, while those tiles associated with H3K56ac are shown in green. A 100-bp–resolution sliding window analysis of sRNA frequencies, corrected for sRNA library size and genomic copy number, for 21- and 24-nt size fractions is shown.

Cytosine methylation was determined by comparing McrBC-digested genomic DNA to an undigested sample of the same DNA by two-color hybridization to the chromosome 4 tiling array [32]. The presence of 5mC was detected as a statistically significant difference in hybridization signal in the untreated sample relative to the McrBC-treated sample, using a linear model that partitions the variation into both biological and technical sources (Text S1). Samples from two different time points during the cell suspension culture cycle of 7 d (cells4 and cells7, as noted above) were analyzed in quadruplicate. Upon testing for differences between the two DNA methylation profiles, almost no change in DNA methylation (false discovery rate [FDR] significance level α = 0.01) occurs during each culture passage. Subsequent statistical analyses benefited from combining these two experiments, thus treating the two DNA samples as biological replicates (Figure 1).

ChIP-chip was performed by hybridization of DNA immunoprecipitated with antibodies against H3K4me2, H3K9me2, and histone H3 acetylated at lysine56 (H3K56ac) [58]. Each immunoprecipitated and amplified sample was hybridized in triplicate against the input DNA sample, using two-color array hybridization. Statistical analysis (Text S1) identified tiles with significant enrichment of a particular histone modification (Figure 1).

We found significant genome-wide hypomethylation of heterochromatic DNA in cell suspension culture (Figure 1) as compared to Columbia seedling DNA [51]. Significant differences in DNA methylation were also seen in the euchromatic portions of chromosome 4, in which a gain of methylation in cell suspension culture was the most prevalent trend, typically within the transcribed regions of genes (“gene-body methylation”) where CpG methylation occurs.

### Specific Families of TEs Lose Heterochromatic Modifications and Are Transcriptionally Activated in Cultured Cells

The chromatin maps of the heterochromatic portion of chromosome 4 showed that significant loss of DNA methylation occurred in cell suspension culture relative to differentiated leaf cells (Figure 1). Although very few regions lost DNA methylation completely, a majority of TEs that comprise heterochromatin became hypomethylated (Figure 1). Contrary to gene-body DNA methylation, which is correlated with the presence of H3K4me2, heterochromatic DNA methylation in *Arabidopsis* is associated with H3K9me2 and with the presence of sRNAs, which direct this histone modification. DNA hypomethylation, loss of H3K9me2, and in most cases, gain of H3K4me2, were specific to families of TEs. Most retrotransposons, including *Athila*, and *Copia* elements, as well as DNA transposons such as helitrons, *AtMu* elements, and a few *Vandal* and *AtENSPM* elements, lose heterochromatic marks irrespective of their location on the chromosome. In contrast, gypsy class *Atlantys* and *AtGP* long terminal repeat (LTR) retrotransposons and other DNA transposons such as most of the *Vandal* and *AtENSPM* elements retain heterochromatic marks (Figure S2). This observation is particularly striking when two repetitive elements are found juxtaposed with a sharp boundary between the loss and retention of heterochromatic marks corresponding to the boundaries of coding regions of the TEs (Figure 1).

Transcriptional reactivation of TEs was analyzed by microarray analysis using Affymetrix *Arabidopsis* ATH1 microarrays. Cells7 samples were compared with 2-wk-old seedlings grown on plates under standard conditions, and 6-wk-old *Arabidopsis* calli generated from cotyledons grown on MS plates supplemented with a combination of cytokinin and auxin at callus-inducing concentrations. ATH1 arrays, in addition to bona fide genic probes, have close to 1,200 probe sets specific to various *Arabidopsis* TEs, of which approximately 200 map to chromosome 4 (R. K. Slotkin and R. A. Martienssen, unpublished data). A majority of oligonucleotide probes on this array are specific to a single TE, or a small number of closely related TEs, which allowed for precise mapping of TE transcripts to their chromosomal location(s).

**Figure 2:**
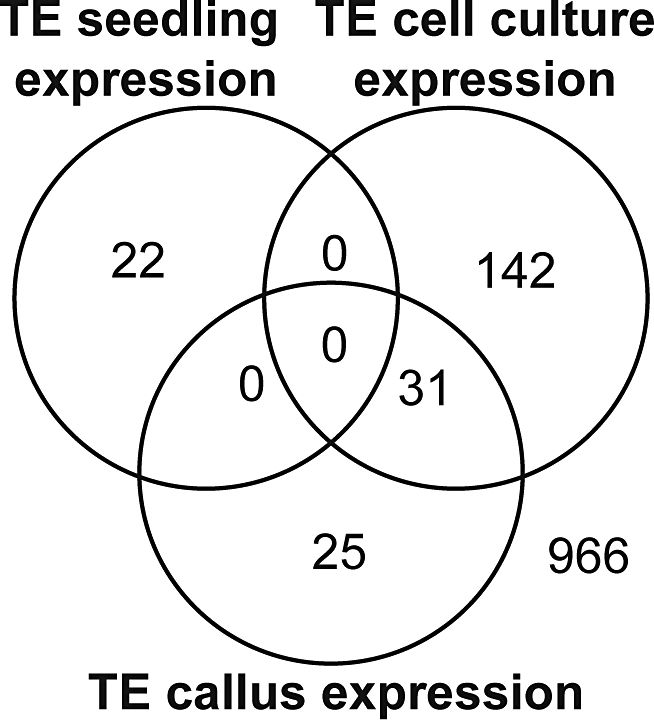
TE Expression Differences between Cell Suspension Culture, Seedling, and Callus Tissues A Venn diagram of transcripts with significant TE homology that show significant overaccumulation (normalized value ≥ 1.5) in ATH1-based microarray expression profiles of cell suspension culture, seedling, and callus samples. There is no overlap between samples and a much higher degree of TE activity in cell suspension culture.

Weak expression of a few TEs was seen in callus, but a strong up-regulation of a much larger number of TEs was found in suspension cells, relative to both seedling and callus samples (Figure 2). A total of 142 (12%) out of 1,155 TE-specific probes on the array were up-regulated 1.5-fold or higher in cell suspension culture. Only 22 and 56 TEs (<2% and <5% of the total TE probes) were expressed in seedling or callus samples, respectively (Figure 2), none of which were in common with cell suspension samples. The transcriptional reactivation of selected TEs located on *Arabidopsis* chromosome 4 was confirmed by reverse-transcription PCR (RT-PCR) (Figure S2).

### Small RNA Profiles in Suspension Cell Culture

**Figure 3:**
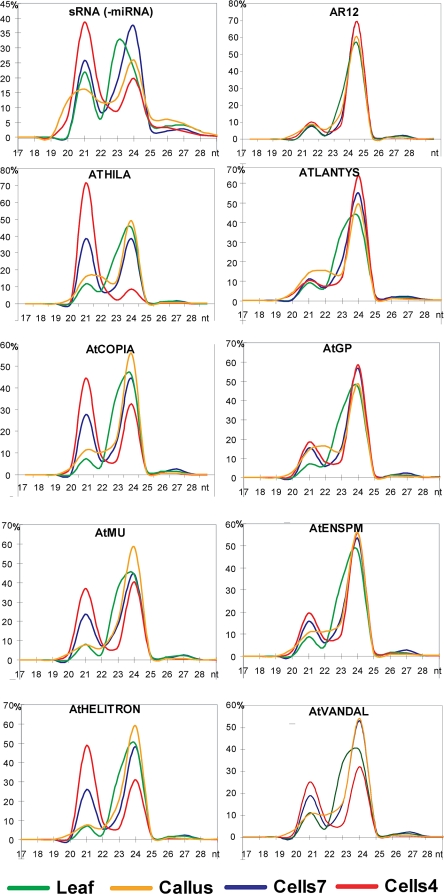
Size Distribution of sRNA Sequences in Cell Suspension Culture (Cells4 and Cells7), Col-0 Leaves, and Col-0 Callus The relative frequencies of all sRNAs (with all known miRNA sequences removed) according to their size for each of the small RNA libraries, as well as only for those sRNAs matching particular families of *Arabidopsis* repeats (*AR12*, *Athila*, *Atlantys*, *Helitron*, *AtGP1*, *AtMu*, *AtENSPM*, *AtCopia*, and *AtVandal*).

We further investigated the specificity of epigenomic changes in *Arabidopsis* cell suspension culture heterochromatin by sequencing sRNA from the two cell suspension culture samples (cells7 and cells4), as well as from leaf and callus samples. In total, we sequenced 1,910,836 and 3,075,388 sRNAs matching the *Arabidopsis* Col-0 reference genome from the cells7 and cells4 libraries, respectively, as well as 1,737,719 and 2,070,923 sRNAs from the leaf and callus libraries, respectively. (Table S1). We tested the technical reproducibility by resequencing the cells4 library, which showed an excellent match in relative size frequency distribution (unpublished data).The overall size distribution of siRNAs from the four libraries showed a typical distribution with the relative frequency maxima at 21 nt and 24 nt, when known microRNAs (miRNAs) were removed from the libraries (Figure 3). Whereas the 24-nt class was the most abundant size class, an overall decrease in the relative frequency of 24-nt sRNAs was noted in the cell suspension culture library, cells4. This prompted further analysis of the sRNA data to identify genomic regions responsible for this reduction.

Each sRNA sequence from the dataset was associated with a particular Repbase repeat class representing all the major families of TEs in the *Arabidopsis* genome, and we examined the size distribution of those classes of sRNAs (Figure 3). This analysis showed that when we compared the relative size frequency of sRNAs specific to particular TE families (Repbase repeat classes), two patterns emerged. The sRNAs specific to those TEs that lose heterochromatic marks and become transcriptionally reactivated in cell suspension culture (*Athila*, *AtCopia*, *AtMu*, and *Helitron* elements) showed a major shift in relative frequency from 24 nt to 21 nt in the cells4, but also in cells7 libraries, relative to sRNA profiles of the leaf and callus libraries (Figure 3). In contrast, the sRNAs specific to those TE families that do not lose the heterochromatic marks and remain transcriptionally silent in cell suspension culture (*Atlantys*, *AtGP*, *Vandal*, and *AtENSPM*) retain the relative sRNA size distribution found in the leaves and callus (Figure 3).

**Figure 4:**
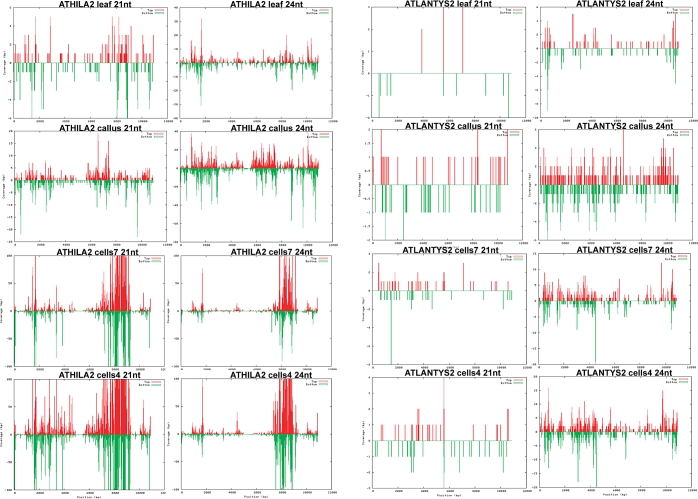
Spatial Distribution and Frequency of sRNA Sequences for *Athila* and *Atlantys* Retrotransposons in the Leaf, Callus, and the Cell Suspension Culture (Cells7 and Cells4) Libraries A representative full-length *Athila2* and *Atlantys2* TE from chromosome 4 was used as a sequence-matching template to map 21-nt and 24-nt sRNAs with perfect matches, resulting in single-nucleotide coverage of the template sequences. The coverage was plotted on the *y-*axis, for both DNA strands (top strand in red and the bottom strand in green), ranging from 0 to 6, 20, 100, and 100 for *Athila2* 21-nt matches, respectively, and from 0 to 40, 40, 100, and 100 for the *Athila2* 24-nt matches; and from 0 to 3, 2, 4, and 4 for the *Atlantys2* 21-nt matches, and from 0 to 6, 6, 15, and 20 for *Atlantys2* 24-nt matches, respectively. The *x*-axis represents the template sequence in the 5′ to 3′ direction, ranging from 0 to 12,000 bp.

We also examined the spatial distribution of sRNAs in cell suspension culture by mapping sRNAs to representative full-length elements representing each of the TE classes listed above. Whereas the distribution and abundance of sRNAs matching a selection of *Atlantys2* (Figure 4), *AtGP1*, and *Vandal14* elements (Figure S3) on chromosome 4 did not change significantly in the four sRNA libraries, a major shift was observed in both the size and location of sRNAs mapping to *Athila2* (Figure 4). Importantly, the overrepresented 21-nt *Athila* sRNAs (and the 24-nt sRNAs to a lesser extent) were localized to the coding regions of this element (Figure 4). In addition, the change in relative size frequencies of *Athila* sRNAs observed in cell suspension culture (Figure 3) was found to be due to massive up-regulation in the production of 21-nt sRNAs, rather than a net loss of 24-nt-size sRNAs (Figure 4). The same patterns were observed in four other full-length *Athila* elements located on chromosomes 1, 2, 3, and 5 (unpublished data). Similarly, a *Helitron2* element on chromosome 4 was found to gain 21-nt sRNAs specifically at the 3′ end of the coding region (Figure S3) without a loss of 24-nt sRNAs. The only exception to this general trend was the small family of *AtMu* elements in which the increase in the relative frequency of 21-nt sRNAs was accompanied by a significant loss of the 24-nt sRNAs in cell suspension culture (Figure S3).

One way that TE families might be differentially regulated by sRNAs is through interaction with different *Argonaute* proteins, which in turn, is determined by the 5′ nucleotide and the sRNA size [59,60]. We, therefore, compared the first nucleotide frequencies by size, origin, and TE specificity (Figure S4). Size, distribution, and 5′ nucleotide specificity were found to be unchanged in leaves and callus, with the majority of TE sRNAs having A as their 5′ base (Figure S4). However, in the cell suspension culture libraries, the frequency of 5′ A sRNAs is increased in every class of TE that we tested, irrespective of the sRNA size (Figure S4). In addition, sRNAs from TEs that lose heterochromatic silencing also have a decrease in the relative frequencies of sRNAs with 5′ G as their first nucleotide, irrespective of their size. sRNAs specific to *Atlantys*, which remains heterochromatic, have no such shift in 5′ nucleotide preference in cell suspension culture (Figure S4).

### Genic Methylation Is Retained in Cell Culture and Is Correlated with Histone H3K4me2 Modification Whereas H3K56ac Marks the Proximal Promoter Regions

**Figure 5:**
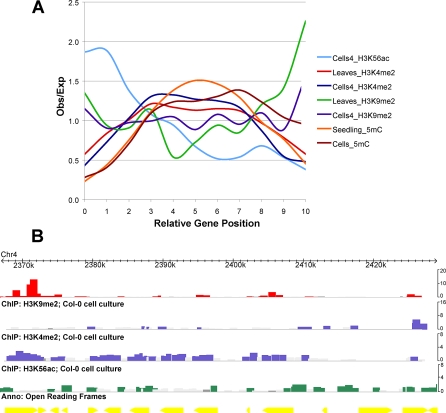
Euchromatic Modification of Chromosome 4 in *Arabidopsis* Cell Suspension Cultures (A) The number of genic array tiles with no repeat homology detecting significant methylation, H3K56ac, H3K9me2, and H3K4me2 in suspension cell culture was calculated at 10% intervals relative to the length of each gene, and compared with the number expected if these modifications were randomly distributed. Parallel computations were done for leaf H3K4me2 and H3K9me2, and data for seedling DNA methylation were imported from previously published work [51]. (B) Chromatin maps of an approximately 87-kb region of euchromatin from the short arm of chromosome 4. Whereas genic DNA methylation patterns are similar between the cell suspension culture and seedlings [51], H3K9me2 is almost completely lacking from this region. Instead, gene coding regions are enriched in H3K4me2, and this pattern is interrupted by H3K56ac-enriched promoter regions preceding the coding regions in an intercalated pattern.

In contrast to heterochromatic DNA methylation, which is correlated with transcriptionally silent sequences, gene-body methylation tends to occur within genes that are transcriptionally active [49,51]. To test whether genic DNA methylation coincides with the sequences enriched for H3K4me2, a histone modification characteristic of transcribed regions, the chromosomal distribution of cytosine methylation was compared to H3K4me2 using an “average gene analysis” that utilized all genes represented by multiple tiles and lacking sequence homology to known repetitive elements. For each chromatin modification, the number of tiles detecting enrichment was calculated at 10% intervals relative to the length of each gene, with position 1 being the transcription start site. This was compared with the number expected if distribution of significant features was randomly distributed relative to gene position (Figure 5A). These comparisons revealed that the distributions of 5mC and H3K4me2 are spatially correlated along gene coding regions, such that both epigenetic marks are depleted from the sequences flanking the gene coding regions (positions 0–1 and 9–10 in Figure 5A), whereas they are specifically enriched, relative to expected average chromosomal distribution of each mark, over the open reading frames of the gene coding regions (positions 1–9 in Figure 5A). In fact, 69% of genic tiles with significant DNA methylation in cell suspension culture are also associated with H3K4me2, compared with only 37% of genic tiles without methylation. This correlation is not unique to cell culture-derived chromatin, since a similar correspondence is observed for the distribution of DNA methylation data in seedling [51] and H3K4me2 methylation in leaf tissue (Figure 5A).

In contrast to H3K4me2, H3K9me2 is enriched in intergenic regions (Figure 5A) and is negatively correlated with the presence of gene-body DNA methylation. Instead, it is highly enriched in TEs [32], which are found predominantly in intergenic regions. Strikingly, our “average gene analysis” revealed that H3K56 acetylation was uniquely and strongly enriched 0–100 bp upstream of the coding region (Figure 5A). The association of H3K56ac with gene promoter regions is ubiquitous throughout the chromosome (Figure 5B) and does not seem to be restricted to expressed genes. This modification is thus a useful tool for gene-finding algorithms. It is also interesting to note that TEs that gain H3K4me2 are also associated with H3K56ac at their 5′ ends (Figure 1), indicating they may be actively transcribed.

### Genes Undergo Hypermethylation in Cell Suspension Culture, Which Can Lead to Epiallele Formation

**Figure 6:**
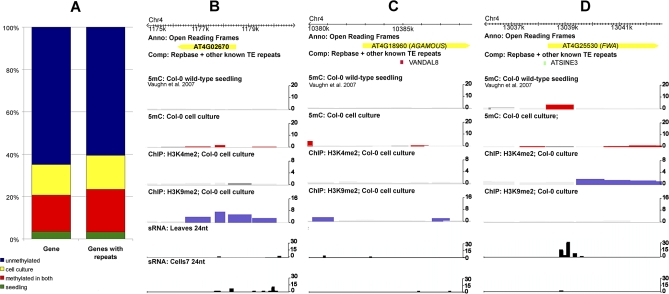
Genic Methylation and Epiallele Formation in *Arabidopsis* Cell Suspension Culture (A) Genic methylation changes between seedling and cell suspension culture (percentage of chromosome 4 array gene tiles). Green: tiles associated with genic sequences that lose DNA methylation in cell suspension culture; yellow: tiles methylated only in cell suspension culture; red: tiles methylated in both samples; and blue: tiles not methylated in either sample. Chromatin maps of cell suspension culture at the (B) *IDD12* (*At4g02670*), (C) *AGAMOUS*, and (D) *FWA* loci. Tiles with DNA methylation are presented as red bars, while those enriched in H3K9me2 and H3K4me2 are shown in purple. Tracks representing the 21-nt and 24-nt sRNA abundance per 100-bp sliding window are shown in black.

Both hypomethylation of transposon sequences and hypermethylation of euchromatic gene sequences can lead to the generation of epialleles at specific loci, which have the potential to heritably transmit the transcriptional state imposed by the methylation status of its sequences [61]. Since hypermethylation of the euchromatic portion of chromosome 4 was observed in cell suspension culture relative to seedling DNA methylation [51], we looked at the distribution of this novel DNA methylation with respect to gene coding regions (Figure 6A). Every tile on chromosome 4 was assigned a category depending on whether its sequence was part of a gene, a gene with an associated repeat, or an intergenic region with no known coding potential, and each tile was then counted as methylated if there was any significant DNA methylation detected at that location. The majority of changes in suspension cell culture euchromatin were due to novel DNA methylation relative to seedling DNA methylation [51] in the genic tile classes (46%), although some genes methylated in seedlings lost methylation in cell suspension culture (12%) (Figure 6A). We tested whether this euchromatic hypermethylation is associated with sRNAs, by comparing the gain or loss of DNA methylation to the frequency of sRNAs from leaves and cell suspension culture from the corresponding genomic regions. We found no significant association between the sRNAs and genic methylation on euchromatic DNA hypermethylation (*p* = 0.29). By contrast, the association of sRNAs and heterochromatic DNA methylation was highly significant (*p* = 0.001). A complete list of genes methylated in either cell suspension culture alone or in both the cell culture and seedling is listed in Table S2.

**Figure 7:**
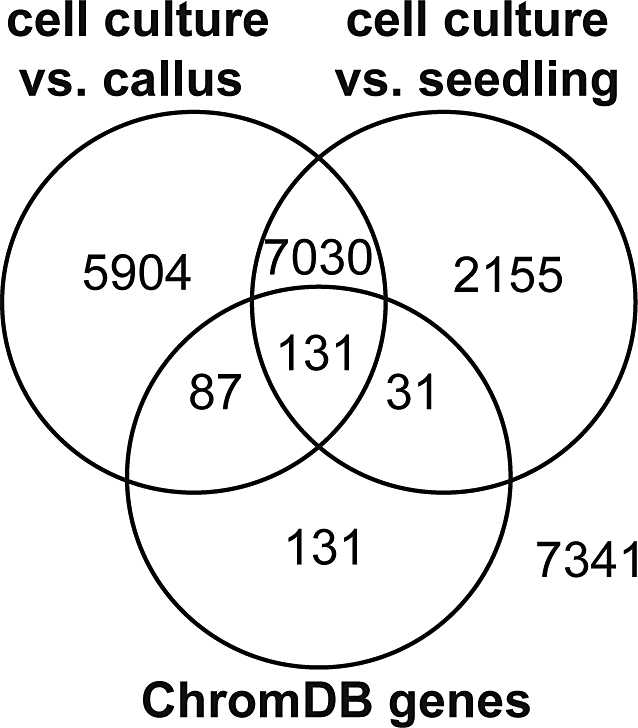
Gene Expression Differences between Cell Suspension Culture and Seedling and Callus Tissues Significant gene expression differences detected by ATH1 microarray expression profiling between cell suspension culture and seedling or callus samples are represented in this Venn diagram. Also, 65% of genes annotated by ChromDB as having a role in chromatin regulation are differentially expressed in these samples relative to seedling.

We also compared the DNA methylation profiles and the genome-wide gene expression levels using Affymetrix ATH1 array. This comparison revealed major changes in gene expression, with 41% of genes exhibiting statistically significant changes (FDR controlled to 0.05),between cell suspension culture and seedling, and 58% between cell suspension culture and callus (Figure 7). However, these expression changes were not correlated with changes in gene-body methylation between cell suspension culture and seedlings.

In search of hypermethylated genes that might be silenced in cultured cells, we found that 14% of genic tiles that become hypermethylated in cell suspension also gained H3K9me2, doubling the number of doubly marked genes found in seedlings. A total of 89% of these tiles were associated with increased sRNA matching these genes, for a total of 28 genes on chromosome 4 (Table S3) that represent candidates for epiallele formation in the immortalized, proliferating cells of the cell suspension culture. An example of a novel epiallele is a member of the IDD family of zinc finger transcription factors At4g02670, which gains both DNA and H3K9 methylation as well as 24-nt sRNAs in cell suspension culture (Figure 6B). Two *Arabidopsis* genes on chromosome 4 are well known to be epigenetically regulated: the floral regulator *AGAMOUS* (*AG*) and the imprinted homeodomain protein gene *FWA*. A short repeat with limited similarity to the *Vandal* class of transposons in the second intron of *AG* is prone to hypermethylation in *ddm1* and *met1* mutants [17], whereas a short interspersed nuclear element (SINE) element within *FWA* 5′ UTR is hypermethylated and targeted by sRNAs [32,62]. *AG* and *FWA* change in their epigenetic status in cell suspension culture, with the second intron of *AG* gaining a low level of DNA methylation. This is accompanied by methylation of H3K9 in the same region and the appearance of sRNAs 24 nt in size (Figure 6C). The imprinted *FWA* gene is normally expressed only from the maternal chromosome during endosperm development [55]. Along with other TEs, the SINE element within the *FWA* 5′ UTR region becomes hypomethylated in cell suspension culture with a concomitant loss of 24-nt sRNA from the same region (Figure 6D). This is accompanied by strong transcriptional reactivation of *FWA* with a 25-fold increase in cell suspension culture relative to seedling expression and by accumulation of H3K4me2 and low levels of DNA methylation in the *FWA* coding region (Figure 6D).

### Genes Involved in Chromatin Regulation Exhibit Cell Culture-Specific Expression Changes

In order to test whether the heterochromatic DNA hypomethylation and changes in histone methylation patterns seen in cell suspension culture may be due to changes in expression of DNA and histone methyltransferases and other genes affecting chromatin modifications, gene expression levels were examined for a list of 380 chromatin regulation-related genes from the ChromDB database (http://www.chromdb.org/). A total of 162 out of 380 genes on this list exhibited significant expression differences between cell suspension culture and Col seedling samples. Of these, 121 were up-regulated and 41 were down-regulated in cell suspension culture (Table S4). DNA cytosine methyltransferases were overexpressed; from a 2-fold increase for *CMT3*, *DRM1*, and *DRM2* to a 5-fold increase for *MET1* (Table S4). The chromatin remodeling gene *DDM1* is also required to methylate TEs, and *DDM1* transcripts were six times more abundant in cell suspension culture. Another *SNF2* class chromatin remodeling gene required for gene silencing, *CLSY1*, was also up-regulated in cell suspension culture 3-fold, but *DRD1* did not change in expression levels. The *VIM1* gene, required to guide CpG DNA methylation during replication, was also up-regulated more than 10-fold. Additionally, the DNA demethylase *ROS1* transcript levels were abolished (Figure S5). In summary, genes involved in DNA methylation are up-regulated in cell suspension culture, whereas those involved in DNA demethylation are down-regulated. This likely contributes to genic hypermethylation, but cannot account for TE activation.

Methylation of H3K9me2 in *Arabidopsis* is under control of histone methyltransferases of the Su(var)3–9 type. The *SUVH4* gene (*KYP*) predominates, but *SUVH5* and *SUVH6* have influence over some heterochromatic sequences. The observed loss of H3K9me2 in cell suspension culture could be explained if the transcripts of H3K9 methyltransferases were down-regulated. However, comparison of transcript levels for these three *SUVH* genes showed that only *SUVH6* was down-regulated in cell culture, whereas the major H3K9 methyltransferase *KYP* was up-regulated 3-fold in this sample relative to seedling, with H3K9me2 remaining ubiquitous at heterochromatic regions of the genome. No significant difference in *SUVH5* transcripts was observed (Table S4). Three putative and closely related H3K9 methyltransferases (*SUVH1*, *SUVH3*, and *SUVH8*) were also up-regulated in cell suspension culture (Table S4). Histone demethylases and *jumonji* domain family members *JMJ22* and *JMJ30* were up-regulated several fold in the cell suspension culture, whereas *JMJ24* was down-regulated. The *LSD1* family of histone demethylase paralogs in *Arabidopsis* (*FLD*, *LDL*, *LDL2*, and *LDL3*) showed no changes in gene expression (Table S4). Histone deacetylases followed the same trend, with *HDA6*, *HDA8*, *HDA9*, *HDA17*, *HDA18*, and *HDA19* up-regulated in cell suspension culture, whereas *HDA14* was the only histone deacetylase down-regulated. Overall, these results indicate that a preponderance of genes involved in heterochromatic modifications increase in expression levels in cell suspension culture relative to seedling.

Changes in the pattern, rather than the extent, of DNA and histone methylation in suspension cell culture might be more readily explained by changes in the targeting machinery, which relies on RNAi. We, therefore, looked at the expression levels of genes from the *Dicer-like*, *RNA-dependent RNA Polymerase*, and *Argonaute* gene families as well as the plant-specific RNA polymerase IV (*NRDP1a*). Whereas genes involved in miRNA processing, such as *AGO1* and *DCL1*, were all either down-regulated in cell suspension culture relative to seedling or did not change in expression, those genes that are part of the heterochromatic silencing sRNA pathway, *AGO4*, *NRPD1a*, *RDR2*, and *DCL3*, were all up-regulated in the cell suspension culture at least 2-fold, with the *NRPD1a* transcripts being 18 times more abundant in cell suspension culture. Interestingly, additional members of the *Argonaute* gene family, *AGO2*, *AGO3*, *AGO5*, and *AGO9*, were up to 30-fold up-regulated in cell suspension culture, potentially accounting for changes in sRNA abundance.

## Discussion

Differentiated plant cells from various species and tissues can be experimentally manipulated using plant growth factors to dedifferentiate in vitro, resulting in callus tissue, from which somatic embryos and plantlets can later emerge. This process often leads to heritable changes in phenotype, referred to as somaclonal variation [7,19,63]. The patterns and modes of inheritance of somaclonal variation point to misregulation of epigenetic control mechanisms [19,24]. Cultured cells can be propagated indefinitely in suspension as continuously dividing cells reminiscent of immortalized animal cell cultures. The epigenomic chromatin maps of a long-term *Arabidopsis* cell suspension culture that we have generated were radically different from comparable maps of *Arabidopsis* seedling tissue [49–51] from which the cultures were originally derived. Whereas heterochromatic DNA becomes hypomethylated in cell suspension culture, the euchromatic portion of chromosome 4, including many genes, becomes hypermethylated instead. However, genes with remnants of TEs in their promoter or coding sequences can also lose methylation in cell suspension culture relative to *Arabidopsis* seedling samples. For example, the imprinted gene *FWA* is reactivated in cell suspension culture accompanied by loss of methylation in the SINE element that constitutes its promoter [32]. Loss of this methylation generates epialleles [62], and is predicted to contribute to somaclonal variation. Patterns of histone modification and siRNA biogenesis may underlie these changes, as described below.

### Euchromatin in Cultured Suspension Cells

We found that most genic DNA methylation is associated with H3K4me2, indicating that gene-body methylation by MET1 might be guided, directly or indirectly, by H3K4me2 (see below).

Gene-body methylation in *Arabidopsis* is dependent on *MET1* [32], is highly variable between *Arabidopsis* accessions, and is positively correlated with elevated gene expression. In support of this idea, the comparison between the distribution of gene-body DNA and histone methylation patterns revealed that H3K4me2 is colocalized with gene-body DNA methylation for over 75% of methylated genes. In yeast, both H3K4me2 and H3K4me3 mark genic regions, but H3K4me3 is characteristic of transcriptionally active genes [64], whereas in plant and mammalian genomes, H3K4me3 is found at promoters [43,65]. In mammalian cells, de novo methylation via DNMT3 complexes cannot be recruited to nucleosomes containing H3K4me3, providing a mechanism. perhaps, for exclusion of DNA methylation from promoters [66].

Whereas H3K4me2 is deposited exclusively within coding regions, we showed that promoters are specifically enriched in H3K56ac while gene coding regions were depleted. In addition to its role in genome stability, the acetylation of Lys56 at histone H3 (H3K56ac) has been shown recently to associate with gene promoter regions in budding yeast [67,68] and in particular with transcriptionally active promoters and the elongating form of RNA Polymerase II in yeast and *Drosophila* [69]. We show for the first time, to our knowledge, that H3K56ac is also present in gene promoter regions in plants, as well as in TEs that have lost heterochromatic marks in cell suspension culture. Acetylation of this particular amino acid located at the first point of contact between the major groove of DNA and the histone octamer may allow access to the core transcriptional machinery [67] as well as replication-coupled nucleosome assembly [70].

Even though most methylated genes are associated with H3K4me2, and are actively transcribed, a small percentage of methylated genes are associated with H3K9me2 in leaves, indicating they are epigenetically silenced. This percentage doubles in cell culture, so that 28 genes from chromosome 4 become hypermethylated and associated with H3K9me2. Close to 90% of these genes are associated with novel 24-nt sRNAs in cell suspension culture, indicating sRNA is the major targeting mechanism for epiallele formation. These genes may also include potential targets of the DNA demethylase ROS1, whose expression is lost in cell culture and is accompanied by hypermethylation of the promoter region of *ROS1* [71], which contains a helitron TE fragment. A third of these genes are significantly down-regulated or silenced and represent good candidates for epigenetically regulated genes affecting various aspects of somaclonal variation and habituation [4,72]. It is important to note that independent plant cell culture lines can vary considerably in their molecular phenotypes. Therefore, analyses of epigenetic changes in independent plant cell culture lines are needed before general conclusions about individual genes can be drawn.

Heterochromatin is activated in cultured suspension cells. In contrast to euchromatin, the pericentromeric heterochromatin of chromosome 4 was massively hypomethylated in cell suspension culture. The majority of these heterochromatic regions consists of various classes of TEs [27], but only some of these classes were hypomethylated, whereas others, such as the LTR retrotransposons *ATLANTYS* and *AtGP*, remained methylated. Loss of DNA methylation was often accompanied by loss of H3K9me2 and in some, but not all cases, gain of H3K4me2. Similar to reactivation of TEs in calli or cell suspension culture of maize, rice, and tobacco [11,14,26,73], as well as *Arabidopsis* [28], we were able to detect transcripts from this group of TEs, though only a subset of the elements with a permissive chromatin landscape was actually transcribed. This very likely reflects the fact that most TEs have undergone genetic changes (mostly deletions and insertions) over evolutionary time, diminishing their coding and transcriptional potential.

These changes are reminiscent of transposon deregulation caused by mutations in *ddm1*, a chromatin remodeling factor [74]. These mutants lose transposon-specific DNA methylation, as well as H3K9me2, accompanied by gain of H3K4me2 [54]. However, *ddm1* mutants affect all families of TEs, whereas cell suspension culture only reactivates specific TEs. Furthermore, 75% of a sample of 380 genes involved in heterochromatin regulation, including *DDM1*, are up-regulated in cell culture, indicating that the silencing machinery is largely intact. Instead, the failure to deliver epigenetic marks to some TEs and not others could be the result of perturbations in targeting these modifications by RNAi and sRNA molecules.

### Small Interfering RNA

While sRNAs accumulated from both silenced and reactivated TEs in cell suspension culture, the relative abundance of different size siRNA changed with respect to differentiated tissues. TEs with the strongest reactivation in cell suspension culture, such as *Athila* retrotransposons, as well as some members of the *Helitron*, *Mu*, *Vandal*, and *En/Spm* families of transposons, gained 21-nt siRNA, but lost 24-nt siRNA only rarely. In contrast, TEs that remained silent in cell suspension culture, such as most *Atlantys* and *AtGP* retrotransposons and some *Vandal* and *EnSpm* TEs, produced the 24-nt siRNAs at the same level as they did in vegetative tissue. It would therefore seem that failure to maintain silencing at heterochromatic sequences in cell suspension culture is associated with a shift in relative abundance of 21-nt siRNAs.

The novel 21-nt TE sRNA we have detected in cultured cells show a preference for adenosine (A) at the 5′ end, and a reduction in guanine (G). These changes are diagnostic of the preferential loading of sRNA onto one or more members of the family of *Argonaute* proteins found in *Arabidopsis*. Although the expression levels of AGO4, the *Argonaute* protein known to bind 24-nt heterochromatic sRNA, did not change in cell suspension culture, four *Argonaute* genes whose roles have not yet been defined, *AGO2*, *AGO3*, *AGO5*, and *AGO9*, are massively up-regulated in cell suspension culture, along with PolIVa, which has been implicated in heterochromatic sRNA biogenesis [75,76]. *Arabidopsis* AGO2 has recently been shown to bind 21-nt siRNAs, with a strong preference for A as the first nucleotide of the siRNA [59,60]. This is precisely the most abundant class of sRNAs in cell suspension culture, and *AGO2* is massively up-regulated in cultured cells. If AGO2 is coupled with the biogenesis of the 21-nt siRNA, then it may preclude the proper targeting by and inclusion of the 24-siRNAs into other AGO complexes, resulting in diminished potential of heterochromatic targeting by sRNAs. Additionally, our data support a minor role for a so-far unidentified AGO protein with a preference for G in targeting H3K9me2 and DNA methylation synergistically with AGO4.

### Reprogramming of Epigenetic Modifications in Immortalized Cultured Cells

Reactivation and hypomethylation of TEs is not unique to plant cell culture. *Drosophila* Kc and S2 cultures harbor numerous actively transcribed *copia* and *copia*-like retrotransposons [77,78] capable of transposition [79,80]. The reactivation seems independent of the origin of the culture and does not depend on DNA methylation, which is absent from *Drosophila* [81]. However, components of the piRNA pathway required to silence several classes of transposons are not expressed in *Drosophila* cell cultures, perhaps accounting in part for this activation [82]. In immortalized cancer cell cultures, non-LTR Alu SINE elements [83,84], as well as LINE-1 elements, lose DNA methylation and are reactivated [85–87]. In mouse, hypomethylation of TEs occurs in mutants in the maintenance DNA methyltransferase DNMT1, and as in plants, leads to their transcriptional reactivation [36,88,89]. In tumors, genome-wide hypomethylation also results in TE activation ([90,91], but tumors express DNMT1 at normal or elevated levels [92–94]. These results indicate that, as in plant suspension cell cultures, targeting rather than maintenance of DNA methylation is defective in mammalian cell cultures and tumors [88]. Whereas the majority of CpG islands in mammalian genomes are normally devoid of methylation [95,96], some CpG islands become hypermethylated in immortalized animal cell cultures [97,98]. This methylation often leads to transcriptional gene silencing [95,96], although loss of genic DNA methylation in cell culture can also occur [99–101]. Both the maintenance and de novo DNA methyltransferases are required for CpG hypermethylation in animal cell cultures [102]. Moreover, there is a decline in expression levels of these genes prior to immortalization, which is accompanied by genome-wide DNA hypomethylation, followed by an overexpression in the immortalized cell culture. The resulting hypermethylation [103] is reflected in the gradual increase in CpG hypermethylation of genes in immortalized animal cells [98,104] during the transition from senescent to immortalized cell culture growth [105,106].

Our results show that in *Arabidopsis* cell suspension culture, the expression levels of *MET1*, the maintenance DNA methyltransferase, as well as those of *CMT3* and *DRM2*, follow the same pattern, with a 4-fold drop in expression levels of *MET1* during callus induction, and a 4-fold increase in expression in cell suspension culture, relative to *MET1* expression in seedlings. A corresponding drop in methylation preceding the establishment of the cell suspension culture, would create a need for de novo targeting of methylation, by CMT3 and DRM2, particularly to repeat-containing genic regions [107]. Mistargeted de novo methylation in subsequent cell suspension culture coupled with high levels of MET1 and down-regulation of DNA demethylase ROS1 would then allow for establishment and perpetuation of genic DNA hypermethylation and for the creation of epialleles.

Support for this reprogramming model comes from analysis of the backcross progeny of *met1* mutant plants [36], and subsequent generations [107], which mimic the loss and reestablishment of DNA methylation in cell culture. In backcross plants, methylation patterns are reestablished only sporadically and depend on RNA-dependent DNA and histone H3K9 methylation, directed by 24-nt siRNA. The 21-nt sRNA were found in met1 mutants from the “TSI” repeat [107], which is a truncated form of *Athila* elements, as well as in a broader genome resource sequence of *met1* siRNA [71], although the origin of these siRNA and their persistence in backcrosses was not examined in these studies. Redirection of TE transcripts into a posttranscriptional silencing 21-nt pathway, rather than the transcriptional silencing 24-nt pathway, would account for the failure to reprogram TE DNA methylation in cultured cells. Thus reprogramming of DNA methylation plays a significant role in both animal and plant cell culture, although the role of sRNA in mammalian cell cultures has not yet been examined.

## Materials and Methods

### Plant material.

*Arabidopsis* cell line (ecotype Columbia) was maintained in Gamborg's B5 basal medium with minor organics (Sigma G5893) supplemented with 1.1 mg/l 2,4-D, 3 mM MES, and 3% sucrose. The cells were grown on a shaker at 160 rpm under consistent light condition at 23 °C and were subcultured every 7 d with a 1:10 (inoculum:fresh medium) dilution ratio.

The wild-type Col seedlings were grown on MS agar plates under standard conditions (day/light) for 2 wk. Callus samples were obtained through standard plant tissue culture techniques. Cotyledons of Arabidopsis thaliana, ecotype Columbia, were cut out 7 d after plating on MS plates and transferred onto 1× MS plates supplemented with auxin and cytokinin (0.15 μg/ml IAA and 0.625 μg/ml isopentanyladenine) and 3% (w/v) sucrose. Five weeks later, callus samples 5–8 mm in diameter were collected for gene expression analysis.

### Custom chromosome tiling microarray.

We used a custom printed genome tiling microarray that comprises approximately 18.6 Mb of Arabidopsis thaliana chromosome 4 sequence (∼14% of the entire *Arabidopsis* genome). Approximately 22,000 tiles, on average 1 kb in size, cover the majority of chromosome 4, including the entire heterochromatic knob on the short arm of chromosome 4 and several megabases of pericentromeric heterochromatin. Details of array design and production are described in [51].

### DNA methylation analysis.

Genomic DNA was isolated from cell suspension culture using Qiagen Plant DNeasy Mini Kit following the manufacturer's protocol (except that genomic DNA was eluted with 10 mM TrisCl [pH 8.0]). Ten micrograms of DNA was sheared to 1–3 kb in size, and half of the sheared DNA was subjected to digestion using McrBC restriction endonuclease, a restriction endonuclease that recognizes 5mC in a variety of sequence contexts (5′ (G/A)^m^C(N)_40–3000_(G/A)^m^C 3′) as described in [51]. Following digestion, both the treated and the untreated DNA sample were briefly run on a 1% agarose gel, and the DNA fragments 0.8–3 kb in size were isolated from the gel slices of both the digested and the undigested DNA sample using QiaexII gel extraction kit. Resulting DNA was amplified using a PCR-based approach described in [58]. Three micrograms of each amplified DNA sample was used in a labeling reaction using the Bioprime Array CGH kit and Cy3 and Cy5 fluorescent dyes. Each labeled DNA sample was eluted in 28 μl of 10 mM TrisCl (pH 8.0), and paired DNA samples were combined in a final volume of 70 μl of 4× SSC and 0.2% SDS. Each microarray hybridization was carried out at 63 °C overnight (14–16 h). Following hybridization, microarrays were washed for 8 min in 1× SSC, 0.2% SDS (warmed up to 50 °C), then for 5 min in 1× SSC (at room temperature), and finally in two washes of 0.2× SSC (2 min each). Microarrays were quickly dried by compressed air and immediately scanned using an Axon 4000B microarray scanner.

DNA methylation levels were measured by cohybridizing differentially labeled McrBC-treated and untreated DNA on a single array. In this way, copy-number differences are not contributing to the difference in hybridization signals on each array. A dye-swap experimental design provided the basis for these chromosome 4 tiling array experiments. DNA methylation was profiled by comparing McrBC-treated samples with untreated samples for both cell culture samples. The experimental design consisted of four replicated dye swaps. Sixteen total arrays were hybridized. Each tile was tested for methylation changes using a simple linear model. Details of the model, hypotheses, statistical test, and multiple testing adjustment are provided in Text S1.

### Chromatin immunoprecipitation and array hybridizations.

An approximate volume of 5 ml of sedimented cells from cell suspension culture were cross-linked by treating with 1% paraformaldehyde for 15 min. After adding 2 M glycine (100 mM final concentration) and incubating for 5 min to stop the cross-linking, the cells were washed three times with 1× PBS by centrifuging at 1,500 rpm for 5 min. The fixed cells were snap-frozen in liquid nitrogen and stored at −80 °C. Chromatin isolation and immunoprecipitation with antibodies against specific histone modifications (anti-H3K2me2, anti-H3K9me2, and anti-H3K56ac) were done according to [108] with minor modifications (an extra wash at each washing step). Antibodies used were from Upstate/Millipore (anti-H3K4me2: cat# 07–030 [lot# 26335], anti-H3Kme2: cat# 07–212 [lot# 27563], and anti-H3K56ac: cat# 07–677). Immunoprecipitated DNA was amplified, labeled, and hybridized to the custom genome tiling array using the procedures described above for genomic DNA amplification, fluorescent labeling, and microarray hybridization in DNA methylation analysis.

For each immunoprecipitation, we hybridized, amplified, and labeled immunoprecipitated DNA against input DNA using two biological and two technical replicates. As with the DNA methylation experiments, a dye swap provided the experimental design. A total of eight microarrays were hybridized for each immunoprecipitation. Details of the model, hypotheses, statistical test, and multiple testing adjustments are provided in Text S1.

### Gene expression microarray analysis.

Total RNA from 100 mg of 2-wk-old plate-grown seedlings (Col) and 100 mg of 4-d cell suspension culture was isolated using Qiagen Plant RNeasy Mini Kit. RNA quality was assessed on an Agilent 2100 Bioanalyzer, RNA 6000 Pico Series II Chips (Agilent). Samples were assessed by an RNA integrity number (RIN) score, and those with an RIN score of 7.5 or greater were included. The RNA quantity was assessed by Nanodrop ND-1000 (Nanodrop Technologies). Total RNA was amplified by a modified Eberwine Technique, using a Message Amp II kit (Ambion) for one round of amplification (1). The antisense RNA (aRNA) smear analysis for 3' bias was performed on select samples using Agilent 2100 Bioanalyzer RNA 6000 Nano Series II Chips (Agilent). Samples were then prepared for hybridization, hybridized, washed, and then scanned according to the manufacturer's instructions on ATH1 GeneChips (Affymetrix). Affymetrix QC metrics were used for quality control of the image data.

### Small RNA sequencing.

The sRNA fractions were isolated from the total RNA sample by size selection on denaturing 15% PAGE gels. RNA molecules between 19 nt and 28 nt in size were eluted from crushed gel slices in four gel volumes of 20 mM Tris (pH 8), 1 mM EDTA, 0.4 M NH_4_OAc, 0.5% SDS overnight, with shaking. sRNA was precipitated with glycoblue (Invitrogen) and three volumes of ethanol (−20 °C overnight) followed by ligation of the adenylated 3′ adapter (AMP-5′p = 5′-pCTGTAGGCACCATCAATdideoxyC-3′) and then the 5′ adapter (rArCrArCrUrCrUrUrUrCrCrCrUrArCrArCrGrArCrGrCrUrCrUrUrCrCrGrArUrC) as described in [109]. After size selection using 15% PAGE gel electrophoresis, sRNA libraries were reverser transcribed and PCR amplified using the forward PCR primer (5′-AATGATACGGCGACCACCGAACACTCTTTCCCT ACACGACG-3′) and the reverse PCR primer (5′-CAAGCAGAAGACGGCATACGATTGATGGTGCCTACAG-3′) for 22 cycles using proofreading Taq polymerase. The sRNA libraries were sequenced on an Illumina 1G sequencer using 48 cycles to allow for adequate 3′ adapted masking for precise size determination of the sRNA clones.

### RT-PCR.

Total RNA was isolated from young rosette leaves of 2-wk-old Columbia plants grown on plates under standard conditions, 4-d cell suspension culture, as well as from cotyledon-derived callus samples described above using Qiagen Plant RNeasy Mini Kit. Five micrograms of total RNA was treated with DNaseI and then reverse transcribed with SuperScript III with oligo dT primer in a 20-μl reaction. Two microliters of 5-fold diluted cDNA reaction was used as a template in the PCR reaction (for a final amount of 100 ng of starting total RNA per each PCR reaction). Thirty cycles of PCR were performed. The primer sequences used in RT-PCR are listed in Text S1.

### Computational analyses.

*General bioinformatics methods.* Histone modification, DNA methylation profiles, and sRNA sequencing data were loaded into a MySQL relational database implementing the Bio::DG::GFF schema, thus facilitating intersecting positional, quantitative, and class-based queries and computations. In addition, array data and genome annotations were displayed visually using a Generic Genome Browser, available for public examination at http://chromatin.cshl.edu/epiculture/.

Genes and noncoding RNAs were annotated based on the TAIR version 6 genome release. TEs and known non-TE classes such as the 180-bp centromeric repeat were annotated based on CENSOR analysis [110] of the *Arabidopsis* genome sequence using version 11.10 of the Repbase database [111]. Anonymous tandem repeats were identified using TandemRepeatsFinder [112], whereas miRNA precursors were localized in the genome by BLAST comparison of precursors sequences provided by Rfam [113] to the *Arabidopsis* genome. Other regions are assumed to be “Intergenic.”

### Analysis of small RNA sequences.

Adapter sequences were identified and removed using the cross_match algorithm (http://www.phrap.org/phredphrapconsed.html) using a minimum match size of 5 and a minimum score of 14. The nonredundant complement of sequences for the cell culture and inflorescence sRNA libraries was determined by a Perl script that indexed unique masked nucleotide sequences and the frequency with which they were identified. These sequences were aligned to the *Arabidopsis* genome sequence using BLATN [114] with a word size of 8, and further computational analysis was restricted to sRNA that perfectly matched the genome.

In order to examine sRNA distribution across the genome in an unbiased fashion, copy number-corrected sRNA frequencies were calculated based on BLATN-determined sequence identities for nonoverlapping 100-bp windows. These data were converted to UCSC Wiggle format and displayed in graphical form on our genome browser. Copy number correction was used to partition sequencing frequencies for each sRNA across all instances of that sRNA in the genome. It was performed by calculating the number of discrete, perfect matches for each sRNA from a BLAT query against the *Arabidopsis* genome as an estimate of copy number, then dividing the sequencing frequencies of individual sRNA matches by that value.

sRNA size class analysis was performed by parsing results of a genome-wide BLAT analysis to index sRNA matches by sRNA length and the annotation units within which they fell. To facilitate comparison between libraries, results were plotted in Microsoft Excel as percentages of sRNA matches in each size class relative to all matches from all size classes. Results were summarized for the genome as a whole and then partitioned to examine matches to particular transposable elements and classes of repeats.

The physical distribution for each sequence library of sRNAs within representative full-length TEs was determined by performing a BLATN of all sequences in the library against reference sequences. The number of sRNAs on each strand at each base position for the template sequences was computed based on sequencing frequency and the BLATN results and plotted as a histogram using Gnuplot 4.0.

Base preference analysis was performed for all sRNA matching the genome in each sequenced library by counting the number of sRNA sequences with a given nucleotide at each position, then dividing values for each nucleotide by the total number of sRNA sequences. Values from the first base were extracted and used for analyses in this paper. These analyses were performed on all sRNA at large, as well as upon a subset of sRNA in which known miRNAs were excluded, since many miRNAs are known to have specific first-base properties [59].

sRNA matches to miRNAs and their precursors were identified by matching the nonredundant complement of sRNA sequences to FASTA files provided by Rfam [113] using the Patscan algorithm [115] allowing up to two internal mismatches.

sRNA sequences have been deposited in GenBank and the accession numbers can be found at http://chromatin.cshl.edu/epiculture/.

### Analysis of ATH1 expression arrays.

Affymetrix .CEL files were imported into Agilent GeneSpring GX using the gcRMA algorithm for preprocessing. Significant differences among tissue sources were established using one-way analysis of variance (ANOVA) followed by control of the FDR to 5% using the method of Benjamini and Hochberg [116]. Inter-sample comparisons were extracted from the list of statistically significantly different genes using GeneSpring's built-in filtering tools. The GEO accession numbers for the ATH1 microarray data compiled in Table S5 are available at http://chromatin.cshl.edu/epiculture/.

## Supporting Information

Figure S1 *Arabidopsis* Cell Suspension Culture Characteristics of *Arabidopsis* cell line used in this study. Analytical FACS profiles of (A) 16-h postculture split (cells7), (B) 96-h postculture split (cells4), and (C) 4-wk-old *Arabidopsis* leaf. *Arabidopsis* cells are visualized in (B) (bar indicates 20 μm). (D) BrdU incorporation at different time points inbetween subculture.
(60 KB PDF)

Figure S2 Transcriptional Reactivation of TEs and the Associated Chromatin Modifications RT-PCR results for 15 selected TEs from chromosome 4 (A–N) showing their expression levels in Col-0 leaf tissue, cell suspension culture, and callus. Chromatin maps of the chromosomal regions corresponding to each TE tested (A–N) are shown, with the TEs annotated in green or pink, DNA methylation profiles of cell suspension culture and seedling shown in red, and the H3K9me2 and H3K4me2 profiles shown in purple.
(1.13 MB PDF)

Figure S3 Spatial Distribution and Frequency of 21-nt and 24-nt sRNA Sequences Specific to Various Families of TEs in the Leaf and Cell Suspension Culture (Cells7) Libraries A representative full-length *Helitron*, *AtGP1*, *AtCopia*, *AtVandal*, *AtMu13*, or *AtENSPM* TE was used as a sequence-matching template to map 21-nt and 24-nt sRNAs with perfect matches, resulting in single-nucleotide coverage of the template sequences. The coverage was plotted on the *y*-axis for both DNA strands (top strand in red and the bottom strand in green). The *x*-axis represents the template sequence in the 5′ to 3′ direction.
(375 KB PDF)

Figure S4 First Nucleotide Preference of sRNAs from Leaf, Cell Suspension Culture, and Callus Libraries The relative frequency of each of the four nucleotides at the 5′ end of each 21-, 22-, 23-, and 24-nt sRNA from the leaf, callus, and cells4 and cells7 sRNA libraries is plotted as a histogram (with known miRNA removed from the analysis). The 5′ nucleotide relative frequencies were also calculated for all sRNAs matching specific TE families (*Atlantys*, *Athila*, *AtGP*, *AtMu*, *AtENSPM*, *AtMu*, and *AtVandal*) from all four sRNA libraries.
(275 KB PDF)

Figure S5 Gene Expression Differences between Cell Suspension Culture, Seedling, and Callus Samples RT-PCR validation of gene expression differences between cell suspension culture, seedling, and callus samples for genes with roles in chromatin regulation. All PCR reactions were done at 32 cycles using 100 ng of total RNA per each reverse transcription reaction, and one-quarter of each PCR reaction was loaded on the gel and visualized using ethidium bromide (EtBr) staining and UV illumination.
(57 KB PDF)

Table S1 The Total Number of 21–24-nt *Arabidopsis* sRNA Reads Sequenced from Each Library and the Percentage Corresponding to Various Families of Transposable Elements (101 KB DOC)

Table S2 List of Genes with Significant DNA Methylation Present The list shows genes that either gain or lose DNA methylation in cell suspension culture relative to seedling in addition to genes that are methylated in both. The list contains two categories of genes, those with repeats associated with either the coding region or flanking the gene, and those without any repeat annotation associated with the particular gene coding region. Since this analysis was done on a per-tile basis, this list represents a compilation of *Arabidopsis* Genome Initiative (AGI) gene identifiers that associated with tiles on the chromosome 4 array.
(1.76 MB DOC)

Table S3 *Arabidopsis* Chromosome 4 Genes That Become Hypermethylated in Cell Suspension Culture with Concomitant Appearance of H3K9me2 and sRNAs in Cell Suspension Culture This list was created by filtering the epigenomic maps of cell suspension culture and seedling for genes that gain DNA and H3K9me2 methylation in cell suspension culture accompanied by a gain or an increase in sRNAs matching the same regions.
(51 KB DOC)

Table S4 List of ChromDB Genes with Significant Differences in Expression between Cell Suspension Culture and Seedling ATH1 microarray probes detecting significant differences in transcript levels between cell suspension culture and seedling were determined using one-way ANOVA (GeneSpring GX) followed by a multiple testing correction to an FDR of 0.05. These probes were cross-indexed to probes matching ChromDB chromatin regulators using a script that imported the expression levels with textual annotation supplied by ChromDB.
(57 KB XLS)

Table S5 Normalized Gene Expression Values in Seedling, Cell Suspension Culture, and Callus Samples for All the Probes Located on ATH1 Arrays (2.85 MB XLS)

Text S1 Supplemental Methods Supplemental text describing the statistical analyses of McrBC and ChIP-chip microarray experiments and PCR primer sequences used in RT-PCR.
(91 KB PDF)

## Abbreviations

ChIP: chromatin immunoprecipitation
H3K4me3: histone H3 trimethylation at lysine4
H3K56ac: histone H3 acetylated at lysine K56
miRNA: microRNA
nt: nucleotide
siRNA: small interfering RNA
RT-PCR: reverse-transcription PCR
SINE: short interspersed nuclear element
sRNA: small RNA
TE: transposable element
