# Supplementary material for: Epigenomic Consequences of Immortalized Plant Cell Suspension Culture
Source: PLoS Biol. 2008 Dec 9;6(12):e302. doi: 10.1371/journal.pbio.0060302 (PMC2596858; doi:10.1371/journal.pbio.0060302)
Supplement: Supplementary file 1 [file pbio.0060302.sd001.pdf]

Supplemental Methods (Tanurdzic et al.)

Methylation analysis. Arabidopsis chromosome 4 tiling microarray is employed to profile DNA methylation (i.e., comparing McrBC treated versus untreated) for cell culture samples collected at day 4 (Cells4 sample) and day 7 (Cells7 sample), respectively. The experimental design consisted of four replicated dye-swaps. Each tile is tested for methylation changes using a simple linear analysis of variance (ANOVA), $Y_{ijkl} = \mu + S_k + T_l + ST_{kl} + A_{i(k)} + D_j + \varepsilon_{ijkl}$, that partitions the sources of variation. The $\log_2$-tranformed, background corrected methylation level is denoted as Y_{ijkl} where biological sample k (k = 1 for Cells4 sample and 2 for Cells7 sample), under treatment condition l (l=1 for untreated, 2 for McrBC treated), on array i with dye j (j=1 for red dye and 2 for green dye). The overall tile specific mean effect is denoted as μ, and S, T, and D represent the main effects for sample, treatment, and dye, respectively. Interactions between sample and treatment are denoted as ST, and the random array effect A is nested within the sample. Furthermore, the random error ε_{ijkl} is assumed to be normally distributed with mean zero and constant variance. Three one-sided hypotheses are tested to identify tiles with higher levels of methylation in the untreated sample when compared to the McrBC treated samples. Specifically, when comparing total methylation differences, the two cell culture samples are combined, and the hypotheses, H_0: $T_1 = T_2$ vs. H_a: $T_1 > T_2$ are employed. However, for testing methylation in only the Cells4 sample, H_0: $T_1 + ST_{11} = T_2 + ST_{12}$ vs. H_a: $T_1 + ST_{11} > T_2 + ST_{12}$ are employed for each tile. Finally, for the Cells7 sample the hypotheses H_0: $T_1 + ST_{21} = T_2 + ST_{22}$ vs. H_a: $T_1 + ST_{21} > T_2 + ST_{22}$ tests the methylation difference for each tile. For all three tests, the methylation difference for each tile is adjusted against the median value of 576 control tiles taken from the same array. Statistically significant differences are determined at 5% false discovery rate (FDR) level using Benjamini and Hochberg's FDR controlling procedure.

ChIP-Chip analysis. Three sets of dye-swap experiments are used to identify significant enrichment for each of three antibodies (i.e., H3K56acetylation, H3K4me2 and H3K9me2) when compared with the whole cell extract DNA (WCE), ie input DNA. For

each experiment, a linear model $Y_{ijkm} = \mu + A_i + D_j + T_k + G_m + AG_{im} + DG_{jm} + TG_{km} + \varepsilon_{ijkm}$ is employed to partition the sources of variation. The $\log_2$-tranformed, background corrected data Y_{ijkmr}, represent the signals for tile m on array i with dye j under treatment k (k=1 for antibody and 2 for WCE). The overall mean effect is μ, and A, D, T, and G represent the array, dye, treatment, and tile main effects, respectively. The array by tile, dye by tile, and treatment by tile interactions are AG, DG, TG, respectively. The random error ε_{ijkm} is assumed to be normally distributed, with mean zero and constant variance. Once the sources of technical variation (e.g., global and tile-specific array and dye effects) and experimental variation (e.g., treatment and treatment by tile interaction) are estimated, differential enrichment is tested using hypotheses, $H_0: T_1 + TG_{1m} = T_2 + TG_{2m}$ vs. $H_a: T_1 + TG_{1m} > T_2 + TG_{2m}$, that acknowledge both the average treatment effect and the treatment by tile interaction (Craig et al. 2003). To address the multiple testing issues Benjamini and Hochberg's false discovery rate (FDR) controlling procedure is used to control the FDR at the 5% level.

Supplemental Methods

Table 1

List of PCR primers used in RT PCR experiments

Annotation	Forward Primer Sequence	Reverse Primer Sequence
At4g08375_Copia55_I	TGTCCTTACACGCCTCAGCA	TCCCAAGTTCTGCAGGTGAA
At4g08680_AtMu13	TCCCACAATGCCAAAGAGGT	TGCTGGACAAACCACAACCA
At4g04380_Copia32_I	GAGGGCAAAGAAGGGAGTCCT	GCCGGTTCTATTGCATCACC
At4g04390_Vandal9	GCAGGCGGATGTTCTCACTC	ACGGAGGACATGCCTGCTAA
At4g04393_Vandal7	GCGTCTGCATCGAACACAAG	GCGGCACCACATAAATCGTT
At4g03790_Athila2_I	GGGACATGCGGAATCTCTTG	CTTCCACCGCTACAGGTTCG
AT4G22450_Harbinger	CGTGTTTGGTTTTGGTTGTCA	TGCGCATCATTTTCATCACC
AT4G05505_AtSPM9	GCCCGTGAGAATGATGAAGG	ATGCCTCTGCCTCACGATGT
At4g06588_AtSPM11	GCGATGCCTTTTTGTGGAGA	GACCTAAGGGGACATGGTGGA
AT4G04010_Arnold2	CGGTTGTAAACCATGCATACCC	CACTGGGAACCGTTCAACAA

atlantys_rt	CATCCTGATTGGCTTGGACT	AATGCCGCTACGGAGTAAGA
tc12f11_Brodyaga2	TCCGTAAACACCACAATCAAA	CATCCGGTTTGACCAAAACTA
AT4G07338_Helitron4	CTGCCATCCTTTTCCCGTTT	CAGCCAGTGGTTGTCCTCAA
AT4G15590_LINE1_2	TAGGCTTCCCCAATCCTGCT	CACGGATGCGATTGGAATTT
AT4G01490_LINE1_6	TCTTCAAGGATCGCCTCAGC	TGCTTGGAATCGGACGCTAT

References.

B.A. Craig, M.A. Black, and R.W. Doerge. 2003. Gene Expression Data: The technology and statistical analysis. Journal of Agricultural, Biological, and Environmental Statistics (JABES) 8(1):1-28.
