# Supplementary material for: Epigenomic Consequences of Immortalized Plant Cell Suspension Culture
Source: PLoS Biol. 2008 Dec 9;6(12):e302. doi: 10.1371/journal.pbio.0060302 (PMC2596858; doi:10.1371/journal.pbio.0060302)
Supplement: Supplementary file 3 [file pbio.0060302.sg002.pdf]

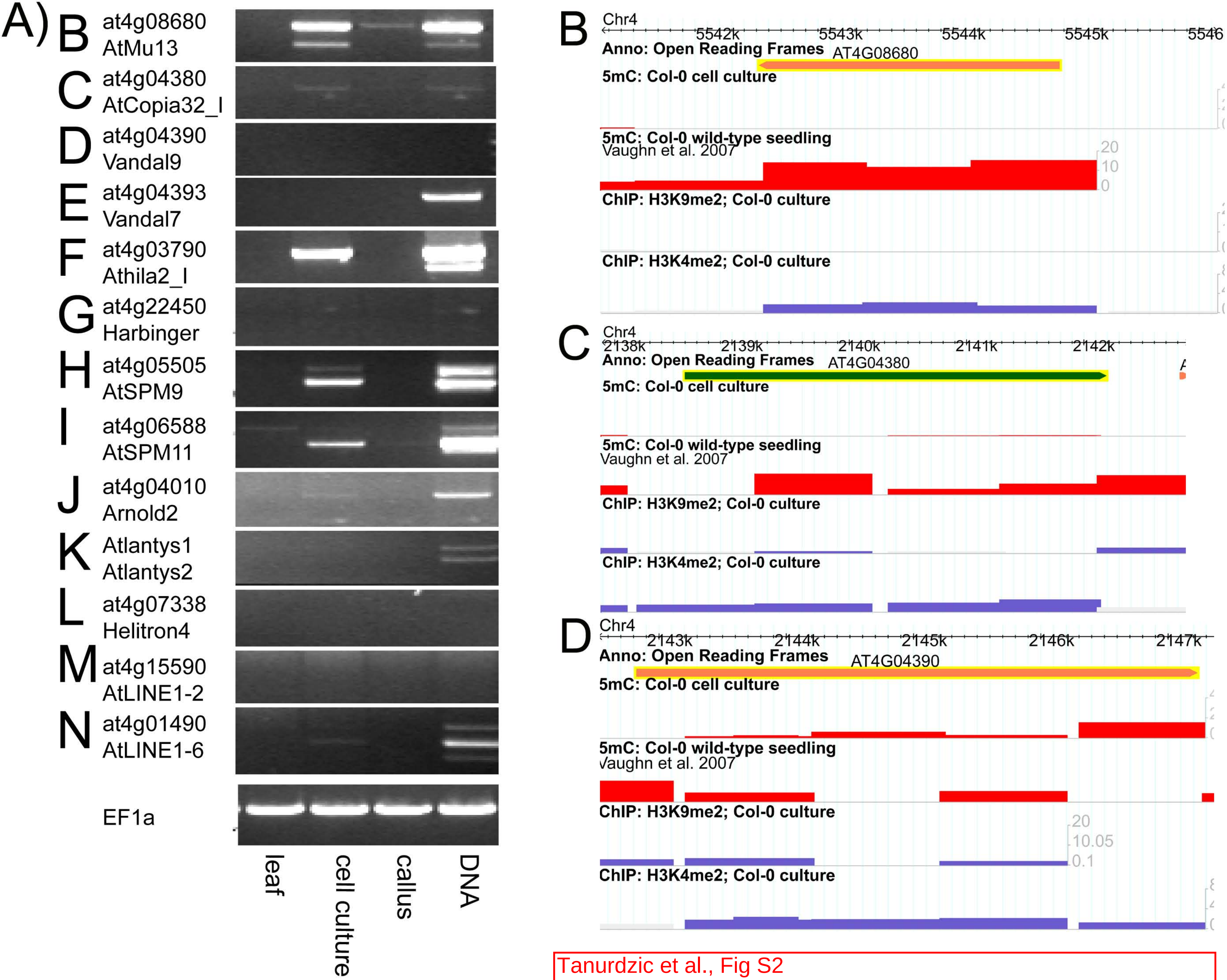

Tanurdzic et al., Fig S2

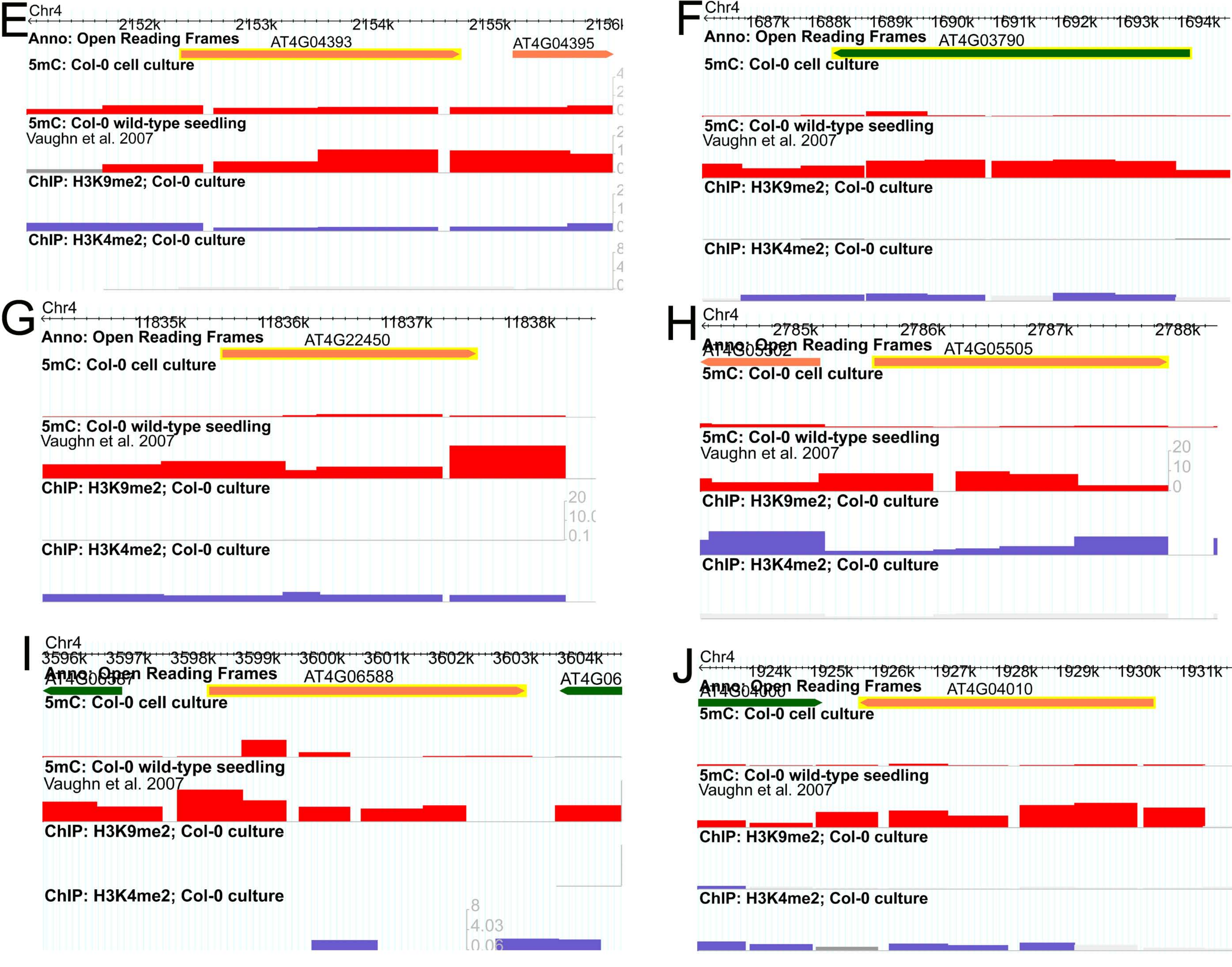

E
Chr4
2152k
2153k
2154k
2155k
Anno: Open Reading Frames
AT4G04393
AT4G04395
5mC: Col-0 cell culture
5mC: Col-0 wild-type seedling
Vaughn et al. 2007
ChIP: H3K9me2; Col-0 culture
ChIP: H3K4me2; Col-0 culture
F
Chr4
1687k
1688k
1689k
1690k
1691k
1692k
1693k
1694k
Anno: Open Reading Frames
AT4G03790
5mC: Col-0 cell culture
5mC: Col-0 wild-type seedling
Vaughn et al. 2007
ChIP: H3K9me2; Col-0 culture
ChIP: H3K4me2; Col-0 culture
G
Chr4
11835k
11836k
11837k
11838k
Anno: Open Reading Frames
AT4G22450
5mC: Col-0 cell culture
5mC: Col-0 wild-type seedling
Vaughn et al. 2007
ChIP: H3K9me2; Col-0 culture
20
ChIP: H3K4me2; Col-0 culture
H
Chr4
2785k
2786k
2787k
2788k
Anno: Open Reading Frames
AT4G05505
5mC: Col-0 cell culture
5mC: Col-0 wild-type seedling
Vaughn et al. 2007
20
10
0
ChIP: H3K9me2; Col-0 culture
ChIP: H3K4me2; Col-0 culture
I
Chr4
3596k
3597k
3598k
3599k
3600k
3601k
3602k
3603k
3604k
Anno: Open Reading Frames
AT4G06588
5mC: Col-0 cell culture
5mC: Col-0 wild-type seedling
Vaughn et al. 2007
ChIP: H3K9me2; Col-0 culture
ChIP: H3K4me2; Col-0 culture
8
4.03
J
Chr4
1924k
1925k
1926k
1927k
1928k
1929k
1930k
1931k
Anno: Open Reading Frames
AT4G04010
5mC: Col-0 cell culture
5mC: Col-0 wild-type seedling
Vaughn et al. 2007
ChIP: H3K9me2; Col-0 culture
ChIP: H3K4me2; Col-0 culture

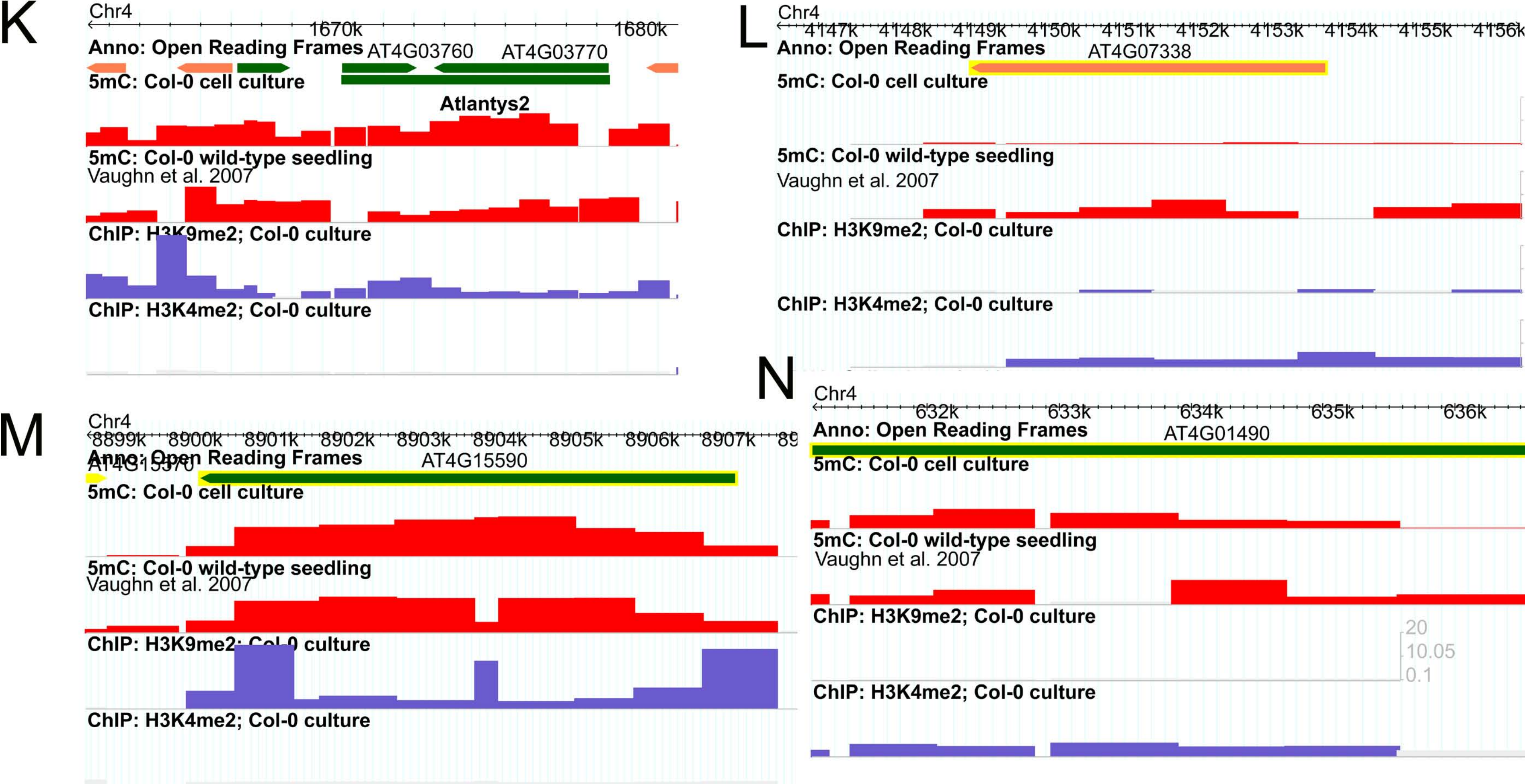

K
Chr4
1670k
1680k
Anno: Open Reading Frames
AT4G03760
AT4G03770
5mC: Col-0 cell culture
Atlantys2
5mC: Col-0 wild-type seedling
Vaughn et al. 2007
ChIP: H3K9me2; Col-0 culture
ChIP: H3K4me2; Col-0 culture
L
Chr4
4147k 4148k 4149k 4150k 4151k 4152k 4153k 4154k 4155k 4156k
Anno: Open Reading Frames
AT4G07338
5mC: Col-0 cell culture
5mC: Col-0 wild-type seedling
Vaughn et al. 2007
ChIP: H3K9me2; Col-0 culture
ChIP: H3K4me2; Col-0 culture
M
Chr4
8899k 8900k 8901k 8902k 8903k 8904k 8905k 8906k 8907k
Anno: Open Reading Frames
AT4G15570
AT4G15590
5mC: Col-0 cell culture
5mC: Col-0 wild-type seedling
Vaughn et al. 2007
ChIP: H3K9me2; Col-0 culture
ChIP: H3K4me2; Col-0 culture
N
Chr4
632k 633k 634k 635k 636k
Anno: Open Reading Frames
AT4G01490
5mC: Col-0 cell culture
5mC: Col-0 wild-type seedling
Vaughn et al. 2007
ChIP: H3K9me2; Col-0 culture
20
10.05
0.1
ChIP: H3K4me2; Col-0 culture
