# Supplementary material for: Epigenomic Consequences of Immortalized Plant Cell Suspension Culture
Source: PLoS Biol. 2008 Dec 9;6(12):e302. doi: 10.1371/journal.pbio.0060302 (PMC2596858; doi:10.1371/journal.pbio.0060302)
Supplement: Supplementary file 4 [file pbio.0060302.sg003.pdf]

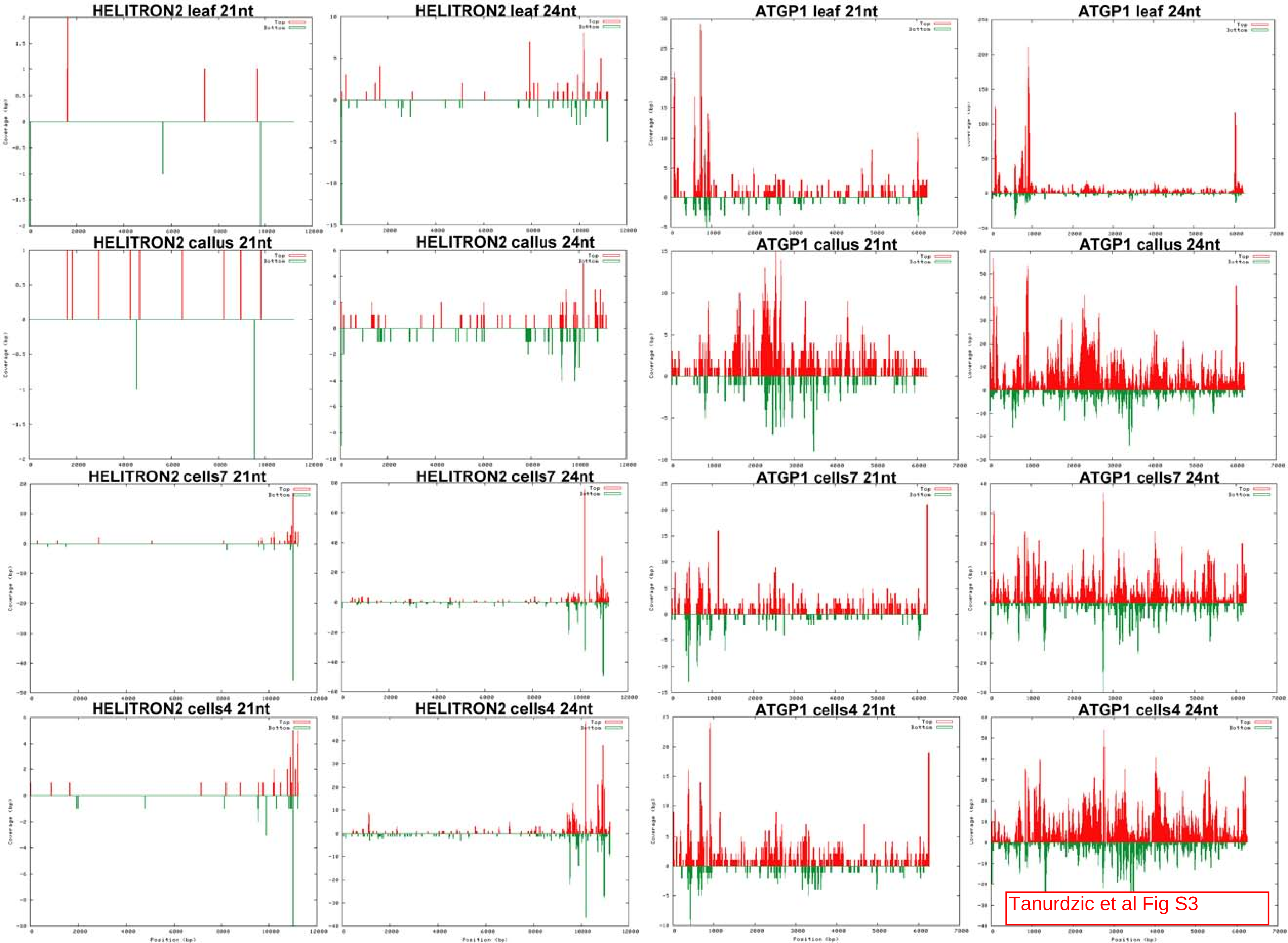
HELITRON2 leaf 21nt
HELITRON2 leaf 24nt
ATGP1 leaf 21nt
ATGP1 leaf 24nt
HELITRON2 callus 21nt
HELITRON2 callus 24nt
ATGP1 callus 21nt
ATGP1 callus 24nt
HELITRON2 cells7 21nt
HELITRON2 cells7 24nt
ATGP1 cells7 21nt
ATGP1 cells7 24nt
HELITRON2 cells4 21nt
HELITRON2 cells4 24nt
ATGP1 cells4 21nt
ATGP1 cells4 24nt
Top
Bottom
Coverage (bp)
Position (bp)
Tanurdzic et al Fig S3

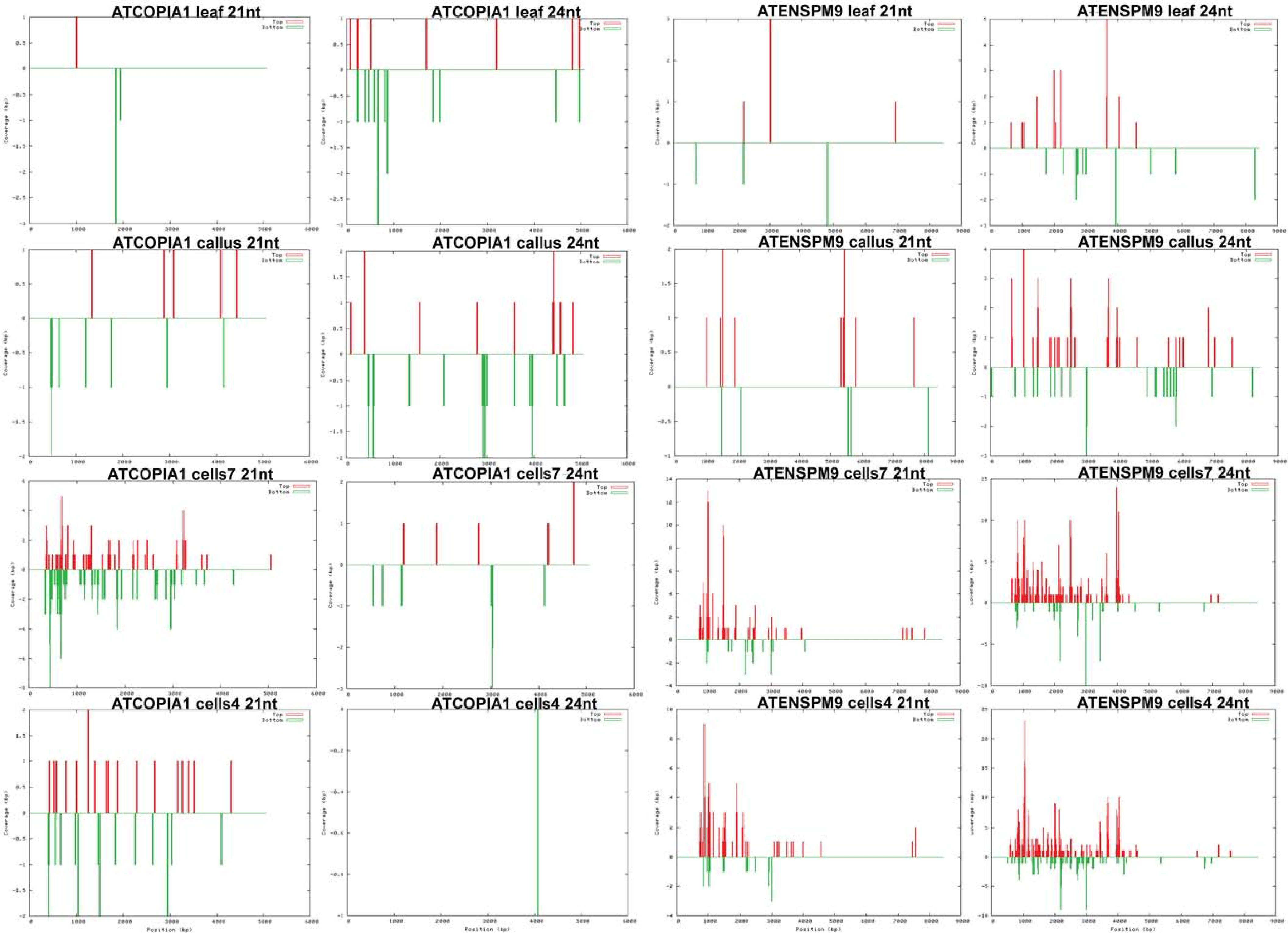
ATCOPIA1 leaf 21nt
ATCOPIA1 leaf 24nt
ATENSPM9 leaf 21nt
ATENSPM9 leaf 24nt
ATCOPIA1 callus 21nt
ATCOPIA1 callus 24nt
ATENSPM9 callus 21nt
ATENSPM9 callus 24nt
ATCOPIA1 cells7 21nt
ATCOPIA1 cells7 24nt
ATENSPM9 cells7 21nt
ATENSPM9 cells7 24nt
ATCOPIA1 cells4 21nt
ATCOPIA1 cells4 24nt
ATENSPM9 cells4 21nt
ATENSPM9 cells4 24nt
Top
Bottom
Coverage (bp)
Position (bp)

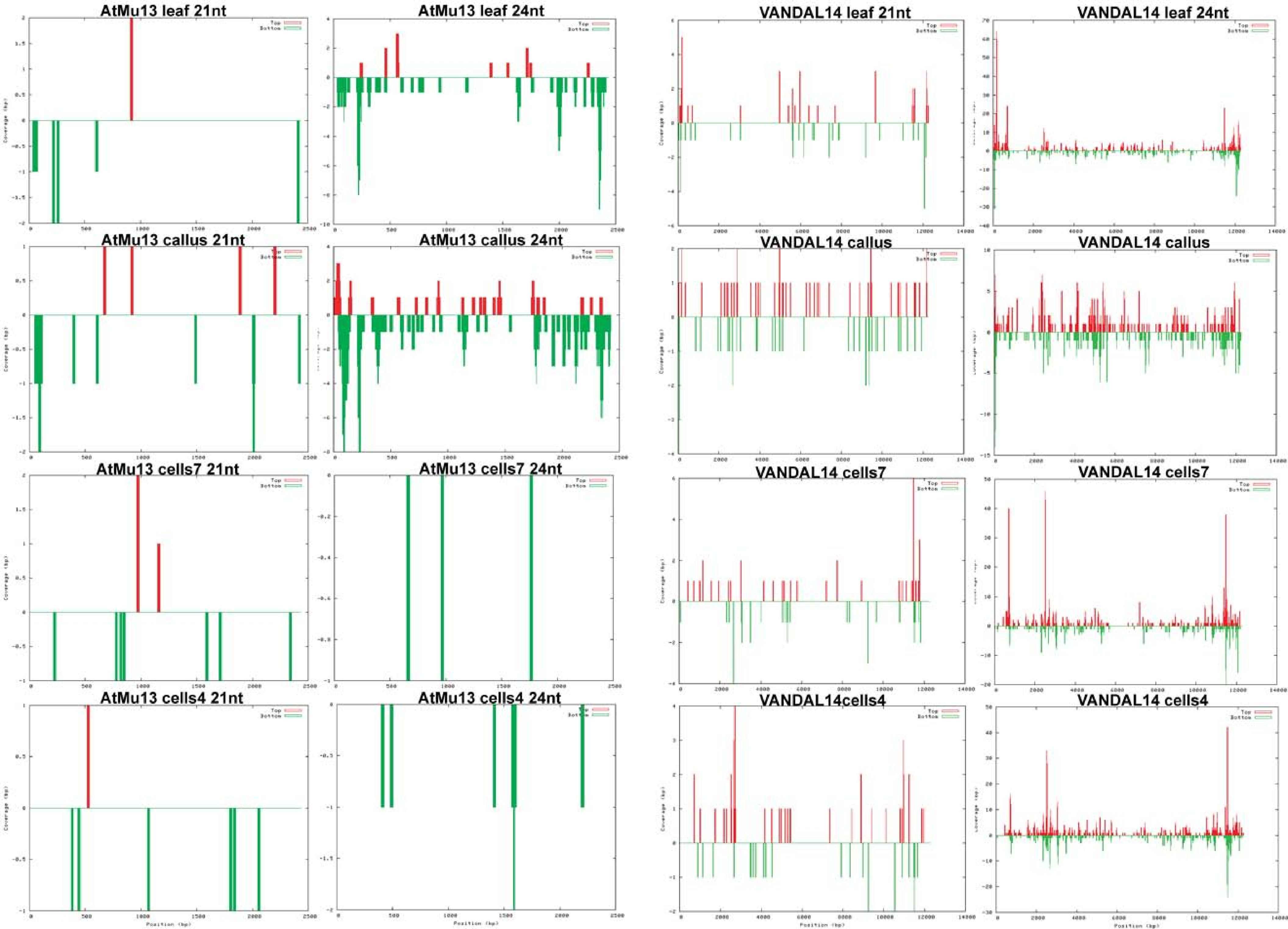
AtMu13 leaf 21nt
AtMu13 leaf 24nt
VANDAL14 leaf 21nt
VANDAL14 leaf 24nt
AtMu13 callus 21nt
AtMu13 callus 24nt
VANDAL14 callus
VANDAL14 callus
AtMu13 cells7 21nt
AtMu13 cells7 24nt
VANDAL14 cells7
VANDAL14 cells7
AtMu13 cells4 21nt
AtMu13 cells4 24nt
VANDAL14cells4
VANDAL14 cells4
Top
Bottom
Coverage (bp)
Position (bp)
