# Supplementary material for: Epigenomic Consequences of Immortalized Plant Cell Suspension Culture
Source: PLoS Biol. 2008 Dec 9;6(12):e302. doi: 10.1371/journal.pbio.0060302 (PMC2596858; doi:10.1371/journal.pbio.0060302)
Supplement: Supplementary file 6 [file pbio.0060302.sg005.pdf]

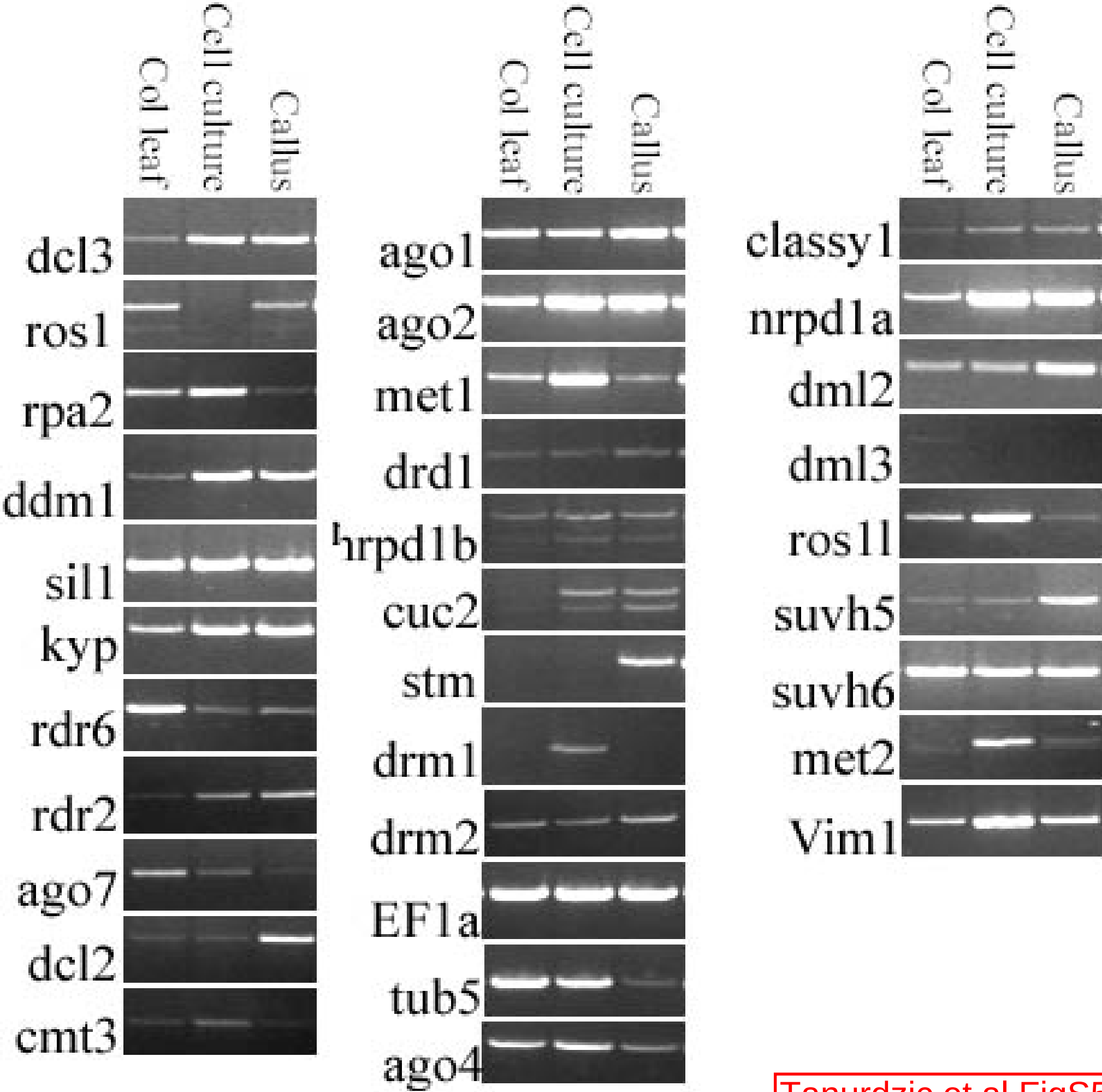

Tanurdzic et al FigS5
