# Supplementary material for: Epigenomic Consequences of Immortalized Plant Cell Suspension Culture
Source: PLoS Biol. 2008 Dec 9;6(12):e302. doi: 10.1371/journal.pbio.0060302 (PMC2596858; doi:10.1371/journal.pbio.0060302)
Supplement: Supplementary file 7 [file pbio.0060302.st001.doc]

| **Tanurdzic et al., Supplemental Table S1** | | | | | | |
| --- | --- | --- | --- | --- | --- | --- |
|  |  |  |  |  |  |  |
|  |  | 21nt | 22nt | 23nt | 24nt | All Sizes |
| Total sRNA | Leaf | 386175 | 56810 | 275646 | 202324 | 1737719 |
|  | Cells7 | 293962 | 76626 | 180664 | 410349 | 1910836 |
|  | Cells4 | 588096 | 200303 | 131098 | 330318 | 3075388 |
|  | Callus | 215064 | 134267 | 155936 | 324343 | 2070923 |
| ATHILA | Leaf | 0.39% | 1.63% | 1.31% | 3.13% |  |
|  | Cells7 | 21.79% | 17.13% | 10.06% | 16.44% |  |
|  | Cells4 | 32.35% | 19.68% | 4.96% | 8.52% |  |
|  | Callus | 2.55% | 4.16% | 2.98% | 5.79% |  |
| ATLANTYS | Leaf | 0.06% | 0.26% | 0.28% | 0.52% |  |
|  | Cells7 | 0.24% | 0.51% | 0.68% | 0.80% |  |
|  | Cells4 | 0.11% | 0.24% | 0.61% | 1.43% |  |
|  | Callus | 0.38% | 0.68% | 0.55% | 0.99% |  |
| AtHELITRON | Leaf | 0.29% | 0.68% | 1.70% | 3.78% |  |
|  | Cells7 | 1.54% | 1.15% | 1.06% | 1.76% |  |
|  | Cells4 | 1.48% | 0.81% | 0.65% | 1.41% |  |
|  | Callus | 0.31% | 0.44% | 1.14% | 1.68% |  |
| AtMU | Leaf | 0.06% | 0.16% | 0.37% | 0.70% |  |
|  | Cells7 | 0.18% | 0.22% | 0.23% | 0.27% |  |
|  | Cells4 | 0.09% | 0.07% | 0.10% | 0.25% |  |
|  | Callus | 0.08% | 0.11% | 0.31% | 0.46% |  |
| AtCOPIA | Leaf | 0.09% | 0.29% | 0.57% | 1.17% |  |
|  | Cells7 | 1.26% | 1.07% | 1.09% | 1.55% |  |
|  | Cells4 | 0.95% | 0.58% | 0.66% | 1.45% |  |
|  | Callus | 0.28% | 0.42% | 0.57% | 1.02% |  |
| AtENSPM | Leaf | 0.04% | 0.13% | 0.19% | 0.46% |  |
|  | Cells7 | 0.17% | 0.25% | 0.37% | 0.55% |  |
|  | Cells4 | 0.10% | 0.13% | 0.32% | 0.82% |  |
|  | Callus | 0.12% | 0.21% | 0.24% | 0.47% |  |
| AtVANDAL | Leaf | 0.59% | 1.47% | 2.78% | 4.37% |  |
|  | Cells7 | 1.41% | 1.33% | 2.03% | 2.99% |  |
|  | Cells4 | 1.40% | 1.16% | 8.53% | 3.91% |  |
|  | Callus | 0.69% | 1.09% | 1.85% | 2.67% |  |
