# Supplementary material for: Epigenomic Consequences of Immortalized Plant Cell Suspension Culture
Source: PLoS Biol. 2008 Dec 9;6(12):e302. doi: 10.1371/journal.pbio.0060302 (PMC2596858; doi:10.1371/journal.pbio.0060302)
Supplement: Supplementary file 8 [file pbio.0060302.st002.doc]

| **Tanurdzic et al., Supplemental Table S2** | | | | | |  |  |  |  |
| --- | --- | --- | --- | --- | --- | --- | --- | --- | --- |
|  | |  | |  | |  |  |  |  |
| **Seedling Only** | | **Culture Only** | | **Intersection** | |  |  |  |  |
| **Gene** | **Class** | **Gene** | **Class** | **Gene** | **Class** |  |  |  |  |
| At4g30470 | Gene | At4g09460 | Gene | At4g23040 | Gene |  |  |  |  |
| At4g14145 | Gene | At4g05270 | Gene | At4g33140 | Gene |  |  |  |  |
| At4g24690 | Gene | At4g20890 | Gene | At4g22390 | Gene |  |  |  |  |
| At4g27830 | Gene | At4g32175 | Gene | At4g12720 | Gene |  |  |  |  |
| At4g29660 | Gene | At4g16210 | Gene | At4g18640 | Gene |  |  |  |  |
| At4g26090 | Gene | At4g20110 | Gene | At4g26900 | Gene |  |  |  |  |
| At4g17615 | Gene | At4g35390 | Gene | At4g14570 | Gene |  |  |  |  |
| At4g22180 | Gene | At4g04614 | Gene | At4g08800 | Gene |  |  |  |  |
| At4g14050 | Gene | At4g34980 | Gene | At4g11010 | Gene |  |  |  |  |
| At4g35440 | Gene | At4g35280 | Gene | At4g17620 | Gene |  |  |  |  |
| At4g00170 | Gene | At4g25980 | Gene | At4g16650 | Gene |  |  |  |  |
| At4g24640 | Gene | At4g08039 | Gene | At4g25230 | Gene |  |  |  |  |
| At4g22050 | Gene | At4g16730 | Gene | At4g32720 | Gene |  |  |  |  |
| At4g24810 | Gene | At4g19760 | Gene | At4g17040 | Gene |  |  |  |  |
| At4g21990 | Gene | At4g12420 | Gene | At4g24820 | Gene |  |  |  |  |
| At4g00305 | Gene | At4g17710 | Gene | At4g39980 | Gene |  |  |  |  |
| At4g11370 | Gene | At4g15200 | Gene | At4g16130 | Gene |  |  |  |  |
| At4g12545 | Gene | At4g26555 | Gene | At4g20650 | Gene |  |  |  |  |
| At4g01930 | Gene | At4g04470 | Gene | At4g16340 | Gene |  |  |  |  |
| At4g38160 | Gene | At4g35690 | Gene | At4g11450 | Gene |  |  |  |  |
| At4g35020 | Gene | At4g33700 | Gene | At4g04680 | Gene |  |  |  |  |
| At4g31870 | Gene | At4g04960 | Gene | At4g38510 | Gene |  |  |  |  |
| At4g21585 | Gene | At4g18010 | Gene | At4g26640 | Gene |  |  |  |  |
| At4g37280 | Gene | At4g00234 | Gene | At4g08630 | Gene |  |  |  |  |
| At4g14980 | Gene | At4g27000 | Gene | At4g24840 | Gene |  |  |  |  |
| At4g00905 | Gene | At4g25650 | Gene | At4g39120 | Gene |  |  |  |  |
| At4g36640 | Gene | At4g30240 | Gene | At4g29180 | Gene |  |  |  |  |
| At4g28040 | Gene | At4g17570 | Gene | At4g35920 | Gene |  |  |  |  |
| At4g31600 | Gene | At4g23200 | Gene | At4g20770 | Gene |  |  |  |  |
| At4g23360 | Gene | At4g04810 | Gene | At4g14910 | Gene |  |  |  |  |
| At4g31875 | Gene | At4g29810 | Gene | At4g33210 | Gene |  |  |  |  |
| At4g33770 | Gene | At4g32695 | Gene | At4g27720 | Gene |  |  |  |  |
| At4g26670 | Gene | At4g31130 | Gene | At4g24790 | Gene |  |  |  |  |
| At4g37910 | Gene | At4g15640 | Gene | At4g15130 | Gene |  |  |  |  |
| At4g22380 | Gene | At4g39140 | Gene | At4g22970 | Gene |  |  |  |  |
| At4g21460 | Gene | At4g21480 | Gene | At4g16150 | Gene |  |  |  |  |
| At4g36780 | Gene | At4g15545 | Gene | At4g28570 | Gene |  |  |  |  |
| At4g12080 | Gene | At4g09570 | Gene | At4g11270 | Gene |  |  |  |  |
| At4g38530 | Gene | At4g14080 | Gene | At4g20560 | Gene |  |  |  |  |
| At4g10320 | Gene | At4g09530 | Gene | At4g37460 | Gene |  |  |  |  |
| At4g34420 | Gene | At4g04404 | Gene | At4g32920 | Gene |  |  |  |  |
| At4g35770 | Gene | At4g14190 | Gene | At4g14180 | Gene |  |  |  |  |
| At4g32500 | Gene | At4g31950 | Gene | At4g13820 | Gene |  |  |  |  |
| At4g31360 | Gene | At4g18480 | Gene | At4g35410 | Gene |  |  |  |  |
| At4g33370 | Gene | At4g12540 | Gene | At4g15240 | Gene |  |  |  |  |
| At4g10650 | Gene | At4g04630 | Gene | At4g15415 | Gene |  |  |  |  |
| At4g09310 | Gene | At4g26530 | Gene | At4g20700 | Gene |  |  |  |  |
| At4g23750 | Gene | At4g28230 | Gene | At4g34640 | Gene |  |  |  |  |
| At4g18290 | Gene | At4g02650 | Gene | At4g16360 | Gene |  |  |  |  |
| At4g12060 | Gene | At4g24180 | Gene | At4g03340 | Gene |  |  |  |  |
| At4g38830 | Gene | At4g18510 | Gene | At4g36050 | Gene |  |  |  |  |
| At4g23760 | Gene | At4g31970 | Gene | At4g29220 | Gene |  |  |  |  |
| At4g00360 | Gene | At4g33730 | Gene | At4g08700 | Gene |  |  |  |  |
| At4g10750 | Gene | At4g30340 | Gene | At4g19006 | Gene |  |  |  |  |
| At4g36880 | Gene | At4g38800 | Gene | At4g38360 | Gene |  |  |  |  |
| At4g37235 | Gene | At4g11880 | Gene | At4g38790 | Gene |  |  |  |  |
| At4g35850 | Gene | At4g33830 | Gene | At4g32670 | Gene |  |  |  |  |
| At4g14100 | Gene | At4g03280 | Gene | At4g39440 | Gene |  |  |  |  |
| At4g37210 | Gene | At4g05450 | Gene | At4g20530 | Gene |  |  |  |  |
| At4g29200 | Gene | At4g24010 | Gene | At4g27050 | Gene |  |  |  |  |
| At4g32990 | Gene | At4g33600 | Gene | At4g07740 | Gene |  |  |  |  |
| At4g27220 | Gene | At4g01910 | Gene | At4g35560 | Gene |  |  |  |  |
| At4g00220 | Gene | At4g20310 | Gene | At4g18530 | Gene |  |  |  |  |
| At4g26250 | Gene | At4g38930 | Gene | At4g00740 | Gene |  |  |  |  |
| At4g31530 | Gene | At4g02200 | Gene | At4g36550 | Gene |  |  |  |  |
| At4g30120 | Gene | At4g33860 | Gene | At4g17730 | Gene |  |  |  |  |
| At4g09110 | Gene | At4g13040 | Gene | At4g04950 | Gene |  |  |  |  |
| At4g34950 | Gene | At4g05110 | Gene | At4g34330 | Gene |  |  |  |  |
| At4g26380 | Gene | At4g27800 | Gene | At4g27910 | Gene |  |  |  |  |
| At4g30810 | Gene | At4g11030 | Gene | At4g07670 | Gene |  |  |  |  |
| At4g29860 | Gene | At4g14030 | Gene | At4g10250 | Gene |  |  |  |  |
| At4g29305 | Gene | At4g33625 | Gene | At4g22990 | Gene |  |  |  |  |
| At4g22610 | Gene | At4g17510 | Gene | At4g26300 | Gene |  |  |  |  |
| At4g36925 | Gene | At4g32470 | Gene | At4g03550 | Gene |  |  |  |  |
| At4g10630 | Gene | At4g02290 | Gene | At4g04910 | Gene |  |  |  |  |
| At4g24550 | Gene | At4g00150 | Gene | At4g24200 | Gene |  |  |  |  |
| At4g18910 | Gene | At4g27600 | Gene | At4g04030 | Gene |  |  |  |  |
| At4g09200 | Gene | At4g12960 | Gene | At4g36210 | Gene |  |  |  |  |
| At4g01460 | Gene | At4g09060 | Gene | At4g27370 | Gene |  |  |  |  |
| At4g40042 | Gene | At4g02195 | Gene | At4g27180 | Gene |  |  |  |  |
| At4g02690 | Gene | At4g31115 | Gene | At4g29210 | Gene |  |  |  |  |
| At4g14440 | Gene | At4g18900 | Gene | At4g08920 | Gene |  |  |  |  |
| At4g00320 | Gene | At4g10440 | Gene | At4g28300 | Gene |  |  |  |  |
| At4g31920 | Gene | At4g13580 | Gene | At4g21470 | Gene |  |  |  |  |
| At4g37980 | Gene | At4g19340 | Gene | At4g08874 | Gene |  |  |  |  |
| At4g39580 | Gene | At4g27300 | Gene | At4g30610 | Gene |  |  |  |  |
| At4g26310 | Gene | At4g25510 | Gene | At4g36080 | Gene |  |  |  |  |
| At4g34520 | Gene | At4g16230 | Gene | At4g32440 | Gene |  |  |  |  |
| At4g24890 | Gene | At4g04210 | Gene | At4g05080 | Gene |  |  |  |  |
| At4g33945 | Gene | At4g16370 | Gene | At4g03820 | Gene |  |  |  |  |
| At4g15870 | Gene | At4g29250 | Gene | At4g02610 | Gene |  |  |  |  |
| At4g18630 | Gene | At4g05490 | Gene | At4g22350 | Gene |  |  |  |  |
| At4g24400 | Gene | At4g25310 | Gene | At4g36960 | Gene |  |  |  |  |
| At4g01560 | Gene | At4g33350 | Gene | At4g30550 | Gene |  |  |  |  |
| At4g29850 | Gene | At4g21130 | Gene | At4g25870 | Gene |  |  |  |  |
| At4g21520 | Gene | At4g05140 | Gene | At4g18905 | Gene |  |  |  |  |
| At4g20960 | Gene | At4g00416 | Gene | At4g21100 | Gene |  |  |  |  |
| At4g35430 | Gene | At4g24860 | Gene | At4g10760 | Gene |  |  |  |  |
| At4g09320 | Gene | At4g34020 | Gene | At4g29040 | Gene |  |  |  |  |
| At4g39955 | Gene | At4g37140 | Gene | At4g20940 | Gene |  |  |  |  |
| At4g37330 | Gene | At4g33010 | Gene | At4g40000 | Gene |  |  |  |  |
| At4g01915 | Gene | At4g24720 | Gene | At4g33580 | Gene |  |  |  |  |
| At4g33320 | Gene | At4g34080 | Gene | At4g00440 | Gene |  |  |  |  |
| At4g35350 | Gene | At4g34260 | Gene | At4g10000 | Gene |  |  |  |  |
| At4g25820 | Gene | At4g13480 | Gene | At4g29500 | Gene |  |  |  |  |
| At4g01590 | Gene | At4g18220 | Gene | At4g33060 | Gene |  |  |  |  |
| At4g16765 | Gene | At4g29450 | Gene | At4g12810 | Gene |  |  |  |  |
| At4g25550 | Gene | At4g03460 | Gene | At4g22120 | Gene |  |  |  |  |
| At4g20860 | Gene | At4g06534 | Gene | At4g20570 | Gene |  |  |  |  |
| At4g00165 | Gene | At4g31500 | Gene | At4g38020 | Gene |  |  |  |  |
| At4g22710 | Gene | At4g21705 | Gene | At4g17280 | Gene |  |  |  |  |
| At4g22340 | Gene | At4g29490 | Gene | At4g29940 | Gene |  |  |  |  |
| At4g23520 | Gene | At4g00190 | Gene | At4g23640 | Gene |  |  |  |  |
| At4g31460 | Gene | At4g25720 | Gene | At4g06536 | Gene |  |  |  |  |
| At4g17540 | Gene | At4g14615 | Gene | At4g21400 | Gene |  |  |  |  |
| At4g34490 | Gene | At4g02550 | Gene | At4g02390 | Gene |  |  |  |  |
| At4g31780 | Gene | At4g03010 | Gene | At4g35740 | Gene |  |  |  |  |
| At4g09420 | Gene | At4g37490 | Gene | At4g20380 | Gene |  |  |  |  |
| At4g30996 | Gene | At4g03610 | Gene | At4g26650 | Gene |  |  |  |  |
| At4g32630 | Gene | At4g15480 | Gene | At4g35290 | Gene |  |  |  |  |
| At4g22720 | Gene | At4g30140 | Gene | At4g20060 | Gene |  |  |  |  |
| At4g03165 | Gene+Repeat | At4g32680 | Gene | At4g01940 | Gene |  |  |  |  |
| At4g08685 | Gene+Repeat | At4g31020 | Gene | At4g34390 | Gene |  |  |  |  |
| At4g01883 | Gene+Repeat | At4g20460 | Gene | At4g30090 | Gene |  |  |  |  |
| At4g22690 | Gene+Repeat | At4g04500 | Gene | At4g28370 | Gene |  |  |  |  |
| At4g35380 | Gene+Repeat | At4g25440 | Gene | At4g17110 | Gene |  |  |  |  |
| At4g20450 | Gene+Repeat | At4g29620 | Gene | At4g30790 | Gene |  |  |  |  |
| At4g39090 | Gene+Repeat | At4g10540 | Gene | At4g34450 | Gene |  |  |  |  |
| At4g39540 | Gene+Repeat | At4g16700 | Gene | At4g09830 | Gene |  |  |  |  |
| At4g15370 | Gene+Repeat | At4g05130 | Gene | At4g32660 | Gene |  |  |  |  |
| At4g12330 | Gene+Repeat | At4g28000 | Gene | At4g38110 | Gene |  |  |  |  |
| At4g03205 | Gene+Repeat | At4g37660 | Gene | At4g24740 | Gene |  |  |  |  |
| At4g27870 | Gene+Repeat | At4g00490 | Gene | At4g20400 | Gene |  |  |  |  |
| At4g04700 | Gene+Repeat | At4g18140 | Gene | At4g32450 | Gene |  |  |  |  |
| At4g02940 | Gene+Repeat | At4g12910 | Gene | At4g29680 | Gene |  |  |  |  |
| At4g28650 | Gene+Repeat | At4g20670 | Gene | At4g02060 | Gene |  |  |  |  |
| At4g27310 | Gene+Repeat | At4g31010 | Gene | At4g31210 | Gene |  |  |  |  |
| At4g21570 | Gene+Repeat | At4g23190 | Gene | At4g39530 | Gene |  |  |  |  |
| At4g26730 | Gene+Repeat | At4g19840 | Gene | At4g23660 | Gene |  |  |  |  |
| At4g34730 | Gene+Repeat | At4g18990 | Gene | At4g22410 | Gene |  |  |  |  |
| At4g23370 | Gene+Repeat | At4g13360 | Gene | At4g23320 | Gene |  |  |  |  |
| At4g25070 | Gene+Repeat | At4g18250 | Gene | At4g30020 | Gene |  |  |  |  |
| At4g20830 | Gene+Repeat | At4g15830 | Gene | At4g12700 | Gene |  |  |  |  |
| At4g31910 | Gene+Repeat | At4g25350 | Gene | At4g31410 | Gene |  |  |  |  |
| At4g39200 | Gene+Repeat | At4g32910 | Gene | At4g17130 | Gene |  |  |  |  |
| At4g27990 | Gene+Repeat | At4g25220 | Gene | At4g16320 | Gene |  |  |  |  |
| At4g00960 | Gene+Repeat | At4g09430 | Gene | At4g26610 | Gene |  |  |  |  |
| At4g00640 | Gene+Repeat | At4g04800 | Gene | At4g37670 | Gene |  |  |  |  |
| At4g09820 | Gene+Repeat | At4g25240 | Gene | At4g16420 | Gene |  |  |  |  |
| At4g34150 | Gene+Repeat | At4g25540 | Gene | At4g03570 | Gene |  |  |  |  |
| At4g18110 | Gene+Repeat | At4g01330 | Gene | At4g01100 | Gene |  |  |  |  |
| At4g08868 | Gene+Repeat | At4g38120 | Gene | At4g34040 | Gene |  |  |  |  |
| At4g04710 | Gene+Repeat | At4g26990 | Gene | At4g16295 | Gene |  |  |  |  |
| At4g23510 | Gene+Repeat | At4g29050 | Gene | At4g18790 | Gene |  |  |  |  |
| At4g34690 | Gene+Repeat | At4g20990 | Gene | At4g18020 | Gene |  |  |  |  |
| At4g31100 | Gene+Repeat | At4g25690 | Gene | At4g23560 | Gene |  |  |  |  |
| At4g02130 | Gene+Repeat | At4g29890 | Gene | At4g12000 | Gene |  |  |  |  |
| At4g13270 | Gene+Repeat | At4g29640 | Gene | At4g09740 | Gene |  |  |  |  |
| At4g23990 | Gene+Repeat | At4g12860 | Gene | At4g34310 | Gene |  |  |  |  |
| At4g33780 | Gene+Repeat | At4g01160 | Gene | At4g24175 | Gene |  |  |  |  |
| At4g11070 | Gene+Repeat | At4g09100 | Gene | At4g30662 | Gene |  |  |  |  |
|  |  | At4g02465 | Gene | At4g35420 | Gene |  |  |  |  |
|  |  | At4g05230 | Gene | At4g09730 | Gene |  |  |  |  |
|  |  | At4g02990 | Gene | At4g03230 | Gene |  |  |  |  |
|  |  | At4g33220 | Gene | At4g36850 | Gene |  |  |  |  |
|  |  | At4g38500 | Gene | At4g37760 | Gene |  |  |  |  |
|  |  | At4g28620 | Gene | At4g11380 | Gene |  |  |  |  |
|  |  | At4g01540 | Gene | At4g31440 | Gene |  |  |  |  |
|  |  | At4g18593 | Gene | At4g02510 | Gene |  |  |  |  |
|  |  | At4g26270 | Gene | At4g27350 | Gene |  |  |  |  |
|  |  | At4g21790 | Gene | At4g04350 | Gene |  |  |  |  |
|  |  | At4g16095 | Gene | At4g28706 | Gene |  |  |  |  |
|  |  | At4g27510 | Gene | At4g03000 | Gene |  |  |  |  |
|  |  | At4g36160 | Gene | At4g28510 | Gene |  |  |  |  |
|  |  | At4g09750 | Gene | At4g15810 | Gene |  |  |  |  |
|  |  | At4g02230 | Gene | At4g34110 | Gene |  |  |  |  |
|  |  | At4g00231 | Gene | At4g11330 | Gene |  |  |  |  |
|  |  | At4g27730 | Gene | At4g22070 | Gene |  |  |  |  |
|  |  | At4g22090 | Gene | At4g38440 | Gene |  |  |  |  |
|  |  | At4g17830 | Gene | At4g03510 | Gene |  |  |  |  |
|  |  | At4g10450 | Gene | At4g13780 | Gene |  |  |  |  |
|  |  | At4g03140 | Gene | At4g35270 | Gene |  |  |  |  |
|  |  | At4g30870 | Gene | At4g05040 | Gene |  |  |  |  |
|  |  | At4g10470 | Gene | At4g19990 | Gene |  |  |  |  |
|  |  | At4g02840 | Gene | At4g32300 | Gene |  |  |  |  |
|  |  | At4g01550 | Gene | At4g28660 | Gene |  |  |  |  |
|  |  | At4g15390 | Gene | At4g16200 | Gene |  |  |  |  |
|  |  | At4g39330 | Gene | At4g12620 | Gene |  |  |  |  |
|  |  | At4g31270 | Gene | At4g15230 | Gene |  |  |  |  |
|  |  | At4g38225 | Gene | At4g36480 | Gene |  |  |  |  |
|  |  | At4g37200 | Gene | At4g15560 | Gene |  |  |  |  |
|  |  | At4g30400 | Gene | At4g26100 | Gene |  |  |  |  |
|  |  | At4g21000 | Gene | At4g34200 | Gene |  |  |  |  |
|  |  | At4g15740 | Gene | At4g33240 | Gene |  |  |  |  |
|  |  | At4g00850 | Gene | At4g35970 | Gene |  |  |  |  |
|  |  | At4g13050 | Gene | At4g02900 | Gene |  |  |  |  |
|  |  | At4g10530 | Gene | At4g38040 | Gene |  |  |  |  |
|  |  | At4g09760 | Gene | At4g33480 | Gene |  |  |  |  |
|  |  | At4g18596 | Gene | At4g03430 | Gene |  |  |  |  |
|  |  | At4g33750 | Gene | At4g27430 | Gene |  |  |  |  |
|  |  | At4g16480 | Gene | At4g34820 | Gene |  |  |  |  |
|  |  | At4g20610 | Gene | At4g04740 | Gene |  |  |  |  |
|  |  | At4g08770 | Gene | At4g08960 | Gene |  |  |  |  |
|  |  | At4g39490 | Gene | At4g36070 | Gene |  |  |  |  |
|  |  | At4g23840 | Gene | At4g11820 | Gene |  |  |  |  |
|  |  | At4g09990 | Gene | At4g31340 | Gene |  |  |  |  |
|  |  | At4g27680 | Gene | At4g34350 | Gene |  |  |  |  |
|  |  | At4g30390 | Gene | At4g28640 | Gene |  |  |  |  |
|  |  | At4g13890 | Gene | At4g14790 | Gene |  |  |  |  |
|  |  | At4g20320 | Gene | At4g02400 | Gene |  |  |  |  |
|  |  | At4g18280 | Gene | At4g34070 | Gene |  |  |  |  |
|  |  | At4g12830 | Gene | At4g35830 | Gene |  |  |  |  |
|  |  | At4g29285 | Gene | At4g12610 | Gene |  |  |  |  |
|  |  | At4g32170 | Gene | At4g26700 | Gene |  |  |  |  |
|  |  | At4g28360 | Gene | At4g28820 | Gene |  |  |  |  |
|  |  | At4g20630 | Gene | At4g25360 | Gene |  |  |  |  |
|  |  | At4g02670 | Gene | At4g24880 | Gene |  |  |  |  |
|  |  | At4g11680 | Gene | At4g05420 | Gene |  |  |  |  |
|  |  | At4g13770 | Gene | At4g39420 | Gene |  |  |  |  |
|  |  | At4g36515 | Gene | At4g10670 | Gene |  |  |  |  |
|  |  | At4g35760 | Gene | At4g15110 | Gene |  |  |  |  |
|  |  | At4g18470 | Gene | At4g01730 | Gene |  |  |  |  |
|  |  | At4g26470 | Gene | At4g19250 | Gene |  |  |  |  |
|  |  | At4g01026 | Gene | At4g02730 | Gene |  |  |  |  |
|  |  | At4g10970 | Gene | At4g01030 | Gene |  |  |  |  |
|  |  | At4g08510 | Gene | At4g34090 | Gene |  |  |  |  |
|  |  | At4g15563 | Gene | At4g29060 | Gene |  |  |  |  |
|  |  | At4g27750 | Gene | At4g33400 | Gene |  |  |  |  |
|  |  | At4g29390 | Gene | At4g38050 | Gene |  |  |  |  |
|  |  | At4g26170 | Gene | At4g16144 | Gene |  |  |  |  |
|  |  | At4g20710 | Gene | At4g05160 | Gene |  |  |  |  |
|  |  | At4g19330 | Gene | At4g18300 | Gene |  |  |  |  |
|  |  | At4g38940 | Gene | At4g18820 | Gene |  |  |  |  |
|  |  | At4g26350 | Gene | At4g19040 | Gene |  |  |  |  |
|  |  | At4g35090 | Gene | At4g14590 | Gene |  |  |  |  |
|  |  | At4g19730 | Gene | At4g15540 | Gene |  |  |  |  |
|  |  | At4g12950 | Gene | At4g38380 | Gene |  |  |  |  |
|  |  | At4g10640 | Gene | At4g13330 | Gene |  |  |  |  |
|  |  | At4g13800 | Gene | At4g36580 | Gene |  |  |  |  |
|  |  | At4g19000 | Gene | At4g30720 | Gene |  |  |  |  |
|  |  | At4g11240 | Gene | At4g30620 | Gene |  |  |  |  |
|  |  | At4g06746 | Gene | At4g36650 | Gene |  |  |  |  |
|  |  | At4g26260 | Gene | At4g30920 | Gene |  |  |  |  |
|  |  | At4g15620 | Gene | At4g01660 | Gene |  |  |  |  |
|  |  | At4g14230 | Gene | At4g13760 | Gene |  |  |  |  |
|  |  | At4g15760 | Gene | At4g33680 | Gene |  |  |  |  |
|  |  | At4g37680 | Gene | At4g23980 | Gene |  |  |  |  |
|  |  | At4g02410 | Gene | At4g15010 | Gene |  |  |  |  |
|  |  | At4g25280 | Gene | At4g34460 | Gene |  |  |  |  |
|  |  | At4g18810 | Gene | At4g03440 | Gene |  |  |  |  |
|  |  | At4g01040 | Gene | At4g09580 | Gene |  |  |  |  |
|  |  | At4g13240 | Gene | At4g32320 | Gene |  |  |  |  |
|  |  | At4g15735 | Gene | At4g27760 | Gene |  |  |  |  |
|  |  | At4g21580 | Gene | At4g13980 | Gene |  |  |  |  |
|  |  | At4g17550 | Gene | At4g15236 | Gene |  |  |  |  |
|  |  | At4g24910 | Gene | At4g35880 | Gene |  |  |  |  |
|  |  | At4g15510 | Gene | At4g23920 | Gene |  |  |  |  |
|  |  | At4g29140 | Gene | At4g23500 | Gene |  |  |  |  |
|  |  | At4g17740 | Gene | At4g18060 | Gene |  |  |  |  |
|  |  | At4g27590 | Gene | At4g02930 | Gene |  |  |  |  |
|  |  | At4g28350 | Gene | At4g04370 | Gene |  |  |  |  |
|  |  | At4g17840 | Gene | At4g18150 | Gene |  |  |  |  |
|  |  | At4g00180 | Gene | At4g09020 | Gene |  |  |  |  |
|  |  | At4g13210 | Gene | At4g33590 | Gene |  |  |  |  |
|  |  | At4g18970 | Gene | At4g39430 | Gene |  |  |  |  |
|  |  | At4g03070 | Gene | At4g08930 | Gene |  |  |  |  |
|  |  | At4g23600 | Gene | At4g32120 | Gene |  |  |  |  |
|  |  | At4g29030 | Gene | At4g11420 | Gene |  |  |  |  |
|  |  | At4g22860 | Gene | At4g33420 | Gene |  |  |  |  |
|  |  | At4g11610 | Gene | At4g13060 | Gene |  |  |  |  |
|  |  | At4g11090 | Gene | At4g38470 | Gene |  |  |  |  |
|  |  | At4g11360 | Gene | At4g09140 | Gene |  |  |  |  |
|  |  | At4g23530 | Gene | At4g14970 | Gene |  |  |  |  |
|  |  | At4g10520 | Gene | At4g20620 | Gene |  |  |  |  |
|  |  | At4g36490 | Gene | At4g09040 | Gene |  |  |  |  |
|  |  | At4g05200 | Gene | At4g11260 | Gene |  |  |  |  |
|  |  | At4g12920 | Gene | At4g09130 | Gene |  |  |  |  |
|  |  | At4g00752 | Gene | At4g11230 | Gene |  |  |  |  |
|  |  | At4g17050 | Gene | At4g27420 | Gene |  |  |  |  |
|  |  | At4g34480 | Gene | At4g14605 | Gene |  |  |  |  |
|  |  | At4g23790 | Gene | At4g37870 | Gene |  |  |  |  |
|  |  | At4g12240 | Gene | At4g00900 | Gene |  |  |  |  |
|  |  | At4g05000 | Gene | At4g33760 | Gene |  |  |  |  |
|  |  | At4g10160 | Gene | At4g29910 | Gene |  |  |  |  |
|  |  | At4g33440 | Gene | At4g39960 | Gene |  |  |  |  |
|  |  | At4g17190 | Gene | At4g38230 | Gene |  |  |  |  |
|  |  | At4g11150 | Gene | At4g18270 | Gene |  |  |  |  |
|  |  | At4g09550 | Gene | At4g27070 | Gene |  |  |  |  |
|  |  | At4g22570 | Gene | At4g20600 | Gene |  |  |  |  |
|  |  | At4g19750 | Gene | At4g20235 | Gene |  |  |  |  |
|  |  | At4g28720 | Gene | At4g34280 | Gene |  |  |  |  |
|  |  | At4g00335 | Gene | At4g31120 | Gene |  |  |  |  |
|  |  | At4g20260 | Gene | At4g27690 | Gene |  |  |  |  |
|  |  | At4g31230 | Gene | At4g07380 | Gene |  |  |  |  |
|  |  | At4g09510 | Gene | At4g19010 | Gene |  |  |  |  |
|  |  | At4g32860 | Gene | At4g00380 | Gene |  |  |  |  |
|  |  | At4g14640 | Gene | At4g10660 | Gene |  |  |  |  |
|  |  | At4g10510 | Gene | At4g24280 | Gene |  |  |  |  |
|  |  | At4g13010 | Gene | At4g18750 | Gene |  |  |  |  |
|  |  | At4g16190 | Gene | At4g33490 | Gene |  |  |  |  |
|  |  | At4g21640 | Gene | At4g00540 | Gene |  |  |  |  |
|  |  | At4g29160 | Gene | At4g04080 | Gene |  |  |  |  |
|  |  | At4g34030 | Gene | At4g39210 | Gene |  |  |  |  |
|  |  | At4g18520 | Gene | At4g20360 | Gene |  |  |  |  |
|  |  | At4g28390 | Gene | At4g08810 | Gene |  |  |  |  |
|  |  | At4g02980 | Gene | At4g11120 | Gene |  |  |  |  |
|  |  | At4g30360 | Gene | At4g34890 | Gene |  |  |  |  |
|  |  | At4g15310 | Gene | At4g18440 | Gene |  |  |  |  |
|  |  | At4g12840 | Gene | At4g06676 | Gene |  |  |  |  |
|  |  | At4g20190 | Gene | At4g14800 | Gene |  |  |  |  |
|  |  | At4g14280 | Gene | At4g39520 | Gene |  |  |  |  |
|  |  | At4g13290 | Gene | At4g21600 | Gene |  |  |  |  |
|  |  | At4g39640 | Gene | At4g37360 | Gene |  |  |  |  |
|  |  | At4g03220 | Gene | At4g16155 | Gene |  |  |  |  |
|  |  | At4g20840 | Gene | At4g27100 | Gene |  |  |  |  |
|  |  | At4g38560 | Gene | At4g06490 | Gene |  |  |  |  |
|  |  | At4g04190 | Gene | At4g20430 | Gene |  |  |  |  |
|  |  | At4g25680 | Gene | At4g04670 | Gene |  |  |  |  |
|  |  | At4g20420 | Gene | At4g23820 | Gene |  |  |  |  |
|  |  | At4g04490 | Gene | At4g33520 | Gene |  |  |  |  |
|  |  | At4g20550 | Gene | At4g30100 | Gene |  |  |  |  |
|  |  | At4g05590 | Gene | At4g08940 | Gene |  |  |  |  |
|  |  | At4g20270 | Gene | At4g25970 | Gene |  |  |  |  |
|  |  | At4g25300 | Gene | At4g32430 | Gene |  |  |  |  |
|  |  | At4g34000 | Gene | At4g24830 | Gene |  |  |  |  |
|  |  | At4g14360 | Gene | At4g30530 | Gene |  |  |  |  |
|  |  | At4g10720 | Gene | At4g24970 | Gene |  |  |  |  |
|  |  | At4g10090 | Gene | At4g26420 | Gene |  |  |  |  |
|  |  | At4g03320 | Gene | At4g40100 | Gene |  |  |  |  |
|  |  | At4g18823 | Gene | At4g33670 | Gene |  |  |  |  |
|  |  | At4g05170 | Gene | At4g02480 | Gene |  |  |  |  |
|  |  | At4g19390 | Gene | At4g36390 | Gene |  |  |  |  |
|  |  | At4g16270 | Gene | At4g30510 | Gene |  |  |  |  |
|  |  | At4g39650 | Gene | At4g10960 | Gene |  |  |  |  |
|  |  | At4g15300 | Gene | At4g32050 | Gene |  |  |  |  |
|  |  | At4g16470 | Gene | At4g03490 | Gene |  |  |  |  |
|  |  | At4g00760 | Gene | At4g19180 | Gene |  |  |  |  |
|  |  | At4g14710 | Gene | At4g38890 | Gene |  |  |  |  |
|  |  | At4g20930 | Gene | At4g00690 | Gene |  |  |  |  |
|  |  | At4g20030 | Gene | At4g05330 | Gene |  |  |  |  |
|  |  | At4g14530 | Gene | At4g30990 | Gene |  |  |  |  |
|  |  | At4g26510 | Gene | At4g37270 | Gene |  |  |  |  |
|  |  | At4g04870 | Gene | At4g14940 | Gene |  |  |  |  |
|  |  | At4g00232 | Gene | At4g39570 | Gene |  |  |  |  |
|  |  | At4g32060 | Gene | At4g14930 | Gene |  |  |  |  |
|  |  | At4g24140 | Gene | At4g14500 | Gene |  |  |  |  |
|  |  | At4g20800 | Gene | At4g39240 | Gene |  |  |  |  |
|  |  | At4g39860 | Gene | At4g17120 | Gene |  |  |  |  |
|  |  | At4g28700 | Gene | At4g39110 | Gene |  |  |  |  |
|  |  | At4g36890 | Gene | At4g21270 | Gene |  |  |  |  |
|  |  | At4g02270 | Gene | At4g26180 | Gene |  |  |  |  |
|  |  | At4g00750 | Gene | At4g03190 | Gene |  |  |  |  |
|  |  | At4g15955 | Gene | At4g24900 | Gene |  |  |  |  |
|  |  | At4g32070 | Gene | At4g39690 | Gene |  |  |  |  |
|  |  | At4g05497 | Gene | At4g35335 | Gene |  |  |  |  |
|  |  | At4g37840 | Gene | At4g10620 | Gene |  |  |  |  |
|  |  | At4g01510 | Gene | At4g31810 | Gene |  |  |  |  |
|  |  | At4g01200 | Gene | At4g05210 | Gene |  |  |  |  |
|  |  | At4g28405 | Gene | At4g31590 | Gene |  |  |  |  |
|  |  | At4g28200 | Gene | At4g32820 | Gene |  |  |  |  |
|  |  | At4g07820 | Gene | At4g35890 | Gene |  |  |  |  |
|  |  | At4g24940 | Gene | At4g25515 | Gene |  |  |  |  |
|  |  | At4g30210 | Gene | At4g02010 | Gene |  |  |  |  |
|  |  | At4g30935 | Gene | At4g25840 | Gene |  |  |  |  |
|  |  | At4g22840 | Gene | At4g26550 | Gene |  |  |  |  |
|  |  | At4g14900 | Gene | At4g15720 | Gene |  |  |  |  |
|  |  | At4g00860 | Gene | At4g25030 | Gene |  |  |  |  |
|  |  | At4g24250 | Gene | At4g11670 | Gene |  |  |  |  |
|  |  | At4g28480 | Gene | At4g19490 | Gene |  |  |  |  |
|  |  | At4g00810 | Gene | At4g28550 | Gene |  |  |  |  |
|  |  | At4g10260 | Gene | At4g01830 | Gene |  |  |  |  |
|  |  | At4g24470 | Gene | At4g32180 | Gene |  |  |  |  |
|  |  | At4g28910 | Gene | At4g03590 | Gene |  |  |  |  |
|  |  | At4g02310 | Gene | At4g21540 | Gene |  |  |  |  |
|  |  | At4g27780 | Gene | At4g06479 | Gene |  |  |  |  |
|  |  | At4g34610 | Gene | At4g37100 | Gene |  |  |  |  |
|  |  | At4g19450 | Gene | At4g28950 | Gene |  |  |  |  |
|  |  | At4g08570 | Gene | At4g12030 | Gene |  |  |  |  |
|  |  | At4g22130 | Gene | At4g04920 | Gene |  |  |  |  |
|  |  | At4g14480 | Gene | At4g32840 | Gene |  |  |  |  |
|  |  | At4g30540 | Gene | At4g16850 | Gene |  |  |  |  |
|  |  | At4g18880 | Gene | At4g23620 | Gene |  |  |  |  |
|  |  | At4g09950 | Gene | At4g14950 | Gene |  |  |  |  |
|  |  | At4g02780 | Gene | At4g10050 | Gene |  |  |  |  |
|  |  | At4g01500 | Gene | At4g29360 | Gene |  |  |  |  |
|  |  | At4g37020 | Gene | At4g02710 | Gene |  |  |  |  |
|  |  | At4g27110 | Gene | At4g18050 | Gene |  |  |  |  |
|  |  | At4g28420 | Gene | At4g25160 | Gene |  |  |  |  |
|  |  | At4g02850 | Gene | At4g15880 | Gene |  |  |  |  |
|  |  | At4g28670 | Gene | At4g09150 | Gene |  |  |  |  |
|  |  | At4g39910 | Gene | At4g26620 | Gene |  |  |  |  |
|  |  | At4g23610 | Gene | At4g26590 | Gene |  |  |  |  |
|  |  | At4g01110 | Gene | At4g26920 | Gene |  |  |  |  |
|  |  | At4g17180 | Gene | At4g27640 | Gene |  |  |  |  |
|  |  | At4g13440 | Gene | At4g02790 | Gene |  |  |  |  |
|  |  | At4g00500 | Gene | At4g23000 | Gene |  |  |  |  |
|  |  | At4g32785 | Gene | At4g28260 | Gene |  |  |  |  |
|  |  | At4g20810 | Gene | At4g07400 | Gene |  |  |  |  |
|  |  | At4g37050 | Gene | At4g21390 | Gene |  |  |  |  |
|  |  | At4g01790 | Gene | At4g08593 | Gene |  |  |  |  |
|  |  | At4g09780 | Gene | At4g04940 | Gene |  |  |  |  |
|  |  | At4g38190 | Gene | At4g09680 | Gene |  |  |  |  |
|  |  | At4g01010 | Gene | At4g38430 | Gene |  |  |  |  |
|  |  | At4g30840 | Gene | At4g01850 | Gene |  |  |  |  |
|  |  | At4g11810 | Gene | At4g17270 | Gene |  |  |  |  |
|  |  | At4g20200 | Gene | At4g20980 | Gene |  |  |  |  |
|  |  | At4g38920 | Gene | At4g25960 | Gene |  |  |  |  |
|  |  | At4g03520 | Gene | At4g35520 | Gene |  |  |  |  |
|  |  | At4g03330 | Gene | At4g10790 | Gene |  |  |  |  |
|  |  | At4g12430 | Gene | At4g15730 | Gene |  |  |  |  |
|  |  | At4g34510 | Gene | At4g38170 | Gene |  |  |  |  |
|  |  | At4g27190 | Gene | At4g28010 | Gene |  |  |  |  |
|  |  | At4g16440 | Gene | At4g39952 | Gene |  |  |  |  |
|  |  | At4g24760 | Gene | At4g19860 | Gene |  |  |  |  |
|  |  | At4g21250 | Gene | At4g02020 | Gene |  |  |  |  |
|  |  | At4g30040 | Gene | At4g09340 | Gene |  |  |  |  |
|  |  | At4g38030 | Gene | At4g02640 | Gene |  |  |  |  |
|  |  | At4g10850 | Gene | At4g13400 | Gene |  |  |  |  |
|  |  | At4g24160 | Gene | At4g12710 | Gene |  |  |  |  |
|  |  | At4g14713 | Gene | At4g10940 | Gene |  |  |  |  |
|  |  | At4g02090 | Gene | At4g03390 | Gene |  |  |  |  |
|  |  | At4g33920 | Gene | At4g29400 | Gene |  |  |  |  |
|  |  | At4g19120 | Gene | At4g00290 | Gene |  |  |  |  |
|  |  | At4g08900 | Gene | At4g17895 | Gene |  |  |  |  |
|  |  | At4g23140 | Gene | At4g24520 | Gene |  |  |  |  |
|  |  | At4g17430 | Gene | At4g33090 | Gene |  |  |  |  |
|  |  | At4g03030 | Gene | At4g27080 | Gene |  |  |  |  |
|  |  | At4g08840 | Gene | At4g01210 | Gene |  |  |  |  |
|  |  | At4g29550 | Gene | At4g36440 | Gene |  |  |  |  |
|  |  | At4g16090 | Gene | At4g33380 | Gene |  |  |  |  |
|  |  | At4g14770 | Gene | At4g03110 | Gene |  |  |  |  |
|  |  | At4g17720 | Gene | At4g37880 | Gene |  |  |  |  |
|  |  | At4g29630 | Gene | At4g20040 | Gene |  |  |  |  |
|  |  | At4g15210 | Gene | At4g16180 | Gene |  |  |  |  |
|  |  | At4g23450 | Gene | At4g02860 | Gene |  |  |  |  |
|  |  | At4g01310 | Gene | At4g25450 | Gene |  |  |  |  |
|  |  | At4g01380 | Gene | At4g00700 | Gene |  |  |  |  |
|  |  | At4g30690 | Gene | At4g27480 | Gene |  |  |  |  |
|  |  | At4g39270 | Gene | At4g19210 | Gene |  |  |  |  |
|  |  | At4g13370 | Gene | At4g00660 | Gene |  |  |  |  |
|  |  | At4g12120 | Gene | At4g05460 | Gene |  |  |  |  |
|  |  | At4g19185 | Gene | At4g26760 | Gene |  |  |  |  |
|  |  | At4g14805 | Gene | At4g24680 | Gene |  |  |  |  |
|  |  | At4g24500 | Gene | At4g30490 | Gene |  |  |  |  |
|  |  | At4g22360 | Gene | At4g30560 | Gene |  |  |  |  |
|  |  | At4g14780 | Gene | At4g17020 | Gene |  |  |  |  |
|  |  | At4g16220 | Gene | At4g36940 | Gene |  |  |  |  |
|  |  | At4g04260 | Gene | At4g14070 | Gene |  |  |  |  |
|  |  | At4g18460 | Gene | At4g09620 | Gene |  |  |  |  |
|  |  | At4g01280 | Gene | At4g01860 | Gene |  |  |  |  |
|  |  | At4g19740 | Gene | At4g23430 | Gene |  |  |  |  |
|  |  | At4g22030 | Gene | At4g08210 | Gene |  |  |  |  |
|  |  | At4g26720 | Gene | At4g08590 | Gene |  |  |  |  |
|  |  | At4g37380 | Gene | At4g38370 | Gene |  |  |  |  |
|  |  | At4g20100 | Gene | At4g00330 | Gene |  |  |  |  |
|  |  | At4g23250 | Gene | At4g00710 | Gene |  |  |  |  |
|  |  | At4g22300 | Gene | At4g26410 | Gene |  |  |  |  |
|  |  | At4g14147 | Gene | At4g24630 | Gene |  |  |  |  |
|  |  | At4g05090 | Gene | At4g15900 | Gene |  |  |  |  |
|  |  | At4g28780 | Gene | At4g31790 | Gene |  |  |  |  |
|  |  | At4g22750 | Gene | At4g21323 | Gene |  |  |  |  |
|  |  | At4g37970 | Gene | At4g02820 | Gene |  |  |  |  |
|  |  | At4g37560 | Gene | At4g21170 | Gene |  |  |  |  |
|  |  | At4g09120 | Gene | At4g26370 | Gene |  |  |  |  |
|  |  | At4g01890 | Gene | At4g00340 | Gene |  |  |  |  |
|  |  | At4g34250 | Gene | At4g35130 | Gene |  |  |  |  |
|  |  | At4g00990 | Gene | At4g29730 | Gene |  |  |  |  |
|  |  | At4g30110 | Gene+Repeat | At4g00020 | Gene |  |  |  |  |
|  |  | At4g14700 | Gene+Repeat | At4g33650 | Gene |  |  |  |  |
|  |  | At4g21760 | Gene+Repeat | At4g33430 | Gene |  |  |  |  |
|  |  | At4g15430 | Gene+Repeat | At4g29790 | Gene |  |  |  |  |
|  |  | At4g13070 | Gene+Repeat | At4g11570 | Gene |  |  |  |  |
|  |  | At4g05020 | Gene+Repeat | At4g02350 | Gene |  |  |  |  |
|  |  | At4g23930 | Gene+Repeat | At4g01570 | Gene |  |  |  |  |
|  |  | At4g32360 | Gene+Repeat | At4g38200 | Gene |  |  |  |  |
|  |  | At4g16845 | Gene+Repeat | At4g26520 | Gene |  |  |  |  |
|  |  | At4g05540 | Gene+Repeat | At4g29540 | Gene |  |  |  |  |
|  |  | At4g28520 | Gene+Repeat | At4g34570 | Gene |  |  |  |  |
|  |  | At4g27595 | Gene+Repeat | At4g36910 | Gene |  |  |  |  |
|  |  | At4g28485 | Gene+Repeat | At4g22140 | Gene |  |  |  |  |
|  |  | At4g36170 | Gene+Repeat | At4g39850 | Gene |  |  |  |  |
|  |  | At4g38640 | Gene+Repeat | At4g31000 | Gene |  |  |  |  |
|  |  | At4g32375 | Gene+Repeat | At4g21060 | Gene |  |  |  |  |
|  |  | At4g21750 | Gene+Repeat | At4g38180 | Gene |  |  |  |  |
|  |  | At4g22780 | Gene+Repeat | At4g26910 | Gene |  |  |  |  |
|  |  | At4g15030 | Gene+Repeat | At4g36760 | Gene |  |  |  |  |
|  |  | At4g38770 | Gene+Repeat | At4g20340 | Gene |  |  |  |  |
|  |  | At4g24000 | Gene+Repeat | At4g01880 | Gene |  |  |  |  |
|  |  | At4g14220 | Gene+Repeat | At4g19500 | Gene |  |  |  |  |
|  |  | At4g32610 | Gene+Repeat | At4g37920 | Gene |  |  |  |  |
|  |  | At4g01610 | Gene+Repeat | At4g30030 | Gene |  |  |  |  |
|  |  | At4g10480 | Gene+Repeat | At4g30940 | Gene |  |  |  |  |
|  |  | At4g25390 | Gene+Repeat | At4g29900 | Gene |  |  |  |  |
|  |  | At4g39900 | Gene+Repeat | At4g09840 | Gene |  |  |  |  |
|  |  | At4g08360 | Gene+Repeat | At4g31480 | Gene |  |  |  |  |
|  |  | At4g32380 | Gene+Repeat | At4g31990 | Gene |  |  |  |  |
|  |  | At4g34440 | Gene+Repeat | At4g24020 | Gene |  |  |  |  |
|  |  | At4g15100 | Gene+Repeat | At4g15080 | Gene |  |  |  |  |
|  |  | At4g18550 | Gene+Repeat | At4g15040 | Gene |  |  |  |  |
|  |  | At4g26770 | Gene+Repeat | At4g12070 | Gene |  |  |  |  |
|  |  | At4g37510 | Gene+Repeat | At4g34100 | Gene |  |  |  |  |
|  |  | At4g17750 | Gene+Repeat | At4g09520 | Gene |  |  |  |  |
|  |  | At4g27850 | Gene+Repeat | At4g14240 | Gene |  |  |  |  |
|  |  | At4g16820 | Gene+Repeat | At4g15475 | Gene |  |  |  |  |
|  |  | At4g15980 | Gene+Repeat | At4g08460 | Gene |  |  |  |  |
|  |  | At4g13260 | Gene+Repeat | At4g02030 | Gene |  |  |  |  |
|  |  | At4g39000 | Gene+Repeat | At4g30160 | Gene |  |  |  |  |
|  |  | At4g31940 | Gene+Repeat | At4g35870 | Gene |  |  |  |  |
|  |  | At4g01080 | Gene+Repeat | At4g23850 | Gene |  |  |  |  |
|  |  | At4g01290 | Gene+Repeat | At4g28880 | Gene |  |  |  |  |
|  |  | At4g25670 | Gene+Repeat | At4g16310 | Gene |  |  |  |  |
|  |  | At4g03040 | Gene+Repeat | At4g14310 | Gene |  |  |  |  |
|  |  | At4g08240 | Gene+Repeat | At4g28020 | Gene |  |  |  |  |
|  |  | At4g15650 | Gene+Repeat | At4g31070 | Gene |  |  |  |  |
|  |  | At4g12560 | Gene+Repeat | At4g05523 | Gene |  |  |  |  |
|  |  | At4g36600 | Gene+Repeat | At4g33890 | Gene |  |  |  |  |
|  |  | At4g04570 | Gene+Repeat | At4g23300 | Gene |  |  |  |  |
|  |  | At4g38590 | Gene+Repeat | At4g30080 | Gene |  |  |  |  |
|  |  | At4g20070 | Gene+Repeat | At4g38130 | Gene |  |  |  |  |
|  |  | At4g12440 | Gene+Repeat | At4g09970 | Gene |  |  |  |  |
|  |  | At4g39400 | Gene+Repeat | At4g08180 | Gene |  |  |  |  |
|  |  | At4g21380 | Gene+Repeat | At4g37190 | Gene |  |  |  |  |
|  |  | At4g01780 | Gene+Repeat | At4g11830 | Gene |  |  |  |  |
|  |  | At4g09960 | Gene+Repeat | At4g21326 | Gene |  |  |  |  |
|  |  | At4g20900 | Gene+Repeat | At4g09210 | Gene |  |  |  |  |
|  |  | At4g08160 | Gene+Repeat | At4g21090 | Gene |  |  |  |  |
|  |  | At4g25320 | Gene+Repeat | At4g11840 | Gene |  |  |  |  |
|  |  | At4g37940 | Gene+Repeat | At4g20640 | Gene |  |  |  |  |
|  |  | At4g35295 | Gene+Repeat | At4g27610 | Gene |  |  |  |  |
|  |  | At4g04980 | Gene+Repeat | At4g17140 | Gene |  |  |  |  |
|  |  | At4g19260 | Gene+Repeat | At4g09470 | Gene |  |  |  |  |
|  |  | At4g02300 | Gene+Repeat | At4g12650 | Gene |  |  |  |  |
|  |  | At4g02280 | Gene+Repeat | At4g26000 | Gene |  |  |  |  |
|  |  | At4g14670 | Gene+Repeat | At4g02600 | Gene |  |  |  |  |
|  |  | At4g07825 | Gene+Repeat | At4g16170 | Gene |  |  |  |  |
|  |  | At4g37900 | Gene+Repeat | At4g32410 | Gene |  |  |  |  |
|  |  | At4g01595 | Gene+Repeat | At4g28470 | Gene |  |  |  |  |
|  |  | At4g27790 | Gene+Repeat | At4g36690 | Gene |  |  |  |  |
|  |  | At4g15550 | Gene+Repeat | At4g33530 | Gene |  |  |  |  |
|  |  | At4g00100 | Gene+Repeat | At4g30190 | Gene |  |  |  |  |
|  |  | At4g16045 | Gene+Repeat | At4g04320 | Gene |  |  |  |  |
|  |  | At4g05631 | Gene+Repeat | At4g24530 | Gene |  |  |  |  |
|  |  | At4g01440 | Gene+Repeat | At4g27020 | Gene |  |  |  |  |
|  |  | At4g11730 | Gene+Repeat | At4g02150 | Gene |  |  |  |  |
|  |  | At4g35930 | Gene+Repeat | At4g23940 | Gene |  |  |  |  |
|  |  | At4g29690 | Gene+Repeat | At4g08190 | Gene |  |  |  |  |
|  |  | At4g02570 | Gene+Repeat | At4g00590 | Gene |  |  |  |  |
|  |  | At4g34700 | Gene+Repeat | At4g36630 | Gene |  |  |  |  |
|  |  | At4g11400 | Gene+Repeat | At4g31420 | Gene |  |  |  |  |
|  |  | At4g32810 | Gene+Repeat | At4g00130 | Gene |  |  |  |  |
|  |  | At4g11430 | Gene+Repeat | At4g37440 | Gene |  |  |  |  |
|  |  | At4g14760 | Gene+Repeat | At4g37040 | Gene |  |  |  |  |
|  |  | At4g38320 | Gene+Repeat | At4g03480 | Gene |  |  |  |  |
|  |  | At4g00880 | Gene+Repeat | At4g00840 | Gene |  |  |  |  |
|  |  | At4g22890 | Gene+Repeat | At4g05120 | Gene |  |  |  |  |
|  |  | At4g14370 | Gene+Repeat | At4g25610 | Gene |  |  |  |  |
|  |  | At4g10180 | Gene+Repeat | At4g24580 | Gene |  |  |  |  |
|  |  | At4g28630 | Gene+Repeat | At4g15410 | Gene |  |  |  |  |
|  |  | At4g02580 | Gene+Repeat | At4g31490 | Gene |  |  |  |  |
|  |  | At4g10390 | Gene+Repeat | At4g31580 | Gene |  |  |  |  |
|  |  | At4g14250 | Gene+Repeat | At4g20580 | Gene |  |  |  |  |
|  |  | At4g11990 | Gene+Repeat | At4g29510 | Gene |  |  |  |  |
|  |  | At4g10595 | Gene+Repeat | At4g39350 | Gene |  |  |  |  |
|  |  | At4g23080 | Gene+Repeat | At4g29590 | Gene |  |  |  |  |
|  |  | At4g05470 | Gene+Repeat | At4g10770 | Gene |  |  |  |  |
|  |  | At4g13280 | Gene+Repeat | At4g39370 | Gene |  |  |  |  |
|  |  | At4g00350 | Gene+Repeat | At4g20080 | Gene |  |  |  |  |
|  |  | At4g05320 | Gene+Repeat | At4g01370 | Gene |  |  |  |  |
|  |  | At4g04510 | Gene+Repeat | At4g28920 | Gene |  |  |  |  |
|  |  | At4g27470 | Gene+Repeat | At4g38570 | Gene |  |  |  |  |
|  |  | At4g39380 | Gene+Repeat | At4g08040 | Gene |  |  |  |  |
|  |  | At4g18960 | Gene+Repeat | At4g18240 | Gene |  |  |  |  |
|  |  | At4g20130 | Gene+Repeat | At4g29130 | Gene |  |  |  |  |
|  |  | At4g26400 | Gene+Repeat | At4g19900 | Gene |  |  |  |  |
|  |  | At4g31820 | Gene+Repeat | At4g33410 | Gene |  |  |  |  |
|  |  | At4g17070 | Gene+Repeat | At4g13630 | Gene |  |  |  |  |
|  |  | At4g14090 | Gene+Repeat | At4g21890 | Gene |  |  |  |  |
|  |  | At4g14650 | Gene+Repeat | At4g13970 | Gene |  |  |  |  |
|  |  | At4g15755 | Gene+Repeat | At4g15090 | Gene |  |  |  |  |
|  |  | At4g25740 | Gene+Repeat | At4g13810 | Gene |  |  |  |  |
|  |  | At4g07940 | Gene+Repeat | At4g17230 | Gene |  |  |  |  |
|  |  | At4g38520 | Gene+Repeat | At4g28860 | Gene |  |  |  |  |
|  |  | At4g28690 | Gene+Repeat | At4g39390 | Gene |  |  |  |  |
|  |  | At4g18180 | Gene+Repeat | At4g10570 | Gene |  |  |  |  |
|  |  | At4g04480 | Gene+Repeat | At4g26940 | Gene |  |  |  |  |
|  |  | At4g01090 | Gene+Repeat | At4g01800 | Gene |  |  |  |  |
|  |  | At4g26540 | Gene+Repeat | At4g23540 | Gene |  |  |  |  |
|  |  | At4g01985 | Gene+Repeat | At4g38950 | Gene |  |  |  |  |
|  |  | At4g10695 | Gene+Repeat | At4g04460 | Gene |  |  |  |  |
|  |  | At4g21120 | Gene+Repeat | At4g27060 | Gene |  |  |  |  |
|  |  | At4g26120 | Gene+Repeat | At4g01810 | Gene |  |  |  |  |
|  |  | At4g20690 | Gene+Repeat | At4g07390 | Gene |  |  |  |  |
|  |  | At4g17060 | Gene+Repeat | At4g09980 | Gene |  |  |  |  |
|  |  | At4g09795 | Gene+Repeat | At4g21770 | Gene |  |  |  |  |
|  |  | At4g34830 | Gene+Repeat | At4g39460 | Gene |  |  |  |  |
|  |  | At4g19170 | Gene+Repeat | At4g11740 | Gene |  |  |  |  |
|  |  | At4g16740 | Gene+Repeat | At4g18160 | Gene |  |  |  |  |
|  |  | At4g08150 | Gene+Repeat | At4g32770 | Gene |  |  |  |  |
|  |  | At4g35240 | Gene+Repeat | At4g28380 | Gene |  |  |  |  |
|  |  | At4g15160 | Gene+Repeat | At4g14455 | Gene |  |  |  |  |
|  |  | At4g03060 | Gene+Repeat | At4g29880 | Gene |  |  |  |  |
|  |  | At4g11100 | Gene+Repeat | At4g36195 | Gene |  |  |  |  |
|  |  | At4g09300 | Gene+Repeat | At4g16835 | Gene |  |  |  |  |
|  |  | At4g16980 | Gene+Repeat | At4g35360 | Gene |  |  |  |  |
|  |  | At4g19440 | Gene+Repeat | At4g28540 | Gene |  |  |  |  |
|  |  | At4g14400 | Gene+Repeat | At4g09070 | Gene |  |  |  |  |
|  |  | At4g21800 | Gene+Repeat | At4g16250 | Gene |  |  |  |  |
|  |  | At4g17950 | Gene+Repeat | At4g24730 | Gene |  |  |  |  |
|  |  | At4g02075 | Gene+Repeat | At4g23650 | Gene |  |  |  |  |
|  |  | At4g04220 | Gene+Repeat | At4g00200 | Gene |  |  |  |  |
|  |  | At4g01680 | Gene+Repeat | At4g03415 | Gene |  |  |  |  |
|  |  | At4g10140 | Gene+Repeat | At4g00620 | Gene |  |  |  |  |
|  |  | At4g04840 | Gene+Repeat | At4g28760 | Gene |  |  |  |  |
|  |  | At4g29020 | Gene+Repeat | At4g04790 | Gene |  |  |  |  |
|  |  | At4g12250 | Gene+Repeat | At4g19540 | Gene |  |  |  |  |
|  |  | At4g33970 | Gene+Repeat | At4g12570 | Gene |  |  |  |  |
|  |  | At4g23030 | Gene+Repeat | At4g13320 | Gene |  |  |  |  |
|  |  | At4g19970 | Gene+Repeat | At4g31050 | Gene |  |  |  |  |
|  |  | At4g04990 | Gene+Repeat | At4g36730 | Gene |  |  |  |  |
|  |  | At4g10360 | Gene+Repeat | At4g17420 | Gene |  |  |  |  |
|  |  | At4g10860 | Gene+Repeat | At4g12130 | Gene |  |  |  |  |
|  |  |  |  | At4g34370 | Gene |  |  |  |  |
|  |  |  |  | At4g02080 | Gene |  |  |  |  |
|  |  |  |  | At4g27900 | Gene |  |  |  |  |
|  |  |  |  | At4g19220 | Gene |  |  |  |  |
|  |  |  |  | At4g15850 | Gene |  |  |  |  |
|  |  |  |  | At4g34120 | Gene |  |  |  |  |
|  |  |  |  | At4g03080 | Gene |  |  |  |  |
|  |  |  |  | At4g28070 | Gene |  |  |  |  |
|  |  |  |  | At4g08520 | Gene |  |  |  |  |
|  |  |  |  | At4g11980 | Gene |  |  |  |  |
|  |  |  |  | At4g16143 | Gene |  |  |  |  |
|  |  |  |  | At4g19510 | Gene |  |  |  |  |
|  |  |  |  | At4g08260 | Gene |  |  |  |  |
|  |  |  |  | At4g14200 | Gene |  |  |  |  |
|  |  |  |  | At4g24490 | Gene |  |  |  |  |
|  |  |  |  | At4g10955 | Gene |  |  |  |  |
|  |  |  |  | At4g22760 | Gene |  |  |  |  |
|  |  |  |  | At4g39450 | Gene |  |  |  |  |
|  |  |  |  | At4g18975 | Gene |  |  |  |  |
|  |  |  |  | At4g21530 | Gene |  |  |  |  |
|  |  |  |  | At4g13030 | Gene |  |  |  |  |
|  |  |  |  | At4g02680 | Gene |  |  |  |  |
|  |  |  |  | At4g20410 | Gene |  |  |  |  |
|  |  |  |  | At4g05520 | Gene |  |  |  |  |
|  |  |  |  | At4g26980 | Gene |  |  |  |  |
|  |  |  |  | At4g27120 | Gene |  |  |  |  |
|  |  |  |  | At4g14340 | Gene |  |  |  |  |
|  |  |  |  | At4g21940 | Gene |  |  |  |  |
|  |  |  |  | At4g24800 | Gene |  |  |  |  |
|  |  |  |  | At4g00450 | Gene |  |  |  |  |
|  |  |  |  | At4g36280 | Gene |  |  |  |  |
|  |  |  |  | At4g30350 | Gene |  |  |  |  |
|  |  |  |  | At4g31850 | Gene |  |  |  |  |
|  |  |  |  | At4g33200 | Gene |  |  |  |  |
|  |  |  |  | At4g26050 | Gene |  |  |  |  |
|  |  |  |  | At4g21550 | Gene |  |  |  |  |
|  |  |  |  | At4g09010 | Gene |  |  |  |  |
|  |  |  |  | At4g16215 | Gene |  |  |  |  |
|  |  |  |  | At4g14290 | Gene |  |  |  |  |
|  |  |  |  | At4g19710 | Gene |  |  |  |  |
|  |  |  |  | At4g32640 | Gene |  |  |  |  |
|  |  |  |  | At4g10430 | Gene |  |  |  |  |
|  |  |  |  | At4g12010 | Gene |  |  |  |  |
|  |  |  |  | At4g16120 | Gene |  |  |  |  |
|  |  |  |  | At4g30900 | Gene |  |  |  |  |
|  |  |  |  | At4g28715 | Gene |  |  |  |  |
|  |  |  |  | At4g32700 | Gene |  |  |  |  |
|  |  |  |  | At4g00520 | Gene |  |  |  |  |
|  |  |  |  | At4g13650 | Gene |  |  |  |  |
|  |  |  |  | At4g29990 | Gene |  |  |  |  |
|  |  |  |  | At4g04690 | Gene |  |  |  |  |
|  |  |  |  | At4g30310 | Gene |  |  |  |  |
|  |  |  |  | At4g28830 | Gene |  |  |  |  |
|  |  |  |  | At4g29960 | Gene |  |  |  |  |
|  |  |  |  | At4g15233 | Gene |  |  |  |  |
|  |  |  |  | At4g23060 | Gene |  |  |  |  |
|  |  |  |  | At4g08895 | Gene |  |  |  |  |
|  |  |  |  | At4g09640 | Gene |  |  |  |  |
|  |  |  |  | At4g33990 | Gene |  |  |  |  |
|  |  |  |  | At4g25290 | Gene |  |  |  |  |
|  |  |  |  | At4g05060 | Gene |  |  |  |  |
|  |  |  |  | At4g38270 | Gene |  |  |  |  |
|  |  |  |  | At4g03090 | Gene |  |  |  |  |
|  |  |  |  | At4g11850 | Gene |  |  |  |  |
|  |  |  |  | At4g31540 | Gene |  |  |  |  |
|  |  |  |  | At4g12680 | Gene |  |  |  |  |
|  |  |  |  | At4g11530 | Gene |  |  |  |  |
|  |  |  |  | At4g08250 | Gene |  |  |  |  |
|  |  |  |  | At4g11800 | Gene |  |  |  |  |
|  |  |  |  | At4g00910 | Gene |  |  |  |  |
|  |  |  |  | At4g04930 | Gene |  |  |  |  |
|  |  |  |  | At4g16015 | Gene |  |  |  |  |
|  |  |  |  | At4g19560 | Gene |  |  |  |  |
|  |  |  |  | At4g34240 | Gene |  |  |  |  |
|  |  |  |  | At4g14300 | Gene |  |  |  |  |
|  |  |  |  | At4g32270 | Gene |  |  |  |  |
|  |  |  |  | At4g10700 | Gene |  |  |  |  |
|  |  |  |  | At4g37120 | Gene |  |  |  |  |
|  |  |  |  | At4g13590 | Gene |  |  |  |  |
|  |  |  |  | At4g04720 | Gene |  |  |  |  |
|  |  |  |  | At4g04610 | Gene |  |  |  |  |
|  |  |  |  | At4g36460 | Gene |  |  |  |  |
|  |  |  |  | At4g15530 | Gene |  |  |  |  |
|  |  |  |  | At4g38350 | Gene |  |  |  |  |
|  |  |  |  | At4g21230 | Gene |  |  |  |  |
|  |  |  |  | At4g28220 | Gene |  |  |  |  |
|  |  |  |  | At4g39620 | Gene |  |  |  |  |
|  |  |  |  | At4g04880 | Gene |  |  |  |  |
|  |  |  |  | At4g08470 | Gene |  |  |  |  |
|  |  |  |  | At4g18120 | Gene |  |  |  |  |
|  |  |  |  | At4g16195 | Gene |  |  |  |  |
|  |  |  |  | At4g11410 | Gene |  |  |  |  |
|  |  |  |  | At4g21670 | Gene |  |  |  |  |
|  |  |  |  | At4g00970 | Gene |  |  |  |  |
|  |  |  |  | At4g24620 | Gene |  |  |  |  |
|  |  |  |  | At4g23420 | Gene |  |  |  |  |
|  |  |  |  | At4g28980 | Gene |  |  |  |  |
|  |  |  |  | At4g18465 | Gene |  |  |  |  |
|  |  |  |  | At4g16670 | Gene |  |  |  |  |
|  |  |  |  | At4g23100 | Gene |  |  |  |  |
|  |  |  |  | At4g00800 | Gene |  |  |  |  |
|  |  |  |  | At4g30520 | Gene |  |  |  |  |
|  |  |  |  | At4g16390 | Gene |  |  |  |  |
|  |  |  |  | At4g27650 | Gene |  |  |  |  |
|  |  |  |  | At4g27630 | Gene |  |  |  |  |
|  |  |  |  | At4g24270 | Gene |  |  |  |  |
|  |  |  |  | At4g31150 | Gene |  |  |  |  |
|  |  |  |  | At4g18130 | Gene |  |  |  |  |
|  |  |  |  | At4g16430 | Gene |  |  |  |  |
|  |  |  |  | At4g03960 | Gene |  |  |  |  |
|  |  |  |  | At4g11130 | Gene |  |  |  |  |
|  |  |  |  | At4g08790 | Gene |  |  |  |  |
|  |  |  |  | At4g21300 | Gene |  |  |  |  |
|  |  |  |  | At4g31240 | Gene |  |  |  |  |
|  |  |  |  | At4g01450 | Gene |  |  |  |  |
|  |  |  |  | At4g09660 | Gene |  |  |  |  |
|  |  |  |  | At4g22930 | Gene |  |  |  |  |
|  |  |  |  | At4g01690 | Gene |  |  |  |  |
|  |  |  |  | At4g29920 | Gene |  |  |  |  |
|  |  |  |  | At4g21650 | Gene |  |  |  |  |
|  |  |  |  | At4g36720 | Gene |  |  |  |  |
|  |  |  |  | At4g19960 | Gene |  |  |  |  |
|  |  |  |  | At4g19090 | Gene |  |  |  |  |
|  |  |  |  | At4g34660 | Gene |  |  |  |  |
|  |  |  |  | At4g13020 | Gene |  |  |  |  |
|  |  |  |  | At4g33495 | Gene |  |  |  |  |
|  |  |  |  | At4g21700 | Gene |  |  |  |  |
|  |  |  |  | At4g31390 | Gene |  |  |  |  |
|  |  |  |  | At4g30000 | Gene |  |  |  |  |
|  |  |  |  | At4g08290 | Gene |  |  |  |  |
|  |  |  |  | At4g02120 | Gene |  |  |  |  |
|  |  |  |  | At4g31170 | Gene |  |  |  |  |
|  |  |  |  | At4g38780 | Gene |  |  |  |  |
|  |  |  |  | At4g17300 | Gene |  |  |  |  |
|  |  |  |  | At4g18780 | Gene |  |  |  |  |
|  |  |  |  | At4g19660 | Gene |  |  |  |  |
|  |  |  |  | At4g23895 | Gene |  |  |  |  |
|  |  |  |  | At4g17150 | Gene |  |  |  |  |
|  |  |  |  | At4g08230 | Gene |  |  |  |  |
|  |  |  |  | At4g19080 | Gene |  |  |  |  |
|  |  |  |  | At4g28770 | Gene |  |  |  |  |
|  |  |  |  | At4g24560 | Gene |  |  |  |  |
|  |  |  |  | At4g20870 | Gene |  |  |  |  |
|  |  |  |  | At4g29010 | Gene |  |  |  |  |
|  |  |  |  | At4g03020 | Gene |  |  |  |  |
|  |  |  |  | At4g11160 | Gene |  |  |  |  |
|  |  |  |  | At4g32620 | Gene |  |  |  |  |
|  |  |  |  | At4g15056 | Gene |  |  |  |  |
|  |  |  |  | At4g31060 | Gene |  |  |  |  |
|  |  |  |  | At4g28850 | Gene |  |  |  |  |
|  |  |  |  | At4g09080 | Gene |  |  |  |  |
|  |  |  |  | At4g11060 | Gene |  |  |  |  |
|  |  |  |  | At4g04885 | Gene |  |  |  |  |
|  |  |  |  | At4g31300 | Gene |  |  |  |  |
|  |  |  |  | At4g30130 | Gene |  |  |  |  |
|  |  |  |  | At4g30060 | Gene |  |  |  |  |
|  |  |  |  | At4g35780 | Gene |  |  |  |  |
|  |  |  |  | At4g24390 | Gene |  |  |  |  |
|  |  |  |  | At4g20540 | Gene |  |  |  |  |
|  |  |  |  | At4g00630 | Gene |  |  |  |  |
|  |  |  |  | At4g00580 | Gene |  |  |  |  |
|  |  |  |  | At4g39050 | Gene |  |  |  |  |
|  |  |  |  | At4g00550 | Gene |  |  |  |  |
|  |  |  |  | At4g12750 | Gene |  |  |  |  |
|  |  |  |  | At4g16660 | Gene |  |  |  |  |
|  |  |  |  | At4g35910 | Gene |  |  |  |  |
|  |  |  |  | At4g17090 | Gene |  |  |  |  |
|  |  |  |  | At4g16970 | Gene |  |  |  |  |
|  |  |  |  | At4g31140 | Gene |  |  |  |  |
|  |  |  |  | At4g00755 | Gene |  |  |  |  |
|  |  |  |  | At4g12590 | Gene |  |  |  |  |
|  |  |  |  | At4g14990 | Gene |  |  |  |  |
|  |  |  |  | At4g01820 | Gene |  |  |  |  |
|  |  |  |  | At4g14840 | Gene |  |  |  |  |
|  |  |  |  | At4g31770 | Gene |  |  |  |  |
|  |  |  |  | At4g08540 | Gene |  |  |  |  |
|  |  |  |  | At4g20590 | Gene |  |  |  |  |
|  |  |  |  | At4g14510 | Gene |  |  |  |  |
|  |  |  |  | At4g35500 | Gene |  |  |  |  |
|  |  |  |  | At4g32010 | Gene |  |  |  |  |
|  |  |  |  | At4g37030 | Gene |  |  |  |  |
|  |  |  |  | At4g29480 | Gene |  |  |  |  |
|  |  |  |  | At4g31090 | Gene |  |  |  |  |
|  |  |  |  | At4g31880 | Gene |  |  |  |  |
|  |  |  |  | At4g10590 | Gene |  |  |  |  |
|  |  |  |  | At4g01020 | Gene |  |  |  |  |
|  |  |  |  | At4g21160 | Gene |  |  |  |  |
|  |  |  |  | At4g32600 | Gene |  |  |  |  |
|  |  |  |  | At4g16760 | Gene |  |  |  |  |
|  |  |  |  | At4g03250 | Gene |  |  |  |  |
|  |  |  |  | At4g00720 | Gene |  |  |  |  |
|  |  |  |  | At4g34910 | Gene |  |  |  |  |
|  |  |  |  | At4g00370 | Gene |  |  |  |  |
|  |  |  |  | At4g30630 | Gene |  |  |  |  |
|  |  |  |  | At4g25340 | Gene |  |  |  |  |
|  |  |  |  | At4g02460 | Gene |  |  |  |  |
|  |  |  |  | At4g38550 | Gene |  |  |  |  |
|  |  |  |  | At4g32790 | Gene |  |  |  |  |
|  |  |  |  | At4g26440 | Gene |  |  |  |  |
|  |  |  |  | At4g14170 | Gene |  |  |  |  |
|  |  |  |  | At4g16400 | Gene |  |  |  |  |
|  |  |  |  | At4g01670 | Gene |  |  |  |  |
|  |  |  |  | At4g09250 | Gene |  |  |  |  |
|  |  |  |  | At4g40050 | Gene |  |  |  |  |
|  |  |  |  | At4g19610 | Gene |  |  |  |  |
|  |  |  |  | At4g14880 | Gene |  |  |  |  |
|  |  |  |  | At4g34270 | Gene |  |  |  |  |
|  |  |  |  | At4g16100 | Gene |  |  |  |  |
|  |  |  |  | At4g32970 | Gene+Repeat |  |  |  |  |
|  |  |  |  | At4g21660 | Gene+Repeat |  |  |  |  |
|  |  |  |  | At4g25880 | Gene+Repeat |  |  |  |  |
|  |  |  |  | At4g29950 | Gene+Repeat |  |  |  |  |
|  |  |  |  | At4g24770 | Gene+Repeat |  |  |  |  |
|  |  |  |  | At4g02920 | Gene+Repeat |  |  |  |  |
|  |  |  |  | At4g33030 | Gene+Repeat |  |  |  |  |
|  |  |  |  | At4g04540 | Gene+Repeat |  |  |  |  |
|  |  |  |  | At4g18770 | Gene+Repeat |  |  |  |  |
|  |  |  |  | At4g11660 | Gene+Repeat |  |  |  |  |
|  |  |  |  | At4g16680 | Gene+Repeat |  |  |  |  |
|  |  |  |  | At4g12670 | Gene+Repeat |  |  |  |  |
|  |  |  |  | At4g02740 | Gene+Repeat |  |  |  |  |
|  |  |  |  | At4g15290 | Gene+Repeat |  |  |  |  |
|  |  |  |  | At4g26870 | Gene+Repeat |  |  |  |  |
|  |  |  |  | At4g00060 | Gene+Repeat |  |  |  |  |
|  |  |  |  | At4g09670 | Gene+Repeat |  |  |  |  |
|  |  |  |  | At4g09560 | Gene+Repeat |  |  |  |  |
|  |  |  |  | At4g11050 | Gene+Repeat |  |  |  |  |
|  |  |  |  | At4g22280 | Gene+Repeat |  |  |  |  |
|  |  |  |  | At4g38600 | Gene+Repeat |  |  |  |  |
|  |  |  |  | At4g21630 | Gene+Repeat |  |  |  |  |
|  |  |  |  | At4g16630 | Gene+Repeat |  |  |  |  |
|  |  |  |  | At4g30710 | Gene+Repeat |  |  |  |  |
|  |  |  |  | At4g11720 | Gene+Repeat |  |  |  |  |
|  |  |  |  | At4g36770 | Gene+Repeat |  |  |  |  |
|  |  |  |  | At4g36270 | Gene+Repeat |  |  |  |  |
|  |  |  |  | At4g22420 | Gene+Repeat |  |  |  |  |
|  |  |  |  | At4g16950 | Gene+Repeat |  |  |  |  |
|  |  |  |  | At4g12460 | Gene+Repeat |  |  |  |  |
|  |  |  |  | At4g20920 | Gene+Repeat |  |  |  |  |
|  |  |  |  | At4g23460 | Gene+Repeat |  |  |  |  |
|  |  |  |  | At4g09360 | Gene+Repeat |  |  |  |  |
|  |  |  |  | At4g25730 | Gene+Repeat |  |  |  |  |
|  |  |  |  | At4g20850 | Gene+Repeat |  |  |  |  |
|  |  |  |  | At4g13550 | Gene+Repeat |  |  |  |  |
|  |  |  |  | At4g10330 | Gene+Repeat |  |  |  |  |
|  |  |  |  | At4g26600 | Gene+Repeat |  |  |  |  |
|  |  |  |  | At4g26690 | Gene+Repeat |  |  |  |  |
|  |  |  |  | At4g29000 | Gene+Repeat |  |  |  |  |
|  |  |  |  | At4g28600 | Gene+Repeat |  |  |  |  |
|  |  |  |  | At4g01650 | Gene+Repeat |  |  |  |  |
|  |  |  |  | At4g39820 | Gene+Repeat |  |  |  |  |
|  |  |  |  | At4g10845 | Gene+Repeat |  |  |  |  |
|  |  |  |  | At4g10020 | Gene+Repeat |  |  |  |  |
|  |  |  |  | At4g36520 | Gene+Repeat |  |  |  |  |
|  |  |  |  | At4g26800 | Gene+Repeat |  |  |  |  |
|  |  |  |  | At4g07666 | Gene+Repeat |  |  |  |  |
|  |  |  |  | At4g35230 | Gene+Repeat |  |  |  |  |
|  |  |  |  | At4g31160 | Gene+Repeat |  |  |  |  |
|  |  |  |  | At4g01240 | Gene+Repeat |  |  |  |  |
|  |  |  |  | At4g33630 | Gene+Repeat |  |  |  |  |
|  |  |  |  | At4g27585 | Gene+Repeat |  |  |  |  |
|  |  |  |  | At4g13920 | Gene+Repeat |  |  |  |  |
|  |  |  |  | At4g38760 | Gene+Repeat |  |  |  |  |
|  |  |  |  | At4g34430 | Gene+Repeat |  |  |  |  |
|  |  |  |  | At4g04970 | Gene+Repeat |  |  |  |  |
|  |  |  |  | At4g03120 | Gene+Repeat |  |  |  |  |
|  |  |  |  | At4g02750 | Gene+Repeat |  |  |  |  |
|  |  |  |  | At4g31180 | Gene+Repeat |  |  |  |  |
|  |  |  |  | At4g14965 | Gene+Repeat |  |  |  |  |
|  |  |  |  | At4g23590 | Gene+Repeat |  |  |  |  |
|  |  |  |  | At4g10060 | Gene+Repeat |  |  |  |  |
|  |  |  |  | At4g09880 | Gene+Repeat |  |  |  |  |
|  |  |  |  | At4g35790 | Gene+Repeat |  |  |  |  |
|  |  |  |  | At4g11110 | Gene+Repeat |  |  |  |  |
|  |  |  |  | At4g26190 | Gene+Repeat |  |  |  |  |
|  |  |  |  | At4g33500 | Gene+Repeat |  |  |  |  |
|  |  |  |  | At4g23860 | Gene+Repeat |  |  |  |  |
|  |  |  |  | At4g37480 | Gene+Repeat |  |  |  |  |
|  |  |  |  | At4g12770 | Gene+Repeat |  |  |  |  |
|  |  |  |  | At4g32250 | Gene+Repeat |  |  |  |  |
|  |  |  |  | At4g10840 | Gene+Repeat |  |  |  |  |
|  |  |  |  | At4g39180 | Gene+Repeat |  |  |  |  |
|  |  |  |  | At4g16920 | Gene+Repeat |  |  |  |  |
|  |  |  |  | At4g15940 | Gene+Repeat |  |  |  |  |
|  |  |  |  | At4g16110 | Gene+Repeat |  |  |  |  |
|  |  |  |  | At4g27010 | Gene+Repeat |  |  |  |  |
|  |  |  |  | At4g34320 | Gene+Repeat |  |  |  |  |
|  |  |  |  | At4g08280 | Gene+Repeat |  |  |  |  |
|  |  |  |  | At4g10030 | Gene+Repeat |  |  |  |  |
|  |  |  |  | At4g22370 | Gene+Repeat |  |  |  |  |
|  |  |  |  | At4g20910 | Gene+Repeat |  |  |  |  |
|  |  |  |  | At4g10170 | Gene+Repeat |  |  |  |  |
|  |  |  |  | At4g24190 | Gene+Repeat |  |  |  |  |
|  |  |  |  | At4g09000 | Gene+Repeat |  |  |  |  |
|  |  |  |  | At4g08870 | Gene+Repeat |  |  |  |  |
|  |  |  |  | At4g39680 | Gene+Repeat |  |  |  |  |
|  |  |  |  | At4g02660 | Gene+Repeat |  |  |  |  |
|  |  |  |  | At4g27500 | Gene+Repeat |  |  |  |  |
|  |  |  |  | At4g26480 | Gene+Repeat |  |  |  |  |
|  |  |  |  | At4g09220 | Gene+Repeat |  |  |  |  |
|  |  |  |  | At4g39080 | Gene+Repeat |  |  |  |  |
|  |  |  |  | At4g20090 | Gene+Repeat |  |  |  |  |
|  |  |  |  | At4g29560 | Gene+Repeat |  |  |  |  |
|  |  |  |  | At4g27940 | Gene+Repeat |  |  |  |  |
|  |  |  |  | At4g16570 | Gene+Repeat |  |  |  |  |
|  |  |  |  | At4g11790 | Gene+Repeat |  |  |  |  |
|  |  |  |  | At4g18690 | Gene+Repeat |  |  |  |  |
|  |  |  |  | At4g17770 | Gene+Repeat |  |  |  |  |
|  |  |  |  | At4g32850 | Gene+Repeat |  |  |  |  |
|  |  |  |  | At4g01520 | Gene+Repeat |  |  |  |  |
|  |  |  |  | At4g13390 | Gene+Repeat |  |  |  |  |
|  |  |  |  | At4g10120 | Gene+Repeat |  |  |  |  |
|  |  |  |  | At4g08320 | Gene+Repeat |  |  |  |  |
|  |  |  |  | At4g23740 | Gene+Repeat |  |  |  |  |
|  |  |  |  | At4g10740 | Gene+Repeat |  |  |  |  |
|  |  |  |  | At4g15215 | Gene+Repeat |  |  |  |  |
|  |  |  |  | At4g08580 | Gene+Repeat |  |  |  |  |
|  |  |  |  | At4g17990 | Gene+Repeat |  |  |  |  |
|  |  |  |  | At4g16940 | Gene+Repeat |  |  |  |  |
|  |  |  |  | At4g08390 | Gene+Repeat |  |  |  |  |
|  |  |  |  | At4g20170 | Gene+Repeat |  |  |  |  |
|  |  |  |  | At4g39280 | Gene+Repeat |  |  |  |  |
|  |  |  |  | At4g32520 | Gene+Repeat |  |  |  |  |
|  |  |  |  | At4g08690 | Gene+Repeat |  |  |  |  |
|  |  |  |  | At4g15570 | Gene+Repeat |  |  |  |  |
|  |  |  |  | At4g19110 | Gene+Repeat |  |  |  |  |
|  |  |  |  | At4g03560 | Gene+Repeat |  |  |  |  |
|  |  |  |  | At4g27380 | Gene+Repeat |  |  |  |  |
|  |  |  |  | At4g08670 | Gene+Repeat |  |  |  |  |
|  |  |  |  | At4g30830 | Gene+Repeat |  |  |  |  |
|  |  |  |  | At4g38750 | Gene+Repeat |  |  |  |  |
|  |  |  |  | At4g22670 | Gene+Repeat |  |  |  |  |
|  |  |  |  | At4g19600 | Gene+Repeat |  |  |  |  |
|  |  |  |  | At4g35470 | Gene+Repeat |  |  |  |  |
|  |  |  |  | At4g32160 | Gene+Repeat |  |  |  |  |
|  |  |  |  | At4g21430 | Gene+Repeat |  |  |  |  |
|  |  |  |  | At4g31670 | Gene+Repeat |  |  |  |  |
|  |  |  |  | At4g05190 | Gene+Repeat |  |  |  |  |
|  |  |  |  | At4g37820 | Gene+Repeat |  |  |  |  |
|  |  |  |  | At4g34740 | Gene+Repeat |  |  |  |  |
|  |  |  |  | At4g25520 | Gene+Repeat |  |  |  |  |
|  |  |  |  | At4g04340 | Gene+Repeat |  |  |  |  |
|  |  |  |  | At4g07410 | Gene+Repeat |  |  |  |  |
|  |  |  |  | At4g20160 | Gene+Repeat |  |  |  |  |
|  |  |  |  | At4g25120 | Gene+Repeat |  |  |  |  |
|  |  |  |  | At4g02700 | Gene+Repeat |  |  |  |  |
|  |  |  |  | At4g23470 | Gene+Repeat |  |  |  |  |
|  |  |  |  | At4g05360 | Gene+Repeat |  |  |  |  |
|  |  |  |  | At4g35940 | Gene+Repeat |  |  |  |  |
|  |  |  |  | At4g08550 | Gene+Repeat |  |  |  |  |
|  |  |  |  | At4g25400 | Gene+Repeat |  |  |  |  |
|  |  |  |  | At4g32760 | Gene+Repeat |  |  |  |  |
|  |  |  |  | At4g02110 | Gene+Repeat |  |  |  |  |
|  |  |  |  | At4g08780 | Gene+Repeat |  |  |  |  |
|  |  |  |  | At4g31350 | Gene+Repeat |  |  |  |  |
|  |  |  |  | At4g19670 | Gene+Repeat |  |  |  |  |
|  |  |  |  | At4g15320 | Gene+Repeat |  |  |  |  |
|  |  |  |  | At4g24170 | Gene+Repeat |  |  |  |  |
|  |  |  |  | At4g00830 | Gene+Repeat |  |  |  |  |
|  |  |  |  | At4g04020 | Gene+Repeat |  |  |  |  |
|  |  |  |  | At4g36180 | Gene+Repeat |  |  |  |  |
|  |  |  |  | At4g15790 | Gene+Repeat |  |  |  |  |
|  |  |  |  | At4g08380 | Gene+Repeat |  |  |  |  |
|  |  |  |  | At4g12020 | Gene+Repeat |  |  |  |  |
|  |  |  |  | At4g21150 | Gene+Repeat |  |  |  |  |
|  |  |  |  | At4g11340 | Gene+Repeat |  |  |  |  |
|  |  |  |  | At4g08850 | Gene+Repeat |  |  |  |  |
|  |  |  |  | At4g05410 | Gene+Repeat |  |  |  |  |
|  |  |  |  | At4g19050 | Gene+Repeat |  |  |  |  |
|  |  |  |  | At4g39235 | Gene+Repeat |  |  |  |  |
|  |  |  |  | At4g13460 | Gene+Repeat |  |  |  |  |
|  |  |  |  | At4g30750 | Gene+Repeat |  |  |  |  |
|  |  |  |  | At4g32730 | Gene+Repeat |  |  |  |  |
|  |  |  |  | At4g31570 | Gene+Repeat |  |  |  |  |
|  |  |  |  | At4g36290 | Gene+Repeat |  |  |  |  |
|  |  |  |  | At4g35140 | Gene+Repeat |  |  |  |  |
|  |  |  |  | At4g37590 | Gene+Repeat |  |  |  |  |
|  |  |  |  | At4g31900 | Gene+Repeat |  |  |  |  |
|  |  |  |  | At4g02220 | Gene+Repeat |  |  |  |  |
|  |  |  |  | At4g08455 | Gene+Repeat |  |  |  |  |
|  |  |  |  | At4g08480 | Gene+Repeat |  |  |  |  |
|  |  |  |  | At4g34140 | Gene+Repeat |  |  |  |  |
|  |  |  |  | At4g04450 | Gene+Repeat |  |  |  |  |
|  |  |  |  | At4g04110 | Gene+Repeat |  |  |  |  |
|  |  |  |  | At4g30910 | Gene+Repeat |  |  |  |  |
|  |  |  |  | At4g27620 | Gene+Repeat |  |  |  |  |
|  |  |  |  | At4g18030 | Gene+Repeat |  |  |  |  |
|  |  |  |  | At4g29380 | Gene+Repeat |  |  |  |  |
|  |  |  |  | At4g15840 | Gene+Repeat |  |  |  |  |
|  |  |  |  | At4g10070 | Gene+Repeat |  |  |  |  |
|  |  |  |  | At4g15820 | Gene+Repeat |  |  |  |  |
|  |  |  |  | At4g08620 | Gene+Repeat |  |  |  |  |
|  |  |  |  | At4g26630 | Gene+Repeat |  |  |  |  |
|  |  |  |  | At4g30970 | Gene+Repeat |  |  |  |  |
|  |  |  |  | At4g31430 | Gene+Repeat |  |  |  |  |
|  |  |  |  | At4g28990 | Gene+Repeat |  |  |  |  |
|  |  |  |  | At4g07524 | Gene+Repeat |  |  |  |  |
|  |  |  |  | At4g08370 | Gene+Repeat |  |  |  |  |
|  |  |  |  | At4g25500 | Gene+Repeat |  |  |  |  |
|  |  |  |  | At4g28080 | Gene+Repeat |  |  |  |  |
|  |  |  |  | At4g04890 | Gene+Repeat |  |  |  |  |
|  |  |  |  | At4g32551 | Gene+Repeat |  |  |  |  |
|  |  |  |  | At4g10080 | Gene+Repeat |  |  |  |  |
|  |  |  |  | At4g24100 | Gene+Repeat |  |  |  |  |
|  |  |  |  | At4g36790 | Gene+Repeat |  |  |  |  |
|  |  |  |  | At4g33470 | Gene+Repeat |  |  |  |  |
|  |  |  |  | At4g21710 | Gene+Repeat |  |  |  |  |
|  |  |  |  | At4g13350 | Gene+Repeat |  |  |  |  |
|  |  |  |  | At4g08980 | Gene+Repeat |  |  |  |  |
|  |  |  |  | At4g26970 | Gene+Repeat |  |  |  |  |
|  |  |  |  | At4g29750 | Gene+Repeat |  |  |  |  |
|  |  |  |  | At4g14150 | Gene+Repeat |  |  |  |  |
|  |  |  |  | At4g01340 | Gene+Repeat |  |  |  |  |
|  |  |  |  | At4g14140 | Gene+Repeat |  |  |  |  |
|  |  |  |  | At4g04760 | Gene+Repeat |  |  |  |  |
|  |  |  |  | At4g37170 | Gene+Repeat |  |  |  |  |
|  |  |  |  | At4g16890 | Gene+Repeat |  |  |  |  |
|  |  |  |  | At4g13880 | Gene+Repeat |  |  |  |  |
|  |  |  |  | At4g27040 | Gene+Repeat |  |  |  |  |
|  |  |  |  | At4g04850 | Gene+Repeat |  |  |  |  |
|  |  |  |  | At4g02260 | Gene+Repeat |  |  |  |  |
|  |  |  |  | At4g02760 | Gene+Repeat |  |  |  |  |
|  |  |  |  | At4g13750 | Gene+Repeat |  |  |  |  |
|  |  |  |  | At4g02430 | Gene+Repeat |  |  |  |  |
|  |  |  |  | At4g04180 | Gene+Repeat |  |  |  |  |
|  |  |  |  | At4g08310 | Gene+Repeat |  |  |  |  |
|  |  |  |  | At4g37130 | Gene+Repeat |  |  |  |  |
|  |  |  |  | At4g32650 | Gene+Repeat |  |  |  |  |
|  |  |  |  | At4g19060 | Gene+Repeat |  |  |  |  |
|  |  |  |  | At4g31040 | Gene+Repeat |  |  |  |  |
|  |  |  |  | At4g19240 | Gene+Repeat |  |  |  |  |
|  |  |  |  | At4g03180 | Gene+Repeat |  |  |  |  |
|  |  |  |  | At4g34900 | Gene+Repeat |  |  |  |  |
|  |  |  |  | At4g29440 | Gene+Repeat |  |  |  |  |
|  |  |  |  | At4g21180 | Gene+Repeat |  |  |  |  |
|  |  |  |  | At4g39030 | Gene+Repeat |  |  |  |  |
|  |  |  |  | At4g26430 | Gene+Repeat |  |  |  |  |
|  |  |  |  | At4g08990 | Gene+Repeat |  |  |  |  |
|  |  |  |  | At4g36140 | Gene+Repeat |  |  |  |  |
|  |  |  |  | At4g03260 | Gene+Repeat |  |  |  |  |
|  |  |  |  | At4g00930 | Gene+Repeat |  |  |  |  |
|  |  |  |  | At4g12640 | Gene+Repeat |  |  |  |  |
|  |  |  |  | At4g15890 | Gene+Repeat |  |  |  |  |
|  |  |  |  | At4g14160 | Gene+Repeat |  |  |  |  |
|  |  |  |  | At4g01320 | Gene+Repeat |  |  |  |  |
|  |  |  |  | At4g24290 | Gene+Repeat |  |  |  |  |
|  |  |  |  | At4g04330 | Gene+Repeat |  |  |  |  |
|  |  |  |  | At4g38240 | Gene+Repeat |  |  |  |  |
|  |  |  |  | At4g23330 | Gene+Repeat |  |  |  |  |
|  |  |  |  | At4g08170 | Gene+Repeat |  |  |  |  |
|  |  |  |  | At4g19650 | Gene+Repeat |  |  |  |  |
|  |  |  |  | At4g27030 | Gene+Repeat |  |  |  |  |
|  |  |  |  | At4g05553 | Gene+Repeat |  |  |  |  |
|  |  |  |  | At4g07990 | Gene+Repeat |  |  |  |  |
|  |  |  |  | At4g01130 | Gene+Repeat |  |  |  |  |
|  |  |  |  | At4g09180 | Gene+Repeat |  |  |  |  |
|  |  |  |  | At4g14850 | Gene+Repeat |  |  |  |  |
|  |  |  |  | At4g31750 | Gene+Repeat |  |  |  |  |
|  |  |  |  | At4g23180 | Gene+Repeat |  |  |  |  |
|  |  |  |  | At4g32560 | Gene+Repeat |  |  |  |  |
|  |  |  |  | At4g13420 | Gene+Repeat |  |  |  |  |
|  |  |  |  | At4g19020 | Gene+Repeat |  |  |  |  |
|  |  |  |  | At4g36860 | Gene+Repeat |  |  |  |  |
|  |  |  |  | At4g19590 | Gene+Repeat |  |  |  |  |
|  |  |  |  | At4g08450 | Gene+Repeat |  |  |  |  |
|  |  |  |  | At4g13430 | Gene+Repeat |  |  |  |  |
|  |  |  |  | At4g11440 | Gene+Repeat |  |  |  |  |
|  |  |  |  | At4g10930 | Gene+Repeat |  |  |  |  |
|  |  |  |  | At4g01400 | Gene+Repeat |  |  |  |  |
|  |  |  |  | At4g17610 | Gene+Repeat |  |  |  |  |
|  |  |  |  | At4g07932 | Gene+Repeat |  |  |  |  |
|  |  |  |  | At4g15440 | Gene+Repeat |  |  |  |  |
|  |  |  |  | At4g09720 | Gene+Repeat |  |  |  |  |
|  |  |  |  | At4g17410 | Gene+Repeat |  |  |  |  |
|  |  |  |  | At4g08410 | Gene+Repeat |  |  |  |  |
|  |  |  |  | At4g28450 | Gene+Repeat |  |  |  |  |
|  |  |  |  | At4g13610 | Gene+Repeat |  |  |  |  |
|  |  |  |  | At4g12270 | Gene+Repeat |  |  |  |  |
|  |  |  |  | At4g20740 | Gene+Repeat |  |  |  |  |
|  |  |  |  | At4g21900 | Gene+Repeat |  |  |  |  |
|  |  |  |  | At4g24610 | Gene+Repeat |  |  |  |  |
|  |  |  |  | At4g16960 | Gene+Repeat |  |  |  |  |
|  |  |  |  | At4g30150 | Gene+Repeat |  |  |  |  |
|  |  |  |  | At4g10550 | Gene+Repeat |  |  |  |  |
|  |  |  |  | At4g26450 | Gene+Repeat |  |  |  |  |
|  |  |  |  | At4g03200 | Gene+Repeat |  |  |  |  |
|  |  |  |  | At4g33300 | Gene+Repeat |  |  |  |  |
|  |  |  |  | At4g39160 | Gene+Repeat |  |  |  |  |
|  |  |  |  | At4g14520 | Gene+Repeat |  |  |  |  |
|  |  |  |  | At4g32420 | Gene+Repeat |  |  |  |  |
|  |  |  |  | At4g10730 | Gene+Repeat |  |  |  |  |
|  |  |  |  | At4g39170 | Gene+Repeat |  |  |  |  |
|  |  |  |  | At4g09630 | Gene+Repeat |  |  |  |  |
|  |  |  |  | At4g25180 | Gene+Repeat |  |  |  |  |
|  |  |  |  | At4g22540 | Gene+Repeat |  |  |  |  |
|  |  |  |  | At4g11470 | Gene+Repeat |  |  |  |  |
|  |  |  |  | At4g04200 | Gene+Repeat |  |  |  |  |
|  |  |  |  | At4g16280 | Gene+Repeat |  |  |  |  |
|  |  |  |  | At4g08500 | Gene+Repeat |  |  |  |  |
|  |  |  |  | At4g25530 | Gene+Repeat |  |  |  |  |
|  |  |  |  | At4g11920 | Gene+Repeat |  |  |  |  |
|  |  |  |  | At4g08330 | Gene+Repeat |  |  |  |  |
|  |  |  |  | At4g10710 | Gene+Repeat |  |  |  |  |
|  |  |  |  | At4g10300 | Gene+Repeat |  |  |  |  |
|  |  |  |  | At4g17080 | Gene+Repeat |  |  |  |  |
|  |  |  |  | At4g07960 | Gene+Repeat |  |  |  |  |
|  |  |  |  | At4g22730 | Gene+Repeat |  |  |  |  |
|  |  |  |  | At4g24750 | Gene+Repeat |  |  |  |  |
|  |  |  |  | At4g12780 | Gene+Repeat |  |  |  |  |
|  |  |  |  | At4g20210 | Gene+Repeat |  |  |  |  |
|  |  |  |  | At4g15096 | Gene+Repeat |  |  |  |  |
|  |  |  |  | At4g33290 | Gene+Repeat |  |  |  |  |
|  |  |  |  | At4g00300 | Gene+Repeat |  |  |  |  |
|  |  |  |  | At4g07950 | Gene+Repeat |  |  |  |  |
|  |  |  |  | At4g35800 | Gene+Repeat |  |  |  |  |
|  |  |  |  | At4g36400 | Gene+Repeat |  |  |  |  |
|  |  |  |  | At4g31200 | Gene+Repeat |  |  |  |  |
|  |  |  |  | At4g18375 | Gene+Repeat |  |  |  |  |
|  |  |  |  | At4g26110 | Gene+Repeat |  |  |  |  |
|  |  |  |  | At4g37640 | Gene+Repeat |  |  |  |  |
|  |  |  |  | At4g06526 | Gene+Repeat |  |  |  |  |
|  |  |  |  | At4g22290 | Gene+Repeat |  |  |  |  |
|  |  |  |  | At4g35785 | Gene+Repeat |  |  |  |  |
|  |  |  |  | At4g19190 | Gene+Repeat |  |  |  |  |
|  |  |  |  | At4g33620 | Gene+Repeat |  |  |  |  |
|  |  |  |  | At4g28710 | Gene+Repeat |  |  |  |  |
|  |  |  |  | At4g03580 | Gene+Repeat |  |  |  |  |
|  |  |  |  | At4g02880 | Gene+Repeat |  |  |  |  |
|  |  |  |  | At4g05530 | Gene+Repeat |  |  |  |  |
|  |  |  |  | At4g15180 | Gene+Repeat |  |  |  |  |
|  |  |  |  | At4g23440 | Gene+Repeat |  |  |  |  |
|  |  |  |  | At4g11560 | Gene+Repeat |  |  |  |  |
|  |  |  |  | At4g05632 | Gene+Repeat |  |  |  |  |
|  |  |  |  | At4g36090 | Gene+Repeat |  |  |  |  |
|  |  |  |  | At4g10350 | Gene+Repeat |  |  |  |  |
|  |  |  |  | At4g08395 | Gene+Repeat |  |  |  |  |
|  |  |  |  | At4g14210 | Gene+Repeat |  |  |  |  |
|  |  |  |  | At4g23910 | Gene+Repeat |  |  |  |  |
|  |  |  |  | At4g12230 | Gene+Repeat |  |  |  |  |
|  |  |  |  | At4g08400 | Gene+Repeat |  |  |  |  |
|  |  |  |  | At4g36120 | Gene+Repeat |  |  |  |  |
|  |  |  |  | At4g08350 | Gene+Repeat |  |  |  |  |
|  |  |  |  | At4g36360 | Gene+Repeat |  |  |  |  |
|  |  |  |  | At4g03380 | Gene+Repeat |  |  |  |  |
|  |  |  |  | At4g36980 | Gene+Repeat |  |  |  |  |
|  |  |  |  | At4g21810 | Gene+Repeat |  |  |  |  |
|  |  |  |  | At4g00230 | Gene+Repeat |  |  |  |  |
|  |  |  |  | At4g31520 | Gene+Repeat |  |  |  |  |
|  |  |  |  | At4g21880 | Gene+Repeat |  |  |  |  |
|  |  |  |  | At4g00570 | Gene+Repeat |  |  |  |  |
|  |  |  |  | At4g22740 | Gene+Repeat |  |  |  |  |
|  |  |  |  | At4g18600 | Gene+Repeat |  |  |  |  |
|  |  |  |  | At4g22940 | Gene+Repeat |  |  |  |  |
|  |  |  |  | At4g16070 | Gene+Repeat |  |  |  |  |
|  |  |  |  | At4g03950 | Gene+Repeat |  |  |  |  |
|  |  |  |  | At4g34930 | Gene+Repeat |  |  |  |  |
|  |  |  |  | At4g11970 | Gene+Repeat |  |  |  |  |
|  |  |  |  | At4g12740 | Gene+Repeat |  |  |  |  |
|  |  |  |  | At4g20720 | Gene+Repeat |  |  |  |  |
|  |  |  |  | At4g00650 | Gene+Repeat |  |  |  |  |
|  |  |  |  | At4g08300 | Gene+Repeat |  |  |  |  |
|  |  |  |  | At4g02070 | Gene+Repeat |  |  |  |  |
|  |  |  |  | At4g18710 | Gene+Repeat |  |  |  |  |
|  |  |  |  | At4g14920 | Gene+Repeat |  |  |  |  |
