# Supplementary material for: Epigenomic Consequences of Immortalized Plant Cell Suspension Culture
Source: PLoS Biol. 2008 Dec 9;6(12):e302. doi: 10.1371/journal.pbio.0060302 (PMC2596858; doi:10.1371/journal.pbio.0060302)
Supplement: Supplementary file 9 [file pbio.0060302.st003.doc]

**Tanurdzic et al., Supplemental Table S3**

**Gene**

At4g00750

At4g02270

At4g02670

At4g02780

At4g03330

At4g04210

At4g04460

At4g04630

At4g04790

At4g05140

At4g05230

At4g05330

At4g07820

At4g08840

At4g09100

At4g09130

At4g09430

At4g09460

At4g11030

At4g12920

At4g13210

At4g13760

At4g18010

At4g19330

At4g19840

At4g20100

At4g20200

At4g20530

At4g22570

At4g26420

At4g30140
